# Antibacterial Siderophores of *Pandoraea* Pathogens and Their Impact on the Diseased Lung Microbiota

**DOI:** 10.1002/anie.202505714

**Published:** 2025-04-14

**Authors:** Elena Herzog, Keishi Ishida, Kirstin Scherlach, Xiuqiang Chen, Benjamin Bartels, Sarah P. Niehs, Bachar Cheaib, Gianni Panagiotou, Christian Hertweck

**Affiliations:** ^1^ Department of Biomolecular Chemistry Leibniz Institute for Natural Product Research and Infection Biology (HKI) Beutenbergstraße 11a 07745 Jena Germany; ^2^ Department of Microbiome Dynamics Leibniz Institute for Natural Product Research and Infection Biology (HKI) Beutenbergstraße 11a 07745 Jena Germany; ^3^ Department of Infectious Diseases, Medical Microbiology and Hygiene Medical Faculty Heidelberg University Im Neuenheimer Feld 672 69120 Heidelberg Germany; ^4^ Faculty of Biological Sciences Friedrich Schiller University Jena Jena 07743 Germany; ^5^ Department of Medicine The University of Hong Kong Hong Kong SAR 999999 China; ^6^ Natural Product Chemistry, Faculty of Biological Sciences Friedrich Schiller University Jena Jena 07743 Germany; ^7^ Cluster of Excellence Balance in the Microverse Friedrich Schiller University Jena Fürstengraben 1 07743 Jena Germany

**Keywords:** Genome mining, Natural products, Nonribosomal peptide synthetases, Siderophores, Structure elucidation

## Abstract

Antibiotic‐resistant bacteria of the genus *Pandoraea*, frequently acquired from the environment, are an emerging cause of opportunistic respiratory infections, especially in cystic fibrosis (CF) patients. However, their specialized metabolites, including niche and virulence factors, remained unknown. Through genome mining of environmental and clinical isolates of diverse *Pandoraea* species, we identified a highly conserved biosynthesis gene cluster (*pan*) that codes for a nonribosomal peptide synthetase (NRPS) assembling a new siderophore. Using bioinformatics‐guided metabolic profiling of wild type and a targeted null mutant, we discovered the corresponding metabolites, pandorabactin A and B. Their structures and chelate (gallium) complexes were elucidated by a combination of chemical degradation, derivatization, NMR, and MS analysis. Metagenomics and bioinformatics of sputum samples of CF patients indicated that the presence of the *pan* gene locus correlates with the prevalence of specific bacteria in the lung microbiome. Bioassays and mass spectrometry imaging showed that pandorabactins have antibacterial activities against various lung pathogens (*Pseudomonas*, *Mycobacterium*, and *Stenotrophomonas*) through depleting iron in the competitors. Taken together, these findings offer first insight into niche factors of *Pandoraea* and indicate that pandorabactins shape the diseased lung microbiota through the competition for iron.

## Graphical abstract

Genome mining of environmental and pathogenic *Pandoraea* species identified a conserved NRPS gene cluster and led to the discovery of novel siderophores, pandorabactins, which exert their antibacterial activity by depleting iron from competitors. Bioinformatic analysis of sputum from cystic fibrosis patients coupled with bioassays indicates that pandorabactins outcompete other bacteria in sequestering iron and have an impact on the diseased lung microbiota.

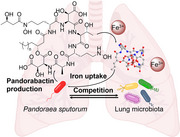

## Introduction

According to Greek mythology, all of humanity's evils were released when Pandora opened a large storage jar containing diseases, illnesses, and various other plagues. The newly established genus *Pandoraea*, which comprises a number of Gram‐negative bacterial species, refers to this myth.^[^ ^1^, ^2^, ^3^ ^]^ In fact, *Pandoraea* species are problematic because they are widespread in the environment, often cause severe respiratory infections, and are resistant to commonly used antibiotics, such as β‐lactams, quinolones, cephalosporins, and aminoglycosides.^[^ ^4^, ^5^, ^6^ ^]^ In particular, these pathogens pose a risk to patients suffering from cystic fibrosis (CF), one of the most common genetic diseases.^[^ ^7^ ^]^ The main characteristic of CF is the accumulation of sticky mucus in the lungs, which promotes chronic infections. Progressive tissue damage eventually leads to respiratory failure and death of the patients.^[^ ^8^ ^]^ Typically, the various bacterial pathogens infecting CF patients are acquired from environmental reservoirs or through patient‐to‐patient transmission.^[^ ^9^ ^]^ It is alarming that an increasing number of sputa from CF patients contain *Pandoraea* species.^[^ ^10^, ^11^ ^]^ In addition, recent reports indicate that *Pandoraea* spp. can also cause severe infections and sepsis in non‐CF patients, causing numerous outbreaks in intensive care units.^[^ ^12^, ^13^, ^14^, ^15^, ^16^ ^]^ Given the emergence of *Pandoraea* strains as pathogens causing severe respiratory disease and sepsis, it is striking that practically nothing is known about their potential niche and virulence factors.^[^ ^10^ ^]^

For pathogens, the ability to efficiently capture iron is essential for their survival in the lung.^[^ ^17^, ^18^, ^19^ ^]^ Therefore, understanding how this essential element is acquired by pathogenic bacteria may provide a basis for new therapeutic approaches to treat CF infections.^[^ ^20^, ^21^, ^22^ ^]^ Microorganisms have evolved sophisticated ways of extracting iron from the environment using low molecular weight chelators, such as siderophores, with high affinity for ferric ions.^[^ ^23^ ^]^ While there is a substantial body of knowledge on siderophores produced by a multitude of environmental and pathogenic bacteria, the genus *Pandoraea* is unexplored in this regard.^[^ ^19^ ^]^ In a previous study, a putative metallophore was detected by mass spectrometric analysis of a *Pandoraea* sp. culture, but the compound was not identified, and no data were shown.^[^ ^24^ ^]^

Here, we report the genomics‐driven discovery of a specialized *Pandoraea* biosynthetic gene cluster (BGC) for a siderophore assembly line that is conserved in environmental and pathogenic species and can also be detected in the metagenomes of patient sputum samples. We describe the successful isolation and full characterization of two siderophore congeners, the nonribosomal peptides pandorabactin A and B, and provide a model of the iron complex. On the basis of bioinformatics and mutational analysis of the native producer, we put forward the biosynthetic steps leading to the formation of both acylated cyclic peptides. Finally, we demonstrate that pandorabactins have antibacterial activity against co‐inhabitants of the lungs of CF patients.

## Results and Discussion

**Figure 1:**
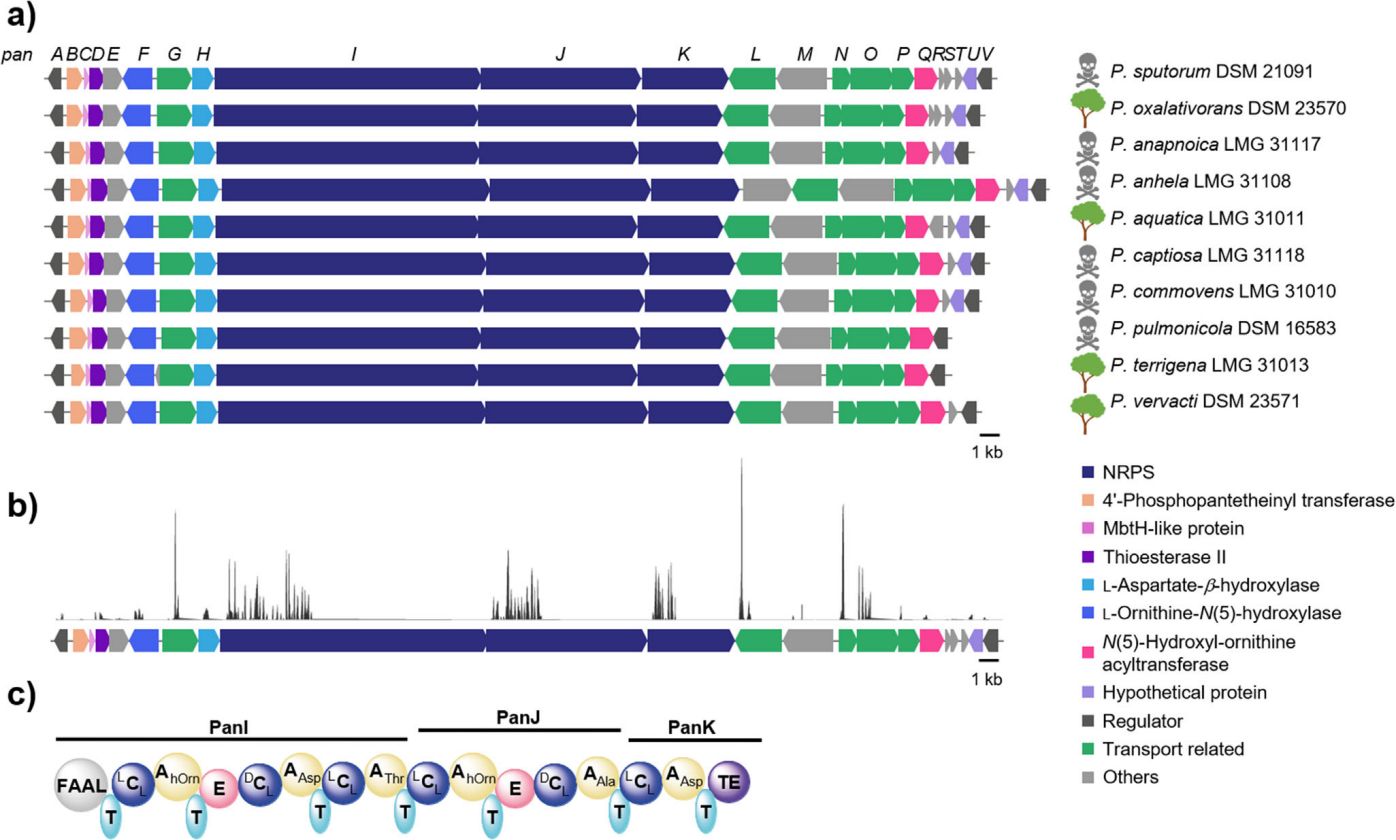
Genome mining for pandorabactin biosynthetic gene clusters and metagenomic analysis. a) Highly conserved *pan* biosynthetic gene clusters coding for NRPS, 4′‐phosphopantetheinyl transferase, MbtH‐like protein, thioesterase type II, l‐ornithine‐*N*(5)‐hydroxylase, l‐aspartate‐β‐hydroxylase, *N*(5)‐hydroxyl‐ornithine acetyltransferase, transporters, and regulators in *Pandoraea* strains, with the skull representing pathogenic strains from CF patients and the tree representing strains from the environment. b) Read coverage of short‐read sequencing of *P. sputorum pan* BGC. c) Assembly line deduced from the *pan* NRPS genes. FAAL, fatty acyl‐AMP ligase domain; C, condensation domain; T, thiolation domain; A, adenylation domain; E, epimerization domain; and TE, thioesterase domain.

To investigate the biosynthetic potential of *Pandoraea* spp. for the production of siderophores, we mined publicly available genome sequences for genes encoding proteins that are required for siderophore biosynthesis and transport. Specifically, we searched for nonribosomal peptide synthetase (NRPS) gene clusters with TonB receptor and/or iron ABC transporter genes. In this way, we discovered a gene locus (named *pan*) that appeared to be conserved in environmental and pathogenic *Pandoraea* isolates (Figure 1a). In addition to the TonB receptor gene (*panL*) and other transport‐related genes, we detected genes in the vicinity that encode a multimodular NRPS and various other biosynthesis enzymes, including a tentative dioxygenase (PanH). Using the *panH* sequence as a bioinformatic handle, we conducted a genome neighborhood analysis with the EFI‐GNT tool.^[^ ^25^ ^]^ The results indicated that the *pan* gene locus is highly conserved in genomes of numerous environmental and pathogenic *Pandoraea* strains (Figure 1a). Notably, these include clinical isolates from CF patients, namely *Pandoraea anapnoica*, *Pandoraea commovens*, *Pandoraea anhela*, *Pandoraea pulmonicola*, and *Pandoraea captiosa*.^[^ ^26^ ^]^

To investigate whether the *pan*‐BGC is indeed present in the diseased lung microbiota, we analyzed shotgun metagenomic sequencing data from 40 sputum samples of CF patients.^[^ ^27^ ^]^ Despite the challenge of low biomass in sputum samples, we succeeded in sequencing all coding sequences (CDS) of the *pan*‐BGC of *Pandoraea sputorum* in eight sputum samples (Figure 1b). The high conservation of the *pan* gene locus in clinical isolates motivated us to gain insight into the role of the encoded biosynthetic pathway.

With the aim of bioinformatically predicting the corresponding products of the encoded assembly line, we more closely examined the *pan* locus. Genes *panI*, *panJ*, and *panK* tentatively code for a hexamodular NRPS. Analysis of the six adenylation domains using antiSMASH^[^ ^28^ ^]^ and the PKS/NRPS analysis tool^[^ ^29^ ^]^ indicated substrate specificities for *N^δ^*‐hydroxy‐ornithine (*N^δ^*‐OH‐Orn), aspartic acid (Asp), alanine (Ala), and threonine (Thr) (Tables S3 and S4). Furthermore, the presence of an N‐terminal fatty acyl‐AMP ligase (FAAL) domain suggests that the NRPS is primed with an acyl unit. Overall, the product of the NRPS was predicted to comprise an *N*‐acyl‐*N^δ^*‐OH‐Orn–Asp–Thr–*N^δ^*‐OH‐Orn–Ala–Asp backbone (Figure 1c).

**Figure 2:**
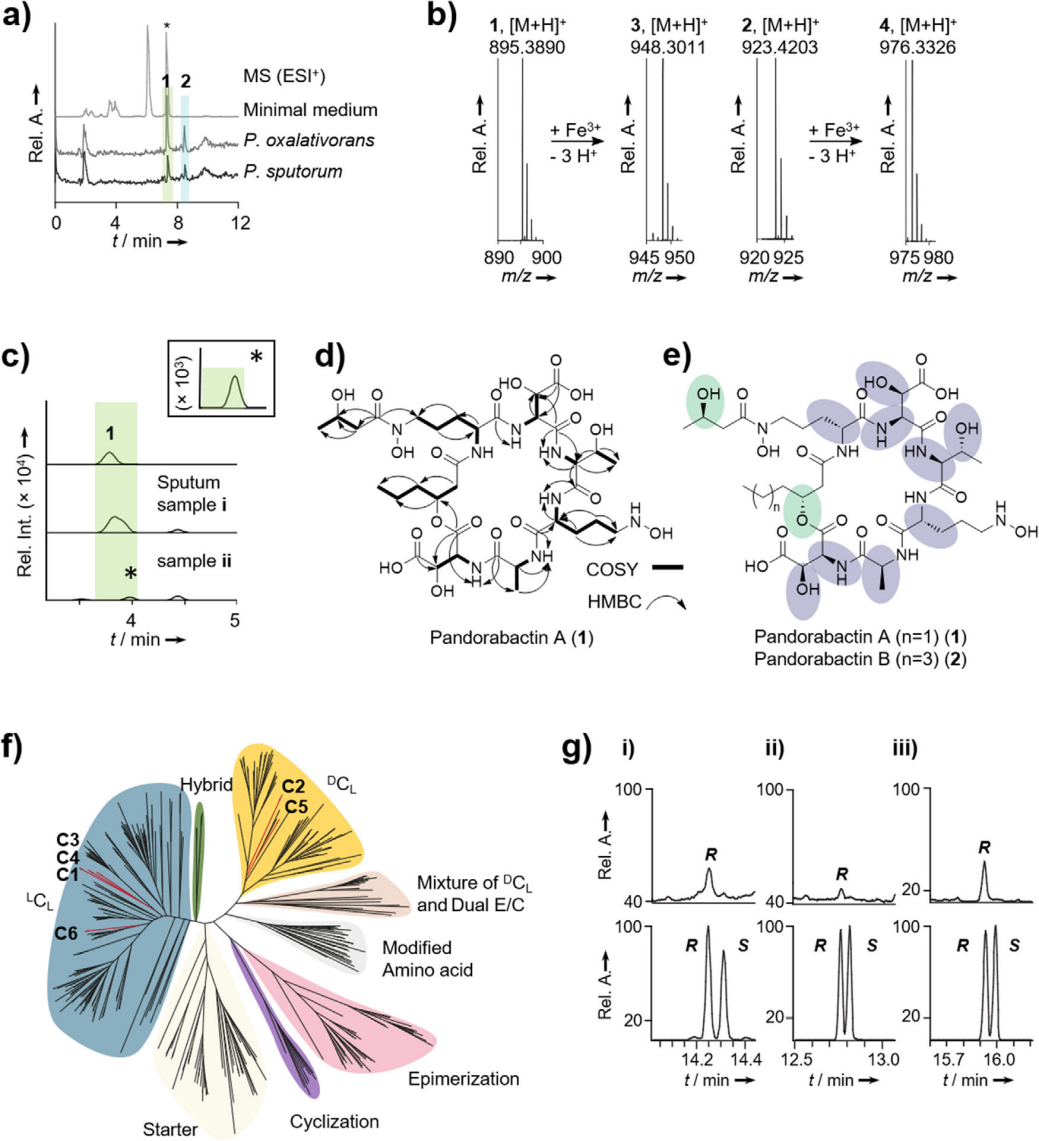
Structure elucidation of pandorabactin A and B. a) HRMS (ESI^+^) profile of extracted minimal medium (light gray) (* signal exhibits an unrelated *m*/*z*), *P. oxalativorans* extract (gray), and *P. sputorum* extract (black). b) Isotope pattern of the metabolites **1** (found *m*/*z* = 895.3890 [M + H]^+^) and **2** (found *m*/*z* = 923.4203 [M + H]^+^) and their corresponding iron complexes **3** and **4**. c) HPLC‐HRMS (ESI^+^) (EIC, extracted ion chromatogram, *m*/*z* 895.3891 ± 0.1 ppm) profiles of pandorabactin A (**1**) as a standard, and extracted sputum samples (i and ii) with possible traces of pandorabactin A (**1**). d) Structure elucidation by NMR analysis showing key ^1^H‐^1^H‐COSY and ^1^H‐^13^C‐HMBC correlations of pandorabactin A (1). e) Stereochemical analysis with the Marfey method (violet) and Mosher method (green) for pandorabactin A (*n* = 1) (**1**) and pandorabactin B (*n* = 3) (**2**). f) Phylogenetic analysis of the C‐domains (red) of *P. sputorum* and *P. oxalativorans*. g) Stereochemical elucidation of i) 3‐OH‐hexanoic acid in pandorabactin A as Mosher methyl ester (upper trace) and derivatized racemic 3‐OH‐hexanoic acid (lower trace), ii) 3‐OH‐butyric acid in pandorabactin A as Mosher methyl ester (upper trace) and derivatized racemic 3‐OH‐butyric acid (lower trace), and iii) 3‐OH‐octanoic acid in pandorabactin B as Mosher methyl ester (upper trace) and derivatized racemic 3‐OH‐octanoic acid (lower trace) by GC‐MS.

Guided by the structural prediction, we conducted the metabolic profiling of two selected strains, the clinical isolate *P. sputorum* DSM 21091 and the environmental isolate *Pandoraea oxalativorans* DSM 23570. We cultivated the strains in minimal medium, extracted the cultures with Amberlite XAD‐16 and subjected the extracts to high‐performance liquid chromatography with diode array detection coupled to high‐resolution mass spectrometry (HPLC‐DAD‐HRMS). We detected two metabolites whose masses are in the range of the predicted product of the *pan*‐NRPS. From the HRMS data (**1**, *m*/*z* 895.3890 [M + H]^+^; **2**, *m*/*z* 923.4203 [M + H]^+^), we deduced the elemental compositions of C_35_H_59_O_19_N_8_ (calc. *m*/*z* 895.3891) and C_37_H_63_O_19_N_8_ (calc. *m*/*z* 923.4204), respectively (Figure 2a). In addition, the MS/MS fragmentation patterns indicated that these compounds are congeners (Figures S1 and S2). Notably, the metabolic profiles also showed a compound **3** with *m*/*z* 948.3011 [M + H]^+^ (C_35_H_56_O_19_N_8_Fe), and a compound **4** with *m*/*z* 976.3326 [M + H]^+^ (C_37_H_60_O_19_N_8_Fe), which correspond to the iron complexes of **1** and **2**, respectively (Figure 2b). Thus, **1** and **2** likely represent *Pandoraea* siderophores, which we named pandorabactin A and B.

To test whether pandorabactins are detectable in the sputum of CF patients, we analyzed *pan*‐positive samples (approx. 250 µL) by HPLC‐HRMS. We observed the expected mass feature *m*/*z* 895.3903 (1.34 ppm) for the major congener at the appropriate retention time in two samples, albeit with only low signal intensity (Figures 2c and S4). We minimized the limit of detection (LOD) = 12 and 15 ng mL^−1^ for pandorabactin A (**1**) and B (**2**), respectively, and combined all samples into one, yet could not achieve adequate signal intensities to rigorously validate the mass features’ identity via isotopic pattern and MS/MS fragmentation. Interestingly, in the course of these analyses, we detected pyochelin and pyocyanin in all *pan*‐positive samples, indicating a co‐infection with *Pseudomonas aeruginosa*.

To obtain sufficient amounts of the compounds **1** and **2** for structural elucidation, we extracted a culture (1 L) of *P. oxalativorans* using Amberlite XAD‐16 and separated the eluate chromatographically. Pure **1** (25.0 mg) and **2** (16.4 mg) were obtained as white powders through preparative reversed‐phase HPLC. We next tested whether **1** and **2** are cyclic or linear peptides. HPLC‐HRMS analysis of the basic hydrolysates revealed mass shifts compared to the parent compounds, *m*/*z* 913.4005 [M + H]^+^ (C_35_H_61_O_20_N_8_) and *m*/*z* = 941.4316 [M + H]^+^ (C_37_H_65_O_20_N_8_), indicating that **1** and **2** were cleaved to give the linear forms. Therefore, we concluded that the native pandorabactins are cyclic peptides with a lactone bond.

We used 1D‐ and 2D‐NMR experiments to determine the architectures of **1** and **2**. Various solvents were tested, of which DMF‐*d*_7_ was found to be the most efficient in producing satisfactory NMR spectral data. Interpretation of the ^1^H‐ and ^13^C‐NMR spectra of pandorabactin A (**1**) in DMF‐*d*_7_ revealed that six amide protons (7.30–9.21 ppm) and ten carbonyl carbons (169.0–173.2 ppm) are peptidic in nature (Tables S6, S7, and Figures S21–S36). The number of carbons in pandorabactin A (**1**) is consistent with the molecular formula deduced by HRMS. Eight spin systems could be detected by ^1^H‐^1^H‐COSY and ^1^H‐^13^C‐HMBC correlations, consisting of six amino acids and two fatty acids (Figure 2d). Among them, Thr and Ala were readily deciphered. The downfield shifts of the β‐carbon signals of both Asp residues (*δ*_C_ = 71.6 ppm for Asp1 and 71.3 ppm for Asp2) indicate that they are substituted with hydroxyl groups. Likewise, downfield shifts of the Orn methylene groups (*δ*_C_ = 47.0 ppm, 47.9 ppm) suggested that the amino groups are hydroxylated (*N^δ^*‐OH‐Orn). ^1^H‐^13^C‐HMBC correlations between the carbonyl carbons and amide protons as *N^δ^*‐OH‐Orn, 3‐OH‐Asp, Thr, *N^δ^*‐OH‐Orn, Ala, and 3‐OH‐Asp confirmed the amino acid sequence of the pandorabactin backbone predicted by bioinformatics and MS/MS fragmentation data of the linearized peptides (Figures S9 and S13).

In accordance with the bioinformatic prediction, compounds **1** and **2** contain N‐terminal acyl groups. NMR data of **1** revealed that a β‐hydroxy‐hexanoyl residue is linked to the amino group of *N^δ^*‐OH‐Orn. Consistent with the mass difference of two methylene groups between pandorabactin A (**1**) and B (**2**), NMR analysis of **2** indicated the presence of the homologous β‐hydroxy‐octanoyl moiety. ^1^H‐^13^C‐HMBC correlations imply that the β‐hydroxy groups of the C6 and C8 side chains are connected to the carbonyl of the second OH‐Asp (*δ*_C_ = 169.0 ppm for pandorabactin A (**1**) and 169.2 ppm for pandorabactin B (**2**)), thus forming a lactone bond. Finally, the ^1^H‐^13^C‐HMBC correlation between the carbonyl carbon at *δ*_C_ = 172.6 ppm and a methylene group (C*_δ_*) of *N^δ^*‐OH‐Orn (*δ*_H_ = 3.72 and 3.63 ppm), and a downfield‐shifted β‐carbon signal (*δ*_C_ = 64.2 ppm) pointed to a 3‐hydroxy‐butyryl residue. According to ^1^H‐^13^C‐HMBC and ^1^H‐^1^H‐NOESY experiments, this acyl group is bound to the N‐terminal *N^δ^*‐OH‐Orn forming a hydroxamate group.

Pandorabactins A (**1**) and B (**2**) expand the group of siderophores with mixed ligands. Each possesses one hydroxamate group harboring a rare 3‐hydroxy‐butyryl residue, like cupriachelin^[^ ^30^ ^]^ and ornibactin C4,^[^ ^31^ ^]^ which differs from the *N^δ^*‐formyl‐ or *N^δ^*‐acetyl‐*N^δ^*‐OH‐Orn moieties typically found in siderophores.^[^ ^32^ ^]^

To determine the absolute configurations of the amino acids, we derivatized the acidic hydrolysate with Marfey's reagent^[^ ^33^ ^]^ and analyzed the 1‐fluoro‐2,4‐dinitrophenyl‐5‐l‐alanine‐amide (l‐FDAA) adducts with HPLC. To identify the absolute configuration of *N^δ^*‐OH‐Orn, we cleaved the hydroxylamine bond using concentrated HI^[^ ^31^, ^34^ ^]^ and compared the resulting Orn with authentic references. For both **1** and **2**, we identified l‐*erythro*‐3‐OH‐Asp, l‐Ala, l‐Thr, and d‐*N^δ^*‐OH‐Orn as amino acid building blocks (Figure 2e and Table S8). To corroborate these findings, we performed a phylogenetic analysis (maximum likelihood)^[^ ^35^ ^]^ of the *pan*‐NRPS C domains. According to the cladogram, all C domains belong to the ^L^C_L_ clade, but the C domains of modules 2 and 5 belong to the ^D^C_L_ clade, which is in line with the positions of the epimerization (E) domains (Figure 2f).

We determined the absolute configuration of the 3‐hydroxy acids by derivatization with Mosher's reagent.^[^ ^36^ ^]^ Both **1** and **2** were independently hydrolyzed, methylated, and treated with (*S*)‐(+)‐MTPA‐Cl. Gas chromatography‐mass spectrometry (GC‐MS) analysis of the resulting conjugates and comparison to authentic references revealed an *R*‐configuration of 3‐hydroxy‐hexanoic acid in **1** (Figure 2g). Likewise, we identified an *R* configuration of 3‐hydroxy‐octanoic acid in **2** (Figure 2g). Identifying the absolute configuration of 3‐hydroxy‐butyric acid, which is present in both **1** and **2**, proved to be cumbersome because the hydrolyzed acid readily racemizes under the reaction conditions. Shortening of the reaction time prevented the isomerization and allowed us to detect (*R*)‐3‐hydroxy‐butyric acid from **1** in trace amounts (Figure 2g).

**Figure 3:**
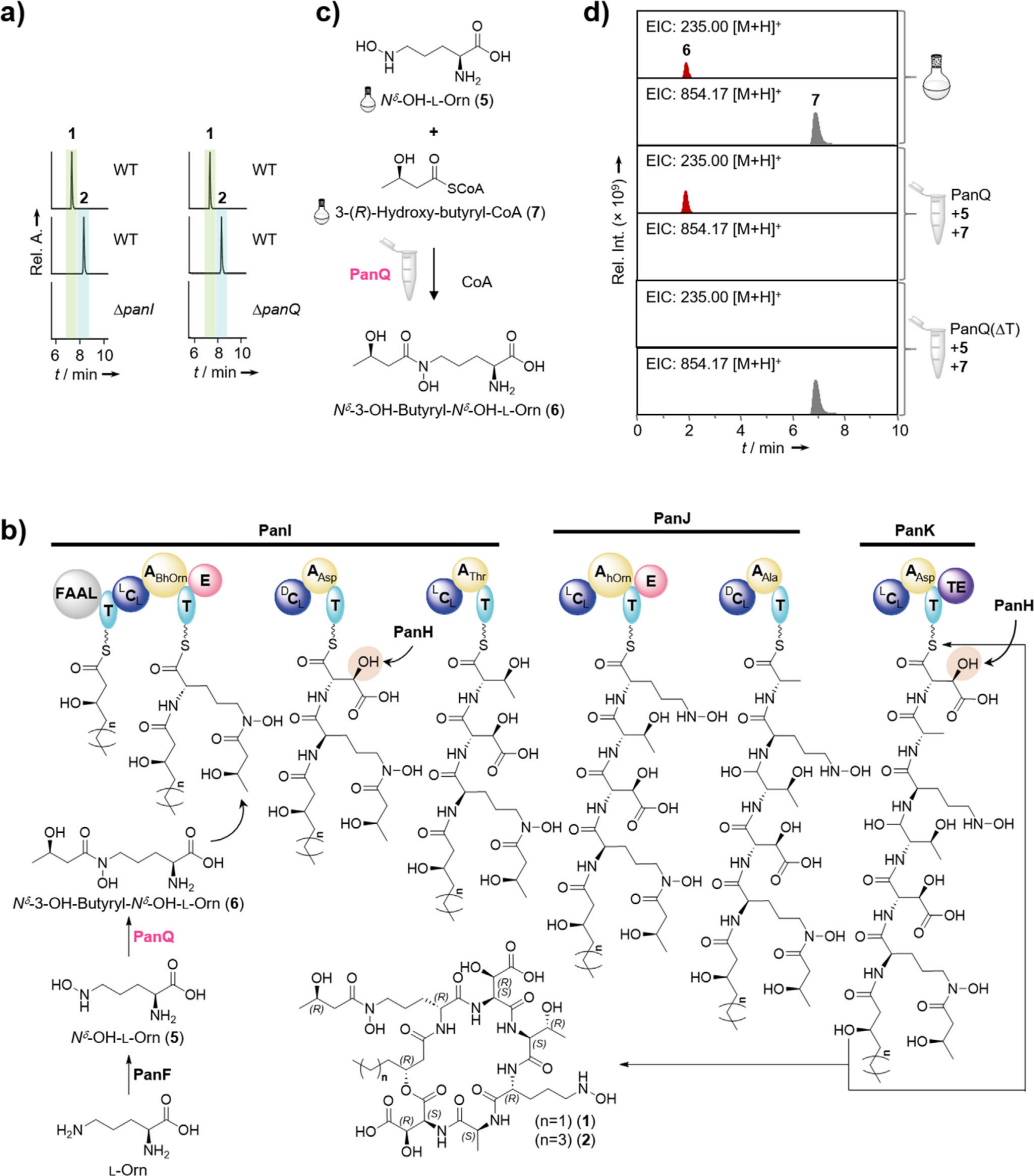
Biosynthesis pathway analysis a) HPLC‐MS (ESI^+^) (EIC, extracted ion chromatogram) profiles of extracts of *P. oxalativorans* wild type (WT), *P. oxalativorans* Δ*panI* and *P. oxalativorans* Δ*panQ* cultures; extracted ion chromatograms of the [M + H]^+^ ionic species for pandorabactin A (**1**) and B (**2**). b) Proposed biosynthesis to pandorabactin A (**1**) and B (**2**), where PanF generates *N^δ^*‐OH‐l‐Orn (**5**) and PanQ further modifies it to *N^δ^*‐3‐OH‐butyryl‐*N^δ^*‐OH‐l‐Orn (**6**), BhOrn, *N^δ^*‐3‐OH‐butyryl‐*N^δ^*‐OH‐l‐Orn; hOrn, *N^δ^*‐OH‐l‐Orn. c) Schematic representation of the in vitro reconstitution of PanQ function, which transfers the *N*‐((*R*)‐3‐hydroxy‐butyryl) group of CoA thioester to *N^δ^* of *N^δ^*‐hydroxy‐l‐ornithine (**5**) with the synthetic substrates *N^δ^*‐OH‐l‐Orn (**5**) and 3‐(*R*)‐hydroxy‐butyryl‐CoA (**7**). d) HPLC‐MS (ESI^+^) (EIC, extracted ion chromatogram) profiles of in vitro activity assay of PanQ, which transfers *N*‐((*R*)‐3‐OH‐butyryl) group of CoA thioester to *N^δ^* of *N^δ^*‐OH‐l‐Orn (**5**), with the standards *N^δ^*‐3‐OH‐butyryl‐*N^δ^*‐OH‐l‐Orn (**6**) and 3‐(*R*)‐hydroxy‐butyryl‐CoA (**7**).

To determine if the putative siderophores **1** and **2** originate from the *pan* locus, we aimed at a targeted gene inactivation approach. Since both strains produce identical metabolites (**1**–**4**), we turned to the risk group 1 strain *P. oxalativorans* for genetic manipulation and large‐scale cultivation. Specifically, we replaced the *panI* gene with a chloramphenicol resistance cassette by homologous double crossover, following a method established for related *Burkholderia* species.^[^ ^34^, ^37^ ^]^ We confirmed the successful construction of *P. oxalativorans* Δ*panI* with PCR (Figure S15). Comparison of the metabolic profiles of *P. oxalativorans* Δ*panI* and the wild type (wt) revealed that **1**–**4** are only produced when the *pan*‐NRPS is intact (Figure 3a). Therefore, we concluded that the *pan*‐BGC is responsible for pandorabactin biosynthesis.

The structures of **1** and **2** align with a plausible biosynthetic model gleaned from deduced *pan*‐gene functions (Figure 3b). After loading of the C6 or C8 hydroxy acids by the FAAL domain, the hexapeptide backbone is assembled by the NRPS in a canonical fashion. Pandorabactin assembly would be concluded with the thioesterase‐mediated release and cyclization of the hexapeptide, initiated by a nucleophilic attack of β‐hydroxy groups of the N‐terminal fatty acids onto the thioester.

The l‐*erythro*‐3‐OH‐Asp moiety of **1** and **2** is likely formed by PanH, which is related to dioxygenases that have been shown to hydroxylate l‐Asp bound to the peptidyl carrier protein.^[^ ^30^, ^38^ ^]^ A BLASTp^[^ ^39^ ^]^ analysis indicates that PanF is related to similar oxygenases that *N*‐hydroxylate Lys and Orn prior to their incorporation into the peptide backbone.^[^ ^40^, ^41^, ^42^ ^]^

To investigate the timing of the acylation of *N^δ^*‐OH‐Orn (**5**), that is, before or after loading onto the NRPS, we performed both in vivo chemical complementation and in vitro enzymatic reconstitution experiments. Since the PKS/NRPS analysis tool^[^ ^29^ ^]^ predicts an A domain substrate specificity for *N^δ^*‐OH‐Orn, hydroxylation of l‐Orn likely takes place before its incorporation into the peptide backbone. Unfortunately, all attempts to obtain soluble protein for an A domain assay were futile.

Thus, we next attempted to gain insight through functional analyses of the modifying enzymes. The deduced gene product of *panQ* is the best candidate for an *N*‐acyltransferase required to append the 3‐hydroxy‐butyryl moiety to the first *N^δ^*‐OH‐l‐Orn (**5**) to generate *N^δ^*‐3‐OH‐butyryl‐*N^δ^*‐OH‐l‐Orn (**6**), which is found in the final product. A targeted inactivation of *panQ* led to abrogation of pandorabactin biosynthesis, confirming the crucial role of PanQ in the assembly.

Initially, we attempted to chemically complement the mutant *P. oxalativorans* Δ*panQ* to test whether **6** is loaded onto the NRPS. Therefore, we synthesized based on a previously reported procedure^[^ ^43^ ^]^ (Scheme S1, Figures S42, and S43) and supplemented the culture of the Δ*panQ* mutant. However, pandorabactins could not be detected in the extract by HPLC‐HRMS analysis. Plausible explanations are that a) **6** is not loaded onto the NRPS or b) **6** is not taken up by the cells. Notably, the synthetic substrate was identified in the supernatant extracts (Figure S16). Moreover, we were able to detect **6** in *P. oxalativorans* wild type and in the Δ*panI* mutant, but not in the Δ*panQ* mutant (Figure S16). This result suggests that **6** is formed as a biosynthetic building block.

To corroborate this, we cloned the *panQ* gene into the pET28 vector for heterologous production in *Escherichia coli* BL21 (DE3) and purified His_6_‐tagged PanQ by Ni‐NTA column (Figure S17). We reconstituted the biotransformation in vitro with His_6_‐PanQ, synthetic **5**, and 3‐(*R*)‐hydroxy‐butyryl‐CoA (**7**) in Tris HCl (pH 7.5). Detection of **6** by HPLC‐HRMS analysis indicated the successful *N^δ^*‐OH‐Orn *N^δ^*‐acyltransfer in the enzyme assay (Figures 3c,d and S17). These results confirm that PanQ is a CoA‐dependent *N^δ^*‐3‐(*R*)‐hydroxy‐butyryl‐*N^δ^*‐OH‐Orn‐transferase and suggest that **6** is formed before it is loaded onto PanI (Figures 3b and S17). This scheme is in agreement with the model for vicibactin biosynthesis, where **6** is biosynthesized before loading onto the NRPS.^[^ ^43^ ^]^

**Figure 4:**
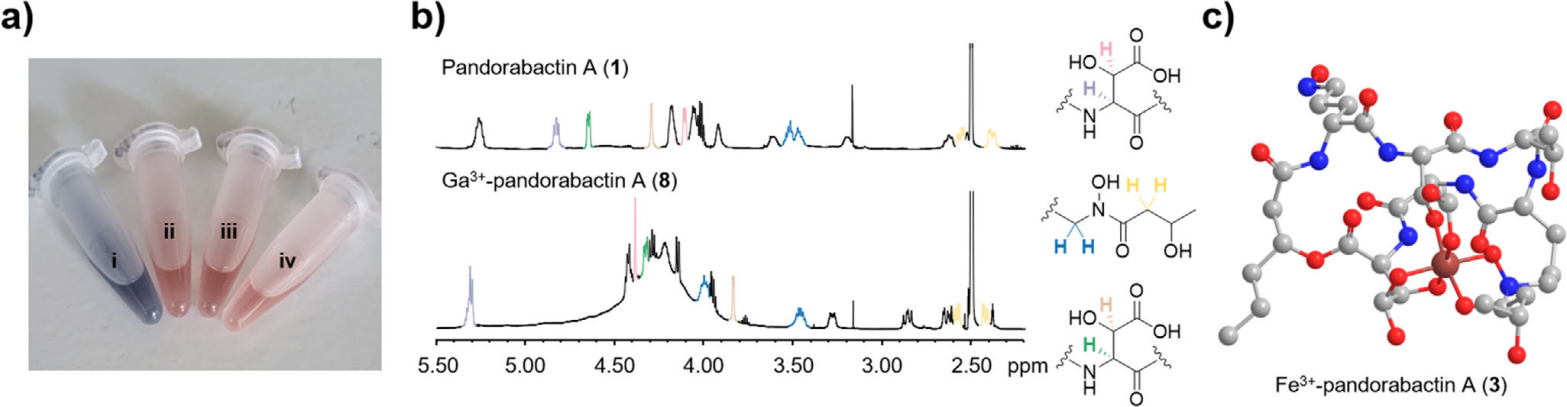
Iron‐chelating property of pandorabactins. a) Colorimetric assay using CAS reagent with water (i), EDTA (ii), pandorabactins A (iii) and B (iv). b) Identification of iron‐binding sites by ^1^H‐NMR spectral comparison between pandorabactin A (**1**) and the Ga^3+^‐pandorabactin A (**8**). c) 3D model of the Fe^3+^‐pandorabactin A (**3**) complex calculated with Perkin Elmer Chem3D Pro (hydrogen atoms not shown, carbon (gray), nitrogen (blue), oxygen (red), and iron (brown)).

To verify the anticipated chelating activity of the pandorabactins, we performed a chrome azurol S (CAS) assay (Figure 4a).^[^ ^44^ ^]^ Both pandorabactin A (**1**) and B (**2**) induce the characteristic color change from blue to red, indicating iron complexation. To identify the Fe^3+^ binding sites in **1** and **2** by NMR, we prepared the corresponding gallium complexes **8** and **9** and purified them by preparative HPLC (Tables S12, S13 and Figures S45–S60). By comparing the chemical shifts in the proton NMR spectra, we identified the two 3‐OH‐Asp moieties and the hydroxamate group (*N^δ^*‐3‐OH‐butyryl‐*N^δ^*‐OH‐d‐Orn) as binding sites (Figure 4b). This enabled us to create a 3D model for the structure of Fe^3+^‐pandorabactin A (**3**) as a stable octahedral complex (Figure 4c).

**Figure 5:**
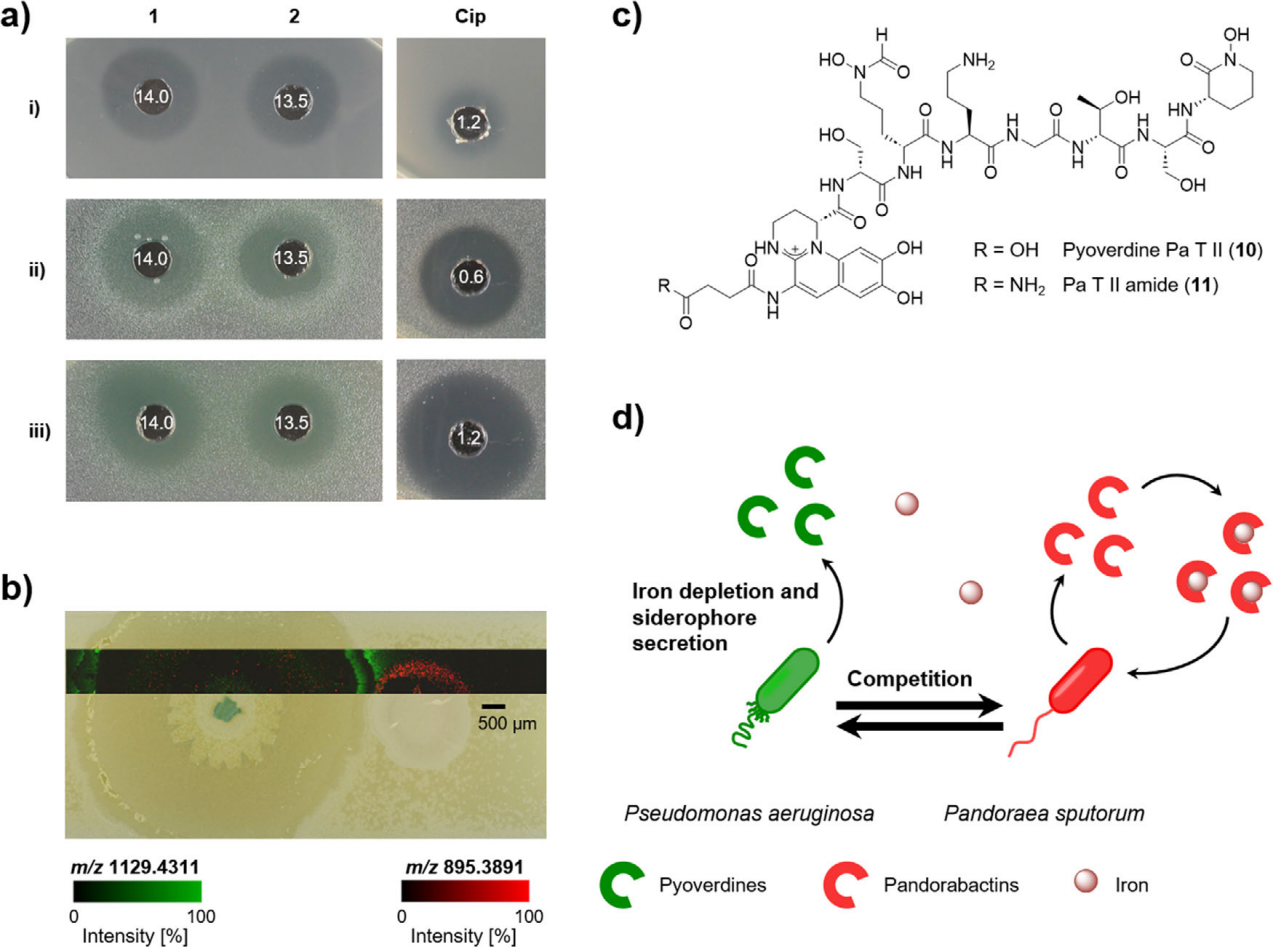
Pandorabactins inhibit bacterial growth and trigger the production of pyoverdines. a) Examples of the inhibition zones observed in lawns of o) *Mycobacterium vaccae*, ii) *Pseudomonas aeruginosa* SG 137, and iii) *Pseudomonas aeruginosa* K799/61 strains in an agar diffusion assay using a concentration of 1 mg mL^−1^ of pandorabactin A (**1**) and B (**2**), and ciprofloxacin as a positive control with a concentration of 5 µg mL^−1^, along with minimum inhibitory concentration (MIC) values given in µm . b) Visualization of the siderophores pandorabactin A (**1**) (*m*/*z* 895.3891 ± 0.017 [M + H]^+^) and pyoverdine **11** (*m*/*z* 1129.4311 ± 0.017 [M + K]^+^) in the coculture of *P. sputorum* (red) with *P. aeruginosa* (green) by MALDI‐MSI (root mean square normalization). c) Structures of pyoverdines **10** and **11**. d) In the coculture of *P. sputorum* (red) and *P. aeruginosa* (green), the production of pyoverdines is stimulated by the release of pandorabactins, resulting in a competitive interaction.

In addition to playing a role in sequestering iron for the producer, siderophores may have additional biological roles, including the inhibition of competing bacteria.^[^ ^23^, ^45^, ^46^, ^47^ ^]^ To determine potential antimicrobial activities, we subjected pandorabactin A (**1**) and B (**2**) to agar diffusion assays with a set of representative test strains. We measured the minimum inhibitory concentration (MIC) of the metabolites **1** and **2** for a subset of the strains that proved to be most susceptible in the agar diffusion assay (Figure 5a, Tables S14, and S16). Both pandorabactins show moderate antibacterial activities against *Mycobacterium vaccae* and *Pseudomonas aeruginosa* with MICs of 14.0 and 13.5 µm, respectively.

In general, secreted siderophores can inhibit the growth of competing bacteria through two possible mechanisms: acting as an antibiotic or scavenging and depleting Fe(III) that is essential for growth.^[^ ^19^, ^48^, ^49^, ^50^ ^]^ We observed that the antimicrobial activity was not present in the assays when we tested the iron complexes **3** and **4** of both siderophores, suggesting that iron depletion may be the mechanism at play (Table S15). Furthermore, when conducting the agar diffusion assays, we observed a color change in *P. aeruginosa* strains to yellow/green in response to the iron scavenging of pandorabactins. Given that the *P. aeruginosa* siderophores pyochelin and pyoverdine are yellow to yellowish green,^[^ ^51^, ^52^ ^]^ it is likely that *P. aeruginosa* strains produce their own siderophores to counteract the iron deficiency caused by pandorabactins.

The impact of a specialized *Pandoraea* metabolite on the growth and metabolism of *P. aeruginosa* is intriguing, as both *Pandoraea* and *Pseudomonas* species colonize the lungs of CF patients.^[^ ^53^, ^54^, ^55^ ^]^ Specifically, *P. aeruginosa* as well as pyoverdines were detected in CF patients.^[^ ^56^, ^57^ ^]^ Notably, the *pan*‐BGC is confirmed by our metagenome data of the lung microbiota of CF patients and MS analysis of *pan*‐positive sputum samples (see above).

To mimic a potentially disease‐relevant microbial interaction, we cocultured the lung isolates *P. aeruginosa* with *P. sputorum* on an iron‐deficient minimal agar. We analyzed the spatial distribution of secreted metabolites directly from the coculture of both pathogens by matrix‐assisted laser desorption/ionization mass spectrometry imaging (MALDI‐MSI). We observed the mass features *m*/*z* 895.39 and 923.42, putatively assigned to the [M + H]^+^ of pandorabactin A (**1**) and B (**2**), respectively, in the area of *P. sputorum* growth and the surrounding area (Figure 5a). Additionally, we observed *m*/*z* 1092.46 and 1091.48, which we putatively assigned to the [M + H]^+^ of pyoverdine **10** and **11**, respectively, with the highest intensities detected in the interaction zone of the coculture (Figure 5b,c). The putative assignment of structural identity is based on the accurate mass and isotopic pattern acquired by HPLC‐HRMS, which were connected to the MALDI‐MSI results by sampling dried droplets of the extracts under MSI conditions. Further corroboration of the structural identity is the potassium and sodium adduct ions present in the MALDI analyses.

As controls, we cocultured *P. aeruginosa* with the pandorabactin‐negative mutant, *P. oxalativorans* Δ*panI*, as well as each strain individually. We extracted the interaction area between the cocultures and the area next to the monocultures, then analyzed the extracts by HPLC‐HRMS. In extracts of *P. aeruginosa* monocultures, we detected pyochelin and pyocyanin but only traces of pyoverdine **10**. On average, the extracts of *P. aeruginosa* with *P. oxalativorans* Δ*panI* exhibited three times higher amounts of pyoverdine **10** than the *P. aeruginosa* monoculture extracts. The extracts from the coculture with *P. sputorum* exhibited an average of nine times more pyoverdine **10**, indicating a strong, specific response to pandorabactins (Table S17). We envision that the production of pyoverdines is stimulated by the release of pandorabactins, leading to a competitive interaction (Figure 5d).

**Figure 6:**
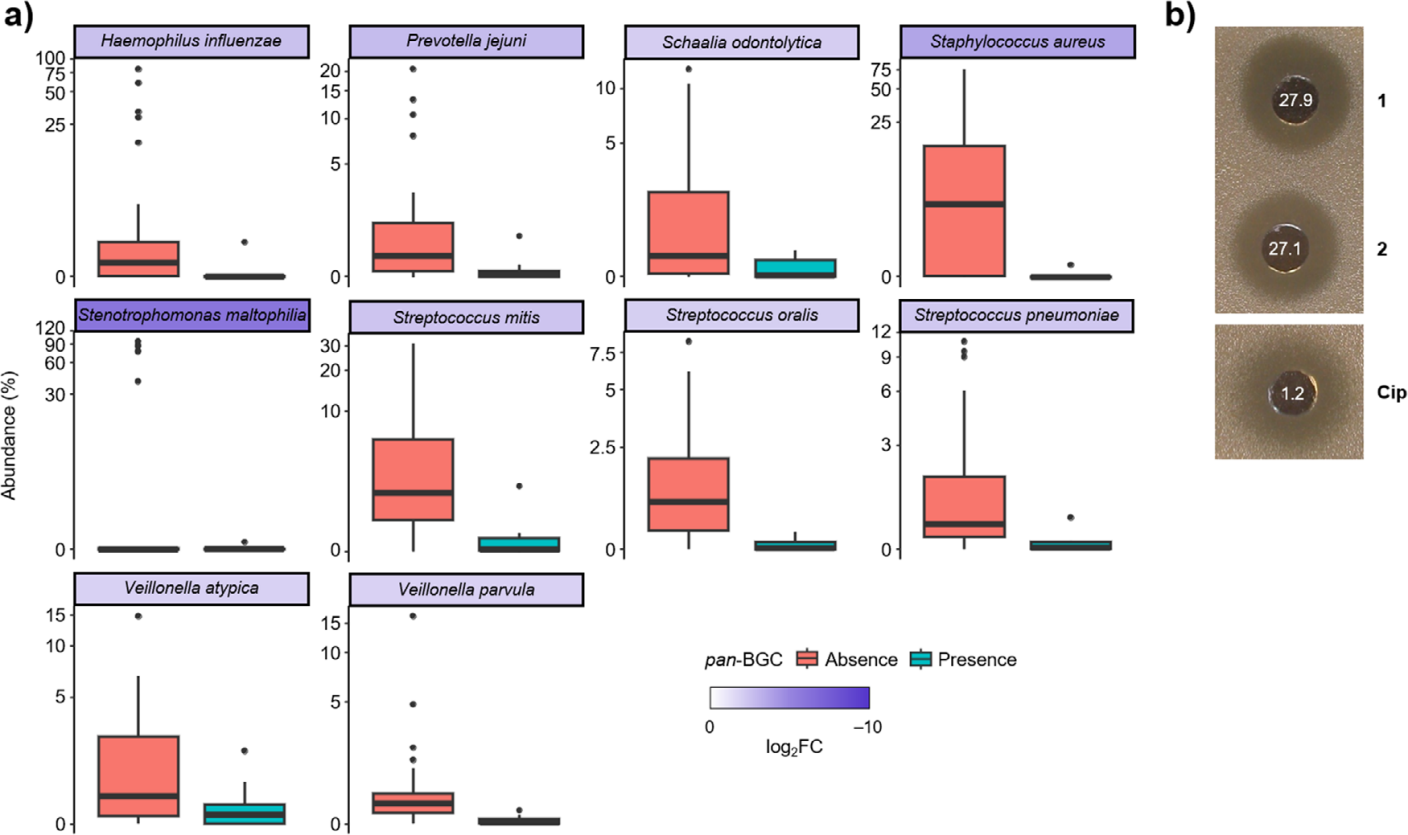
Metagenomic analysis and inhibition of different species. a) Relative abundance of species detected in sputum samples from CF patients (*n* = 40) that differ significantly in abundance when *pan* genes are absent (orange column (left), *n* = 32) or present (blue column (right), *n* = 8). The strip color represents the log2‐normalized ratio of mean genus abundance in the “presence” group relative to the “absence” group. Box‐plot elements: center line: median, lower/upper bound: 25^th^/75^th^ percentile, whiskers: minimum and maximum values within 1.5 × interquartile range (IQR), outliers: points outside ± 1.5 × IQR. b) Inhibition zone observed in the lawn of *Stenotrophomonas maltophilia* strain in an agar diffusion assay using a concentration of 1 mg mL^−1^ of pandorabactin A (**1**) and B (**2**), and ciprofloxacin as a positive control with a concentration of 5 µg mL^−1^, along with MIC values given in µm.

To investigate the potential impact of pandorabactin production on the composition of the diseased lung microbiota, we compared the microbiome of samples with and without the *Pandoraea pan‐*BGC. In the presence of this gene locus, the abundance of 10 bacterial species was significantly altered (*p* < 0.05, DESeq2), with the most pronounced change observed for *Stenotrophomonas maltophilia* (*p* = 0.013, FDR = 0.05, log_2_FC = −8.46 (Figure 6a and Table S18). Notably, *S. maltophilia* is known as a multidrug‐resistant pathogen that primarily causes infections of the respiratory tract but can also infect other organs.^[^ ^58^ ^]^ The abundance of *Streptococcus mitis*, an oral commensal that has also been implicated in infective endocarditis,^[^ ^59^, ^60^, ^61^ ^]^ was also significantly decreased (*p* = 0.001, FDR = 0.013, log_2_FC = −3.62) (Figure 6a and Table S18). The pronounced correlation between the presence of the *pan*‐BGC and the reduced abundance of these species suggests that pandorabactins are produced in vivo and effectively sequester iron from the competitive microbiome environment.

To evaluate the immediate effect of pandorabactins on the strains with the most reduced abundance in the presence of the *pan*‐BGC, we performed agar diffusion assays with *S. maltophilia* DSM 50170 and *S. mitis* DSM 12643. Pandorabactin A (**1**) and B (**2**) were found to inhibit *S. maltophilia* with MIC values of 27.9 and 27.1 µm (Figure 6b and Table S16). In contrast, the iron complexes **3** and **4** showed no growth inhibition, which is in line with the mode of action by iron depletion (Table S15). Interestingly, pandorabactins showed no direct effect on *S. mitis*. This could be explained by strain‐dependent variations in the susceptibility or by indirect effects through the microbiota composition. Nonetheless, the marked inhibition of the multidrug‐resistant *S. maltophilia* strain indicates that pandorabactin A (**1**) and B (**2**) have an immediate impact on the co‐colonizers of the CF lung. As such, *Pandoraea* species that produce pandorabactins seem to gain an advantage in the competitive lung microbiota.

This finding complements other studies showing that iron availability plays an important role in complex microbial interactions and determines the success of individual species within a niche. Important examples of siderophore‐mediated interactions that shape microbial composition are known in the plant rhizosphere,^[^ ^62^ ^]^ the (inflamed) human gut,^[^ ^63^, ^64^, ^65^ ^]^ and the nasal microbiota.^[^ ^66^, ^67^ ^]^ We now provide the first indication that *Pandoraea* siderophores could play a role in the diseased lung microbiota.

## Conclusion

*Pandoraea* species are generally regarded as emerging pathogens that are resistant to antibiotic treatment. Despite their growing clinical role, these Gram‐negatives are still very little explored in terms of niche factors and virulence, which may promote host colonization and infection. Remarkably, neither siderophores nor other specialized metabolites from *Pandoraea* strains have been structurally elucidated. In this study, we identify a conserved gene cluster that is distributed across representatives of both environmental and clinical isolates and is abundant in the metagenomes of CF lung samples. Mutational analyses showed that the presence of an intact gene cluster correlates with siderophore production. Using a combination of in silico and chemical analyses, we fully elucidated the structures and iron‐chelating potential of pandorabactins. They are the first characterized natural products from this important bacterial genus and add to the knowledge on siderophores of human pathogens.^[^ ^68^ ^]^

Our findings suggest that the capacity to produce pandorabactins provides an important survival advantage in a competitive environment. In the lung, where bioavailable iron is scarce, the acquisition of this essential metal is crucial for the adaptation of pathogens in the host.^[^ ^69^ ^]^ As such, siderophores play an important role as niche factors in colonization and chronic infections such as cystic fibrosis.^[^ ^20^ ^]^

The ability of pandorabactins to inhibit the growth of bacterial pathogens such as mycobacteria, pseudomonads, and stenotrophomonads co‐occurring in CF lungs is particularly intriguing. MALDI‐MSI, metabolic profiling, and bioassays suggest that the antibacterial activity of pandorabactins results from iron depletion of the competitors. The correlation analysis of the CF microbiomes in conjunction with bioassays provides the first indication that pandorabactins modulate the lung microbiota of CF patients. This is an important addition to analogous findings in other niches, further highlighting the substantial role of iron acquisition in shaping microbial communities.

Taken together, our findings suggest that producers of the secreted pandorabactins may deplete or even outcompete other bacteria sharing the same habitat, which could, at least partially, explain the success of emerging pathogenic *Pandoraea* species. The discovery of pandorabactins and elucidation of their functions may not only serve as a starting point for siderophore‐based drug development but could also inspire therapeutic interventions to cure severe and chronic lung infections.

## Author Contributions

E.H., C.H., and S.P.N. designed the research work. E.H. K.I., and B.B. performed experiments. X.C. performed bioinformatic analysis. E.H., K.I., and K.S. analyzed data. B.C. provided sputum samples. C.H. and G.P. supervised the research work.

## Conflict of Interests

The authors declare no conflict of interest.

## Supporting information



Supporting Information

## Data Availability

The data that support the findings of this study are available in the Supporting Information of this article.

## References

[1] T. Coenye , E. Falsen , B. Hoste , M. Ohlén , J. Goris , J. Govan , M. Gillis , P. Vandamme , Int. J. Syst. Evol. Microbiol. 2000, 50, 887–899. 10758901 10.1099/00207713-50-2-887

[2] T. Coenye , L. Liu , P. Vandamme , J. J. LiPuma , J. Clin. Microbiol. 2001, 39, 4452–4455. 11724860 10.1128/JCM.39.12.4452-4455.2001 PMC88564

[3] N. Sahin , A. Tani , R. Kotan , I. Sedláček , K. Kimbara , A. U. Tamer , Int. J. Syst. Evol. Microbiol. 2011, 61, 2247–2253. 20952546 10.1099/ijs.0.026138-0

[4] M. Puges , S. Debelleix , M. Fayon , F. Megraud , P. Lehours , Pediatr. Infect. Dis. J. 2015, 34, 1135–1137. 26176630 10.1097/INF.0000000000000843

[5] M. I. Daneshvar , D. G. Hollis , A. G. Steigerwalt , A. M. Whitney , L. Spangler , M. P. Douglas , J. G. Jordan , J. P. MacGregor , B. C. Hill , F. C. Tenover , D. J. Brenner , a. R. S. Weyant , J. Clin. Microbiol. 2001, 39, 1819–1826. 11325997 10.1128/JCM.39.5.1819-1826.2001 PMC88032

[6] L. N. Johnson , J. Y. Han , S. M. Moskowitz , J. L. Burns , X. Qin , J. A. Englund , Pediatr. Infect. Dis. J. 2004, 23, 881–882. 15361734 10.1097/01.inf.0000136857.74561.3c

[7] R. L. Gibson , J. L. Burns , B. W. Ramsey , Am. J. Respir. Crit. Care Med. 2003, 168, 918–951. 14555458 10.1164/rccm.200304-505SO

[8] H. Heijerman , J. Cyst. Fibros. 2005, 4, 3–5. 15970469 10.1016/j.jcf.2005.05.005

[9] F. Harrison , Microbiology 2007, 153, 917–923. 17379702 10.1099/mic.0.2006/004077-0

[10] E. Caraher , J. Collins , G. Herbert , P. G. Murphy , C. G. Gallagher , M. J. Crowe , M. Callaghan , S. McClean , J. Med. Microbiol. 2008, 57, 15–20. 18065662 10.1099/jmm.0.47544-0

[11] C. Bitossi , M. Fracella , A. Viscido , F. Diaco , C. Pecoraro , P. Troiani , G. Cimino , G. d'Ettorre , M. Trancassini , A. Pierangeli , G. Antonelli , C. Scagnolari , J. Immunoassay Immunochem. 2025, 46, 201–206. 39935049 10.1080/15321819.2025.2462807

[12] M. E. Stryjewski , J. J. LiPuma , R. H. Messier, Jr. , L. B. Reller , B. D. Alexander , J. Clin. Microbiol. 2003, 41, 2255–2257. 12734295 10.1128/JCM.41.5.2255-2257.2003 PMC154699

[13] T. Kruis , P. Menzel , R. Schwarzer , S. Wiesener , F. Schoenrath , F. Klefisch , M. Stegemann , F. Pfafflin , Emerg. Infect. Dis. 2023, 29, 2229–2237. 37877517 10.3201/eid2911.230493 PMC10617358

[14] C. Lin , N. Luo , Q. Xu , J. Zhang , M. Cai , G. Zheng , P. Yang , BMC Infect. Dis. 2019, 19, 869. 31640582 10.1186/s12879-019-4420-6 PMC6805617

[15] X. Xiao , H. Tian , X. Cheng , G. Li , J. Zhou , Z. Peng , Y. Li , Infect. Drug Resist. 2019, 12, 3359–3364. 31695454 10.2147/IDR.S227643 PMC6821047

[16] Z. Ma , X. Zou , J. Lin , C. Zhang , S. Xiao , Infect. Drug Resist. 2022, 15, 7043–7052. 36483145 10.2147/IDR.S388520 PMC9725920

[17] V. I. Holden , M. A. Bachman , Metallomics 2015, 7, 986–995. 25745886 10.1039/c4mt00333k

[18] A. Khan , P. Singh , A. Srivastava , Microbiol. Res. 2018, 212–213, 103–111. 10.1016/j.micres.2017.10.012 29103733

[19] J. Kramer , O. Ozkaya , R. Kummerli , Nat. Rev. Microbiol. 2020, 18, 152–163. 31748738 10.1038/s41579-019-0284-4 PMC7116523

[20] J. Tyrrell , M. Callaghan , Microbiology 2016, 162, 191–205. 26643057 10.1099/mic.0.000220 PMC4772740

[21] A. L. Lamb , Biochim. Biophys. Acta 2015, 1854, 1054–1070. 25970810 10.1016/j.bbapap.2015.05.001 PMC4457648

[22] F. Imperi , F. Massai , M. Facchini , E. Frangipani , D. Visaggio , L. Leoni , A. Bragonzi , P. Visca , Proc. Natl. Acad. Sci. USA 2013, 110, 7458–7463. 23569238 10.1073/pnas.1222706110 PMC3645532

[23] R. C. Hider , X. Kong , Nat. Prod. Rep. 2010, 27, 637–657. 20376388 10.1039/b906679a

[24] Y. Shinozaki , H. Kitamoto , Y. Sameshima‐Yamashita , A. Kinoshita , T. Nakajima‐Kambe , J. Gen. Appl. Microbiol. 2019, 65, 273–276. 31019144 10.2323/jgam.2018.12.001

[25] N. Oberg , R. Zallot , J. A. Gerlt , J. Mol. Biol. 2023, 435, 168018. 37356897 10.1016/j.jmb.2023.168018 PMC10291204

[26] C. Peeters , E. De Canck , M. Cnockaert , E. De Brandt , C. Snauwaert , B. Verheyde , E. Depoorter , T. Spilker , J. J. LiPuma , P. Vandamme , Front. Microbiol. 2019, 10, 2556. 31781066 10.3389/fmicb.2019.02556 PMC6851202

[27] M. H. Mirhakkak , X. Chen , Y. Ni , T. Heinekamp , T. Sae‐Ong , L. L. Xu , O. Kurzai , A. E. Barber , A. A. Brakhage , S. Boutin , S. Schauble , G. Panagiotou , Nat. Commun. 2023, 14, 4369. 37474497 10.1038/s41467-023-39982-5 PMC10359302

[28] K. Blin , S. Shaw , H. E. Augustijn , Z. L. Reitz , F. Biermann , M. Alanjary , A. Fetter , B. R. Terlouw , W. W. Metcalf , E. J. N. Helfrich , G. P. van Wezel , M. H. Medema , T. Weber , Nucleic Acids Res. 2023, 51, W46–W50. 37140036 10.1093/nar/gkad344 PMC10320115

[29] B. O. Bachmann , J. Ravel , Methods Enzymol. 2009, 458, 181–217. 19374984 10.1016/S0076-6879(09)04808-3

[30] M. F. Kreutzer , H. Kage , M. Nett , J. Am. Chem. Soc. 2012, 134, 5415–5422. 22381697 10.1021/ja300620z

[31] H. Stephan , S. Freund , W. Beck , G. Jung , J. M. Meyer , G. Winkelmann , BioMetals 1993, 6, 93–100. 7689374 10.1007/BF00140109

[32] D. Al Shaer , O. Al Musaimi , B. G. de la Torre , F. Albericio , Eur. J. Med. Chem. 2020, 208, 112791. 32947228 10.1016/j.ejmech.2020.112791

[33] K. Fujii , Y. Ikai , T. Mayumi , H. Oka , M. Suzuki , K.‐i. Harada , Anal. Chem. 1997, 69, 3346–3352.

[34] J. Franke , K. Ishida , M. Ishida‐Ito , C. Hertweck , Angew. Chem. Int. Ed. 2013, 52, 8271–8275; 10.1002/anie.201303196 23821334

[35] L. T. Nguyen , H. A. Schmidt , A. von Haeseler , B. Q. Minh , Mol. Biol. Evol. 2015, 32, 268–274. 25371430 10.1093/molbev/msu300 PMC4271533

[36] R. Jenske , W. Vetter , J. Agric. Food Chem. 2008, 56, 11578–11583. 19090708 10.1021/jf802772a

[37] F. Trottmann , K. Ishida , J. Franke , A. Stanisic , M. Ishida‐Ito , H. Kries , G. Pohnert , C. Hertweck , Angew. Chem. Int. Ed. 2020, 59, 13511–13515; 10.1002/anie.202003958 PMC7496086 32314848

[38] H. Chen , L. Zhong , H. Zhou , T. Sun , G. Zhong , Q. Tu , Y. Zhuang , X. Bai , X. Wang , J. Xu , L. Xia , Y. Shen , Y. Zhang , X. Bian , Angew. Chem. Int. Ed. 2022, 61, e202203591; 10.1002/anie.202203591 35689369

[39] S. F. Altschul , W. Gish , W. Miller , E. W. Myers , D. J. Lipman , J. Mol. Biol. 1990, 215, 403–410. 2231712 10.1016/S0022-2836(05)80360-2

[40] I. J. Schalk , L. Guillon , Environ. Microbiol. 2013, 15, 1661–1673. 23126435 10.1111/1462-2920.12013

[41] O. Yamada , S. Na Nan , T. Akao , M. Tominaga , H. Watanabe , T. Satoh , H. Enei , O. Akita , J. Biosci. Bioeng. 2003, 95, 82–88. 16233371 10.1016/S1389-1723(03)80153-6

[42] M. D. McMahon , J. S. Rush , M. G. Thomas , J. Bacteriol. 2012, 194, 2809–2818. 22447909 10.1128/JB.00088-12 PMC3370630

[43] J. R. Heemstra Jr. , C. T. Walsh , E. S. Sattely , J. Am. Chem. Soc. 2009, 131, 15317–15329. 19778043 10.1021/ja9056008 PMC2783850

[44] B. Schwyn , J. B. Neilands , Anal. Biochem. 1987, 160, 47–56. 2952030 10.1016/0003-2697(87)90612-9

[45] T. C. Johnstone , E. M. Nolan , Dalton Trans. 2015, 44, 6320–6339. 25764171 10.1039/c4dt03559c PMC4375017

[46] B. R. Wilson , A. R. Bogdan , M. Miyazawa , K. Hashimoto , Y. Tsuji , Trends Mol. Med. 2016, 22, 1077–1090. 27825668 10.1016/j.molmed.2016.10.005 PMC5135587

[47] M. Ribeiro , M. Simões , Environ. Chem. Lett. 2019, 17, 1485–1494.

[48] M. E. Hibbing , C. Fuqua , M. R. Parsek , S. B. Peterson , Nat. Rev. Microbiol. 2010, 8, 15–25. 19946288 10.1038/nrmicro2259 PMC2879262

[49] N. Tejman‐Yarden , A. Robinson , Y. Davidov , A. Shulman , A. Varvak , F. Reyes , G. Rahav , I. Nissan , Front. Microbiol. 2019, 10, 2377. 31681234 10.3389/fmicb.2019.02377 PMC6808179

[50] K. S. Ong , Y. L. Cheow , S. M. Lee , J. Adv. Res. 2017, 8, 393–398. 28580180 10.1016/j.jare.2017.05.007 PMC5447373

[51] J. M. Meyer , Arch. Microbiol. 2000, 174, 135–142. 11041343 10.1007/s002030000188

[52] C. D. Cox , K. L. Rinehart Jr. , M. L. Moore , J. C. Cook Jr. , Proc. Natl. Acad. Sci. USA 1981, 78, 4256–4260. 6794030 10.1073/pnas.78.7.4256 PMC319768

[53] P. H. Gilligan , Clin. Microbiol. Rev. 1991, 4, 35–51. 1900735 10.1128/cmr.4.1.35 PMC358177

[54] J. J. Lipuma , Clin. Microbiol. Rev. 2010, 23, 299–323. 20375354 10.1128/CMR.00068-09 PMC2863368

[55] A. R. Hauser , M. Jain , M. Bar‐Meir , S. A. McColley , Clin. Microbiol. Rev. 2011, 24, 29–70. 21233507 10.1128/CMR.00036-10 PMC3021203

[56] J. M. Meyer , A. Stintzi , D. De Vos , P. Cornelis , R. Tappe , K. Taraz , H. Budzikiewicz , Microbiology 1997, 143, 35–43. 9025276 10.1099/00221287-143-1-35

[57] D. De Vos , M. De Chial , C. Cochez , S. Jansen , B. Tummler , J. M. Meyer , P. Cornelis , Arch. Microbiol. 2001, 175, 384–388. 11409549 10.1007/s002030100278

[58] J. S. Brooke , Clin. Microbiol. Rev. 2012, 25, 2–41. 22232370 10.1128/CMR.00019-11 PMC3255966

[59] A. M. Whatmore , A. Efstratiou , A. P. Pickerill , K. Broughton , G. Woodard , D. Sturgeon , R. George , C. G. Dowson , Infect. Immun. 2000, 68, 1374–1382. 10678950 10.1128/iai.68.3.1374-1382.2000 PMC97291

[60] J. Mitchell , Mol. Oral Microbiol. 2011, 26, 89–98. 21375700 10.1111/j.2041-1014.2010.00601.x

[61] L. H. Rasmussen , K. Hojholt , R. Dargis , J. J. Christensen , O. Skovgaard , U. S. Justesen , F. S. Rosenvinge , C. Moser , O. Lukjancenko , S. Rasmussen , X. C. Nielsen , J. Med. Microbiol. 2017, 66, 1316–1323. 28874232 10.1099/jmm.0.000573

[62] S. Gu , Z. Wei , Z. Shao , V. P. Friman , K. Cao , T. Yang , J. Kramer , X. Wang , M. Li , X. Mei , Y. Xu , Q. Shen , R. Kümmerli , A. Jousset , Nat. Microbiol. 2020, 5, 1002–1010. 32393858 10.1038/s41564-020-0719-8 PMC7116525

[63] E. Deriu , J. Z. Liu , M. Pezeshki , R. A. Edwards , R. J. Ochoa , H. Contreras , S. J. Libby , F. C. Fang , M. Raffatellu , Cell Host Microbe 2013, 14, 26–37. 23870311 10.1016/j.chom.2013.06.007 PMC3752295

[64] M. Ellermann , J. C. Arthur , Free Radic. Biol. Med. 2017, 105, 68–78. 27780750 10.1016/j.freeradbiomed.2016.10.489 PMC5401654

[65] W. Zhu , M. G. Winter , L. Spiga , E. R. Hughes , R. Chanin , A. Mulgaonkar , J. Pennington , M. Maas , C. L. Behrendt , J. Kim , X. Sun , D. P. Beiting , L. V. Hooper , S. E. Winter , Cell Host Microbe 2020, 27, 376–388. 32075741 10.1016/j.chom.2020.01.010 PMC7439322

[66] Y. Zhao , A. Bitzer , J. J. Power , D. Belikova , B. O. Torres Salazar , L. A. Adolf , D. Gerlach , B. Krismer , S. Heilbronner , ISME J. 2024, 18, wrae123. 38987933 10.1093/ismejo/wrae123 PMC11296517

[67] R. M. Stubbendieck , D. S. May , M. G. Chevrette , M. I. Temkin , E. Wendt‐Pienkowski , J. Cagnazzo , C. M. Carlson , J. E. Gern , C. R. Currie , Appl. Environ. Microbiol. 2019, 85, e02406–18. 30578265 10.1128/AEM.02406-18 PMC6498180

[68] H. Büttner , J. Hörl , J. Krabbe , C. Hertweck , ChemBioChem 2023, 24, e202300322. 37191164 10.1002/cbic.202300322

[69] M. Callaghan , S. McClean , Curr. Opin. Microbiol. 2012, 15, 71–77. 22137884 10.1016/j.mib.2011.11.001

