# Supplementary material for: Antibacterial Siderophores of Pandoraea Pathogens and Their Impact on the Diseased Lung Microbiota
Source: Angew Chem Int Ed Engl. 2025 Apr 14;64(24):e202505714. doi: 10.1002/anie.202505714 (PMC12144892; doi:10.1002/anie.202505714)
Supplement: Supplementary file 1 — Supporting Information [file ANIE-64-e202505714-s001.pdf]

# SUPPORTING INFORMATION

Table of Contents

Table of Contents ........ 1
Figures ........ 2
Tables ........ 4
General methods ........ 5
Experimental procedures ........ 6
Bacterial strains and culturing conditions ........ 6
Preparation of MM9 medium[1-3] ........ 6
Preparation of MM9 medium agar ........ 6
Siderophore production in liquid media and compound isolation ........ 6
Detection of the compounds ........ 7
Analysis of the putative *pan* biosynthetic gene cluster ........ 8
Extraction of sputum samples from cystic fibrosis patients ........ 10
Determination of the structure of pandorabactin A (1). ........ 12
Determination of the structure of pandorabactin B (2). ........ 15
Marfey's analysis of acid hydrolysates obtained from 1 and 2 ........ 18
Phylogenetic analysis of the C-domains from *pan* gene cluster ........ 18
Methylation and derivatization with the Mosher reagent ........ 19
Preparation of mutants of *P. oxalativorans* DSM 23570 ........ 20
Extraction of mutants of *P. oxalativorans* DSM 23570 ........ 21
Preparation of the synthetic substrate for the complementation assay ........ 21
Synthesis of $N^{\delta}$-benzoyloxy-$N^{\alpha}$-Boc-L-ornithine *tert*-butyl ester (13) ........ 22
Synthesis of $N^{\delta}$-benzoyloxy-$N^{\delta}$-(*R*)-3-hydroxy-butyryl-$N^{\alpha}$-Boc-L-ornithine *tert*-butyl ester (14) ........ 22
Synthesis of $N^{\delta}$-hydroxy-$N^{\delta}$-(*R*)-3-hydroxy-butyryl$N^{\alpha}$-Boc-L-ornithine *tert*-butyl ester (15) ........ 22
Synthesis of $N^{\delta}$-hydroxy-$N^{\delta}$-(*R*)-3-hydroxy-butyryl-L-ornithine (6) ........ 23
Chemical complementation of *P. oxalativorans* Δ*panQ* ........ 23
Construction of panQ heterologous expression plasmid ........ 24
Heterologous produciton and purification of PanQ ........ 25
Preparation of $N^{\delta}$-hydroxy-L-ornithine (5) and 3-(*R*)-Hydroxy-butyryl-CoA (7) ........ 25
*In vitro* reconstitutiuon of PanQ activity ........ 25
CAS assay solution preparation ........ 26
CAS agar preparation ........ 26
Preparation of $Fe^{3+}$-pandorabactin A (3) ........ 27

**Preparation of $Fe^{3+}$-pandorabactin B (4)** ..... 27

**Preparation of $Ga^{3+}$-pandorabactin A (8)** ..... 28

**Preparation of $Ga^{3+}$-pandorabactin B (9)** ..... 30

**Agar diffusion assay** ..... 32

**Minimal inhibitory concentration (MIC)** ..... 33

**MALDI mass spectrometry imaging** ..... 33

**Cultivation of monocultures of *P. aeruginosa*, *P. sputorum* and *P. oxalativorans* Δ*panI* and co-cultures** ..... 34

**Extraction of monocultures of *P. aeruginosa*, *P. sputorum* and *P. oxalativorans* Δ*panI* and co-cultures** ..... 34

**Metagenomic analysis** ..... 34

**NMR spectra of pandorabactin A (1)** ..... 36

**NMR spectra of pandorabactin B (2)** ..... 40

**NMR spectra of $N^{\delta}$-benzoyloxy-$N^{\alpha}$-Boc-L-ornithine *tert*-butyl ester (13)** ..... 44

**NMR spectra of $N^{\delta}$-benzoyloxy-$N^{\delta}$-(*R*)-3-hydroxy-butyryl-$N^{\alpha}$-Boc-L-ornithine *tert*-butyl ester (14)** ..... 45

**NMR spectrum of $N^{\delta}$-hydroxy-$N^{\delta}$-(*R*)-3-hydroxy-butyryl-$N^{\alpha}$-Boc-L-ornithine *tert*-butyl ester (15)** ..... 46

**NMR spectra of $N^{\delta}$-hydroxy-$N^{\delta}$-(*R*)-3-hydroxy-butyryl-L-ornithine (6)** ..... 47

**NMR spectrum of 3-(*R*)-hydroxy-butyryl-CoA (7)** ..... 48

**NMR spectra of $Ga^{3+}$-pandorabactin A (8)** ..... 49

**NMR spectra of $Ga^{3+}$-pandorabactin B (9)** ..... 53

**References** ..... 57

**Figures**

Figure S1. HRESI-MS/MS (positive ionization mode) spectrum of A pandorabactin A (**1**) *m/z* = 895.3873 $[M+H]^+$ and B pandorabactin B (**2**) *m/z* = 923.4186 $[M+H]^+$ from *P. oxalativorans*. ..... 7

Figure S2. HRESI-MS/MS (positive ionization mode) spectrum of A pandorabactin A (**1**) *m/z* = 895.3874 $[M+H]^+$ and B pandorabactin B (**2**) *m/z* = 923.4184 $[M+H]^+$ from *P. sputorum*. ..... 8

Figure S3. Putative *pan* biosynthetic gene cluster in genome from *Pandoraea sputorum*. ..... 8

Figure S4. HPLC-HRMS ($ESI^+$) (EIC, Extracted Ion Chromatogram, *m/z* 895.3891 ± 0.1 ppm) profiles of the pandorabactin A (**1**) as a standard and extracted sputum samples (**i**–**viii**). ..... 11

Figure S5. Calibration line of pandorabactin A (**1**) (left) and pandorabactin B (**2**) (right) for the determination of limit of detection (LOD). ..... 12

Figure S6. Structure of pandorabactin A (**1**). ..... 12

Figure S7. UV-Vis spectrum of pandorabactin A (**1**). ..... 12

Figure S8. FTIR spectrum of pandorabactin A (**1**). ..... 13

Figure S9. MS/MS fragmentation pattern of *m/z* 913.3997 $[M+H]^+$, linearized pandorabactin A (**1**). ..... 15

Figure S10. Structure of pandorabactin B (**2**). ..... 15

Figure S11. UV-Vis spectrum of pandorabactin B (**2**). ..... 16

Figure S12. FTIR spectrum of pandorabactin B (**2**). ..... 16

Figure S13. MS/MS fragmentation pattern of *m/z* 941.4304 $[M+H]^+$, linearized pandorabactin B (**2**). ..... 18

Figure S14. Phylogenetic analysis of the C-domains. ..... 19

Figure S15. PCR confirmation of *P. oxalativorans* mutants. .......... 21
Figure S16. HPLC-ESIMS (EIC, Extracted Ion Chromatogram, *m/z* 235.05 ± 0.2 ppm) profiles of the chemical substrate $N^{\delta}$-hydroxy-$N^{\delta}$-(*R*)-3-hydroxy-butyryl-L-ornithine (**6**), the *P. oxalativorans* Δ*panQ*, *P. oxalativorans* Δ*panI*, and the *P. oxalativorans* wild-type. Left panel indicates EIC profiles. Right panel shows observed ions of **6**. .......... 24
Figure S17. Purified $His_6$-PanQ and in vitro reconstitution of CoA-dependent $N^{\delta}$-hydroxy-L-ornithine (**5**) *N*-((*R*)-3-hydroxy-butryl)-transferase activity. (A) SDS-PAGE (4–20% gradient gel) of $His_6$-PanQ (44.98 kDa), which is purified by Ni-NTA resin. M: Molecular marker. (B) A schematic representation of in vitro reconstitution of PanQ function which transfers *N*-((*R*)-3-hydroxy-butryl) group of CoA thioester to $N^{\delta}$ of $N^{\delta}$-hydroxy-L-ornithine. (C) HPLC-ESIMS (EIC, Extracted Ion Chromatogram, *m/z* 235.05 ± 0.5 ppm) profiles of in vitro activity assay of PanQ. The left panel indicates EIC profiles. The right panel shows observed ions of **6** and **7**. .......... 26
Figure S18. CAS agar plate with cultivated a) *P. oxalativorans* wild-type, b) *P. oxalativorans* Δ*panI and* c) *P. oxalativorans* Δ*panQ*. .......... 27
Figure S19. UV-Vis spectrum of $Fe^{3+}$-pandorabactin A (**3**). .......... 27
Figure S20. UV-Vis spectrum of $Fe^{3+}$-pandorabactin B (**4**). .......... 28
Figure S21. $^{1}$H-NMR spectrum of pandorabactin A (**1**) in DMF-$d_7$ (600 MHz). .......... 36
Figure S22. $^{13}$C-NMR spectrum of pandorabactin A (**1**) in DMF-$d_7$ (150 MHz). .......... 36
Figure S23. $^{13}$C-Jmod-NMR spectrum of pandorabactin A (**1**) in DMF-$d_7$ (150 MHz, C and $CH_2$ are positive, CH and $CH_3$ are negative). .......... 37
Figure S24. $^{1}$H-$^{13}$C-HSQC spectrum of pandorabactin A (**1**) in DMF-$d_7$ (600 MHz). .......... 37
Figure S25. $^{1}$H-$^{13}$C-HMBC spectrum of pandorabactin A (**1**) in DMF-$d_7$ (600 MHz). .......... 38
Figure S26. $^{1}$H-$^{1}$H-COSY spectrum of pandorabactin A (**1**) in DMF-$d_7$ (600 MHz). .......... 38
Figure S27. $^{1}$H-$^{1}$H-NOESY spectrum of pandorabactin A (**1**) in DMF-$d_7$ (600 MHz). .......... 39
Figure S28. $^{1}$H-NMR spectrum of pandorabactin A (**1**) in DMSO-$d_6$ (600 MHz). .......... 39
Figure S29. $^{1}$H-NMR spectrum of pandorabactin B (**2**) in DMF-$d_7$ (600 MHz). .......... 40
Figure S30. $^{13}$C-NMR spectrum of pandorabactin B (**2**) in DMF-$d_7$ (150 MHz). .......... 40
Figure S31. $^{13}$C-Jmod-NMR spectrum of pandorabactin B (**2**) in DMF-$d_7$ (150 MHz, C and $CH_2$ are positive, CH and $CH_3$ are negative). .......... 41
Figure S32. $^{1}$H-$^{13}$C-HSQC spectrum of pandorabactin B (**2**) in DMF-$d_7$ (600 MHz). .......... 41
Figure S33. $^{1}$H-$^{13}$C-HMBC spectrum of pandorabactin B (**2**) in DMF-$d_7$ (600 MHz). .......... 42
Figure S34. $^{1}$H-$^{1}$H-COSY spectrum of pandorabactin B (**2**) in DMF-$d_7$ (600 MHz). .......... 42
Figure S35. $^{1}$H-$^{1}$H-NOESY spectrum of pandorabactin B (**2**) in DMF-$d_7$ (600 MHz). .......... 43
Figure S36. $^{1}$H-$^{1}$H-TOCSY spectrum of pandorabactin B (**2**) in DMF-$d_7$ (600 MHz). .......... 43
Figure S37. $^{1}$H-NMR spectrum of $N^{\delta}$-benzoyloxy-$N^{\alpha}$-Boc-L-ornithine *tert*-butyl ester (**13**) in $CDCl_3$ (500 MHz). .......... 44
Figure S38. $^{13}$C-NMR spectrum of $N^{\delta}$-benzoyloxy-$N^{\alpha}$-Boc-L-ornithine *tert*-butyl ester (**13**) in $CDCl_3$ (125 MHz). .......... 44
Figure S39. $^{1}$H-NMR spectrum of $N^{\delta}$-benzoyloxy-$N^{\delta}$-(*R*)-3-hydroxy-butyryl-$N^{\alpha}$-Boc-L-ornithine *tert*-butyl ester (**14**) in $CDCl_3$ (500 MHz). .......... 45
Figure S40. $^{13}$C-NMR spectrum of $N^{\delta}$-benzoyloxy-$N^{\delta}$-(*R*)-3-hydroxy-butyryl-$N^{\alpha}$-Boc-L-ornithine *tert*-butyl ester (**14**) in $CDCl_3$ (125 MHz). .......... 45
Figure S41. $^{1}$H-NMR spectrum of $N^{\delta}$-hydroxy-$N^{\delta}$-(*R*)-3-hydroxy-butyryl-$N^{\alpha}$-Boc-L-ornithine *tert*-butyl ester (**15**) in $CDCl_3$ (500 MHz). .......... 46
Figure S42. $^{1}$H-NMR spectrum of $N^{\delta}$-hydroxy-$N^{\delta}$-(*R*)-3-hydroxy-butyryl-L-ornithine (**6**) in $D_2O$ (500 MHz). .......... 47
Figure S43. $^{13}$C-NMR spectrum of $N^{\delta}$-hydroxy-$N^{\delta}$-(*R*)-3-hydroxy-butyryl-L-ornithine (**6**) in $D_2O$ (125 MHz). .......... 47
Figure S44. $^{1}$H NMR spectrum of 3-(*R*)-hydroxy-butyryl-CoA (**7**) in DMSO-$d_6$ (600 MHz). .......... 48
Figure S45. $^{1}$H-NMR spectrum of $Ga^{3+}$-pandorabactin A (**8**) in DMSO-$d_6$ (600 MHz). .......... 49
Figure S46. $^{13}$C-NMR spectrum of $Ga^{3+}$-pandorabactin A (**8**) in DMSO-$d_6$ (150 MHz). .......... 49

Figure S47. $^{13}$C-DEPT-NMR spectrum of $Ga^{3+}$-pandorabactin A (**8**) in DMSO-$d_6$ (150 MHz, $CH_2$ are negative, CH and $CH_3$ are positive). 50
Figure S48. $^{1}$H-$^{13}$C-HSQC spectrum of $Ga^{3+}$-pandorabactin A (**8**) in DMSO-$d_6$ (600 MHz). 50
Figure S49. $^{1}$H-$^{13}$C-HMBC spectrum of $Ga^{3+}$-pandorabactin A (**8**) in DMSO-$d_6$ (600 MHz). 51
Figure S50. $^{1}$H-$^{1}$H-COSY spectrum of $Ga^{3+}$-pandorabactin A (**8**) in DMSO-$d_6$ (600 MHz). 51
Figure S51. $^{1}$H-$^{1}$H-NOESY spectrum of $Ga^{3+}$-pandorabactin A (**8**) in DMSO-$d_6$ (600 MHz). 52
Figure S52. $^{1}$H-$^{1}$H-TOCSY spectrum of $Ga^{3+}$-pandorabactin A (**8**) in DMSO-$d_6$ (600 MHz). 52
Figure S53. $^{1}$H-NMR spectrum of $Ga^{3+}$-pandorabactin B (**9**) in DMSO-$d_6$ (600 MHz). 53
Figure S54. $^{13}$C-NMR spectrum of $Ga^{3+}$-pandorabactin B (**9**) in DMSO-$d_6$ (150 MHz). 53
Figure S55. $^{13}$C-Jmod-NMR spectrum of $Ga^{3+}$-pandorabactin B (**9**) in DMSO-$d_6$ (150 MHz, C and $CH_2$ are positive, CH and $CH_3$ are negative). 54
Figure S56. $^{1}$H-$^{13}$C-HSQC spectrum of $Ga^{3+}$-pandorabactin B (**9**) in DMSO-$d_6$ (600 MHz). 54
Figure S57. $^{1}$H-$^{13}$C-HMBC spectrum of $Ga^{3+}$-pandorabactin B (**9**) in DMSO-$d_6$ (600 MHz). 55
Figure S58. $^{1}$H-$^{1}$H-COSY spectrum of $Ga^{3+}$-pandorabactin B (**9**) in DMSO-$d_6$ (600 MHz). 55
Figure S59. $^{1}$H-$^{1}$H-NOESY spectrum of $Ga^{3+}$-pandorabactin B (**9**) in DMSO-$d_6$ (600 MHz). 56
Figure S60. $^{1}$H-$^{1}$H-TOCSY spectrum of $Ga^{3+}$-pandorabactin B (**9**) in DMSO-$d_6$ (600 MHz). 56

**Tables**

Table S1. Overview of bacterial strains. 6
Table S2. Deduced proteins from *pan* biosynthetic gene cluster. 9
Table S3. Predicted A-domain substrate specificity from *pan* NRPS genes from *P. sputorum*. X is unknown amino acid. 10
Table S4. Predicted A-domain substrate specificity from *pan* NRPS genes from *P. oxalativorans*. X is unknown amino acid. 10
Table S5. List of *Pandoraea* strains used for genomic analysis with antiSMASH version 7.0.0[4] possessing pandorabactin NRPS cluster. 10
Table S6. $^{1}$H- and $^{13}$C-NMR data of pandorabactin A (**1**) (600 MHz, DMF-$d_7$). 14
Table S7. $^{1}$H- and $^{13}$C-NMR data of pandorabactin B (**2**) (600 MHz, DMF-$d_7$). 17
Table S8. Retention times in min of reference compounds and pandorabactin A (**1**) and B (**2**) hydrolysate. 18
Table S9. Additional C-domains used for phylogeny analysis. 19
Table S10. The primers used in this study. Underlines show restriction sites. 20
Table S11. Plasmids used in this study. 21
Table S12. $^{1}$H- and $^{13}$C-NMR data of $Ga^{3+}$-pandorabactin A (**8**) (600 MHz, DMSO-$d_6$). 29
Table S13. $^{1}$H- and $^{13}$C-NMR data of $Ga^{3+}$-pandorabactin B (**9**) (600 MHz, DMSO-$d_6$). 31
Table S14. Agar diffusion assays with pandorabactin A (**1**) and pandorabactin B (**2**). 32
Table S15. Agar diffusion assays with $Fe^{3+}$-pandorabactin A (**3**) and $Fe^{3+}$-pandorabactin B (**4**). 33
Table S16. MIC values in $\mu g \cdot mL^{-1}$ of pandorabactin A (**1**) and pandorabactin B (**2**) on different test organisms. 33
Table S17. Determination of pyoverdine (**10**) in mono- and co-cultures. 34
Table S18. Fold-change values of the species detected in sputum samples from cystic fibrosis patients that differ significantly in abundancy when *pan* genes are present or absent. 35

## General methods

### HPLC-HRMS

High-resolution mass spectrometry measurements were performed on an Exactive Orbitrap High Performance Benchtop LC-MS (Thermo Fisher Scientific) with an electron spray ion (ESI) source and an Accela HPLC System, C18 column (Betasil C18 5 µm, 150 × 2.1 mm, Thermo Fisher Scientific), solvents: acetonitrile + 0.1% HCOOH and water + 0.1% HCOOH, flow rate: 0.2 mL · $min^{-1}$; program: hold 1 min at 5% acetonitrile, 1–16 min 5→98% acetonitrile, hold 3 min at 98% acetonitrile, 19–20 min 98%→5% acetonitrile, hold 13 min at 5% acetonitrile.

### HPLC-HRMS/MS

Tandem mass spectrometry measurements were performed on a QExactive Orbitrap High Performance Benchtop LC/MS (Thermo Fisher Scientific) with an electron spray ion (ESI) source and an Accela HPLC System, C18 column (Accucore C18 2.6 µm, 100 × 2.1 mm, Thermo Fisher Scientific) and the following solvents: acetonitrile + 0.1% HCOOH and water + 0.1% HCOOH at a flow rate of 0.2 mL · $min^{-1}$; gradient: 0–10 min 5→98% acetonitrile, hold 4 min at 98% acetonitrile, 14–14.1 min 98%→5% acetonitrile, hold 6 min at 5% acetonitrile.

### GC-MS

GC-MS analysis was performed using a Trace 1310 GC (Thermo Fisher Scientific) coupled with a TSQ 9000 electron impact (EI)-triple quad mass spectrometer (Thermo Fisher Scientific). The column was operated with helium carrier gas (1.5 mL · $min^{-1}$) and a split injection (split ratio 1:50). A BPX5 capillary column (30 m, 0.25 mm inner diameter, 0.25 µm film) from Trajan (SGE) was used. The GC temperature program was: 40 °C in 0–1 min, hold 1 min, heating to 100 °C in 1–3 min (30 C · $min^{-1}$), heating to 350 °C in 3–29 min, hold 1 min (10 °C · $min^{-1}$).

### NMR spectroscopy

NMR spectra were measured on Bruker Avance DRX 500 MHz or Bruker Avance III 600 MHz spectrometer equipped with a cryo-platform and signals were referenced to the residual solvent signal DMSO-$d_6$ (2.50 ppm ($^1$H) or 39.52 ppm ($^{13}$C)), DMF-$d_7$ (8.03 ppm, 2.92 ppm or 2.75 ppm ($^1$H) or 162.42 ppm, 34.89 ppm or 29.76 ppm ($^{13}$C)), $CDCl_3$ (7.26 ppm ($^1$H) or 77.16 ppm ($^{13}$C)) or $D_2O$ (4.79 ppm ($^1$H)) at a temperature of 300 K.

### UV-Vis spectroscopy

UV-Vis spectra were obtained using a UV-1800 spectrometer (Shimadzu) with quartz glass cuvette (type 100-QS) with 10 mm path length. The samples were measured at 20 °C in MeOH.

### IR spectroscopy

A Jasco Fourier Transform Infrared (FTIR) Spectrometer 4100 (Jasco) was used to measure the infrared spectra using the ATR technique.

### Optical rotation

Specific optical rotation of the enantiopure compounds was measured with a P-1020 (Jasco) in a quartz glass cuvette with 50 mm path length at a wavelength of 589 nm (Na-D line). The concentration was given in 10 mg/mL.

### Column chromatography

The substance purifications were performed using column chromatography. Silica gel (silica gel 60, 40–63 µm, Roth) was used as the stationary phase. The respective ratio of the eluents is specified in the individual experimental procedures.

### TLC

Thin layer chromatography (TLC) (silica gel 60 on aluminum plates coated with fluorescent indicator F254 nm, Merck) or HPLC-(HR)MS was used to monitor the reaction progress. TLC was stained with $KMnO_4$ (3 g $KMnO_4$, 20 g $K_2CO_3$, 2.5 mL 10% NaOH in 400 mL $H_2O$).

### General synthesis procedures

All chemicals and solvents were obtained from commercial suppliers (Sigma Aldrich, TCI, Alfa Aesar, ABCR, VWR) and used without further purification unless otherwise explained. Reactions were carried out in oven-dried glassware. An argon atmosphere was used for reactions under dry conditions.

## Experimental procedures

### Bacterial strains and culturing conditions

**Table S1.** Overview of bacterial strains.

| Species | Strian | Character | Source or reference |
|---|---|---|---|
| *Pandoraea oxalativorans* | DSM 23570 (CCM 7677 NBRC 106091) | Wild-type; isolated from soil litter close to Oxalis sp. | DSMZ |
| | Δ*NRPS* | $Cm^R$ gene inserted into NRPS gene | This study |
| | Δ*NAT* | $Cm^R$ gene inserted into NAT gene | This study |
| *Pandoraea sputorum* | DSM 21091 (CCUG 45026, LMG 18819, NCTC 13161, CIP 107268) | Wild-type; isolated from human sputum, cystic fibrosis patient | DSMZ |
| *Escherichia coli* | XL1 Blue | General cloning host strain | Stratagene |
| | TOP10 | General cloning host strain | Invitrogen |
| | BL21 (DE3) | Heterologous expression strain | NEB |
| | BL21 (DE3) pET28a-panQ | $His_6$-PanQ producing strain | This study |

### Preparation of MM9 medium[1-3]

One liter was prepared as follows. Solution A [350 g $K_2HPO_4$ and 100 g $KH_2PO_4$ dissolved in 1 L dd$H_2O$] and solution B [29.4 g NaCl, 50 g $(NH_4)_2SO_4$, 5 g $MgSO_4$ dissolved in 1 L dd$H_2O$] were prepared and autoclaved separately. To 900 mL dd$H_2O$, 2 g of an amino acid mixture [2 g each of L-Ala, L-Arg, L-Asn, L-Asp, L-Cys, L-Gln, L-Glu, Gly, L-Ile, L-Pro, L-Ser, L-Thr, L-Tyr, L-Val, L-Phe] were added and autoclaved. 20 mL of each solution A and B were added to the mixture. Further, 16.7 mL of L-Leu (100 mM), 5 mL of L-His (60 mM), 10 mL of L-Lys (100 mM), 10 mL of L-Trp (40 mM), 10 mL of L-Met (40 mM), and 20 mL of glucose (50% (*w/v*)) were added.
If necessary, 1 mL of a trace element solution [per liter: 40 mg $ZnCl_2$, 200 mg $FeCl_3 \cdot 6\,H_2O$, 10 mg $CuCl_2 \cdot 2\,H_2O$, 10 mg $MnCl_2 \cdot 4\,H_2O$, 10 mg $Na_2B_4O_7 \cdot 10\,H_2O$, 10 mg $(NH_4)_6Mo_7O_{24} \cdot 4\,H_2O$] was added.

### Preparation of MM9 medium agar

For preparation of 1 L MM9 medium agar the solution A and B were prepared as described above. To 900 mL dd$H_2O$, 2 g of an amino acid mixture [2 g each of L-Ala, L-Arg, L-Asn, L-Asp, L-Cys, L-Gln, L-Glu, Gly, L-Ile, L-Pro, L-Ser, L-Thr, L-Tyr, L-Val, L-Phe] and 30 g · $L^{-1}$ agar was added and autoclaved. After cooling to 60 °C 16.7 mL of L-Leu (100 mM), 5 mL of L-His (60 mM), 10 mL of L-Lys (100 mM), 10 mL of L-Trp (40 mM), 10 mL of L-Met (40 mM), and 20 mL of glucose (50% (*w/v*)) were added. 1 mL of a trace element solution [per liter: 40 mg $ZnCl_2$, 200 mg $FeCl_3 \cdot 6\,H_2O$, 10 mg $CuCl_2 \cdot 2\,H_2O$, 10 mg $MnCl_2 \cdot 4\,H_2O$, 10 mg $Na_2B_4O_7 \cdot 10\,H_2O$, 10 mg $(NH_4)_6Mo_7O_{24} \cdot 4\,H_2O$] was added.

### Siderophore production in liquid media and compound isolation

*P. oxalativorans* and *P. sputorum* were precultured in MM9 medium with trace elements with orbital shaking at 110 × *g* and 30 °C overnight, respectively. The obtained cultures were added to 1 L MM9 medium with trace elements, which was distributed in baffled Erlenmeyer flasks and orbital shaken at 110 × *g* and 30 °C for four days, with a start $OD_{600}$ of 0.05. For extraction Amberlite® XAD16 resin was used. The resin was prepared by washing it with methanol (3x) and acetone (2x) and equilibrated with dd$H_2O$. Before extraction the cultures were centrifuged at 5,000 × *g* for 15 min at room temperature. Then, the resin was added to the supernatant, and they were shaken at 110 × *g* and 30 °C for 1–2 h. The resin was filtered off and subsequently eluted with 10%-, 30%-, 50%-, 70%-, 100%- methanol solutions. Volatiles were removed under reduced pressure and the residual aqueous solution was freeze-dried. The samples were dissolved in MeOH for HPLC-HRMS analysis.
Fractions containing the desired compounds were pooled and subjected to preparative RP-HPLC using a Phenomenex Synergi Fusion-RP (250 × 21.20 mm, 4 µm, 80 Å) with a guard column and a gradient elution (solvent A: $H_2O$ + 0.01% TFA, solvent B: acetonitrile, time program: 3 min 10% B, 10% B to 25% B in 20 min, 25% B to 100% B in 2 min, 10 min 100% B, flow rate 16 mL · $min^{-1}$), $t_R$ = 12.2 min (pandorabactin A (**1**)) and $t_R$ = 21.9 min (pandorabactin B (**2**)). The collected fractions were lyophilized to yield 25.0 mg of pandorabactin A (**1**) and 16.4 mg of pandorabactin B (**2**) as a white powder, respectively.

**Pandorabactin A (1)**

**HRMS (ESI$^+$):** $[M+H]^+$ = 895.3873 (calculated for $C_{35}H_{59}N_8O_{19}$ 895.3891); **$[\alpha]_D^{21.4}$** = –9.42 (c = 0.5 in MeOH), **FTIR:** ν [cm$^{-1}$] = 3267.79, 1659.45, 1518.67, 1194.69, 1133.94.

**Pandorabactin B (2)**

**HRMS (ESI$^+$):** $[M+H]^+$ = 923.4186 (calculated for $C_{37}H_{63}N_8O_{19}$ 923.4204); **$[\alpha]_D^{21.4}$** = –15.86 (c = 0.25 in MeOH), **FTIR:** ν [cm$^{-1}$] = 3279.36, 1661.37, 1520.60, 1196.61, 1133.94.

**Detection of the compounds**

To compare the fragmentation pattern of the two compounds produced by *P. sputorum* and *P. oxalativorans* in MM9 medium, HRESI-MS/MS analysis was performed.

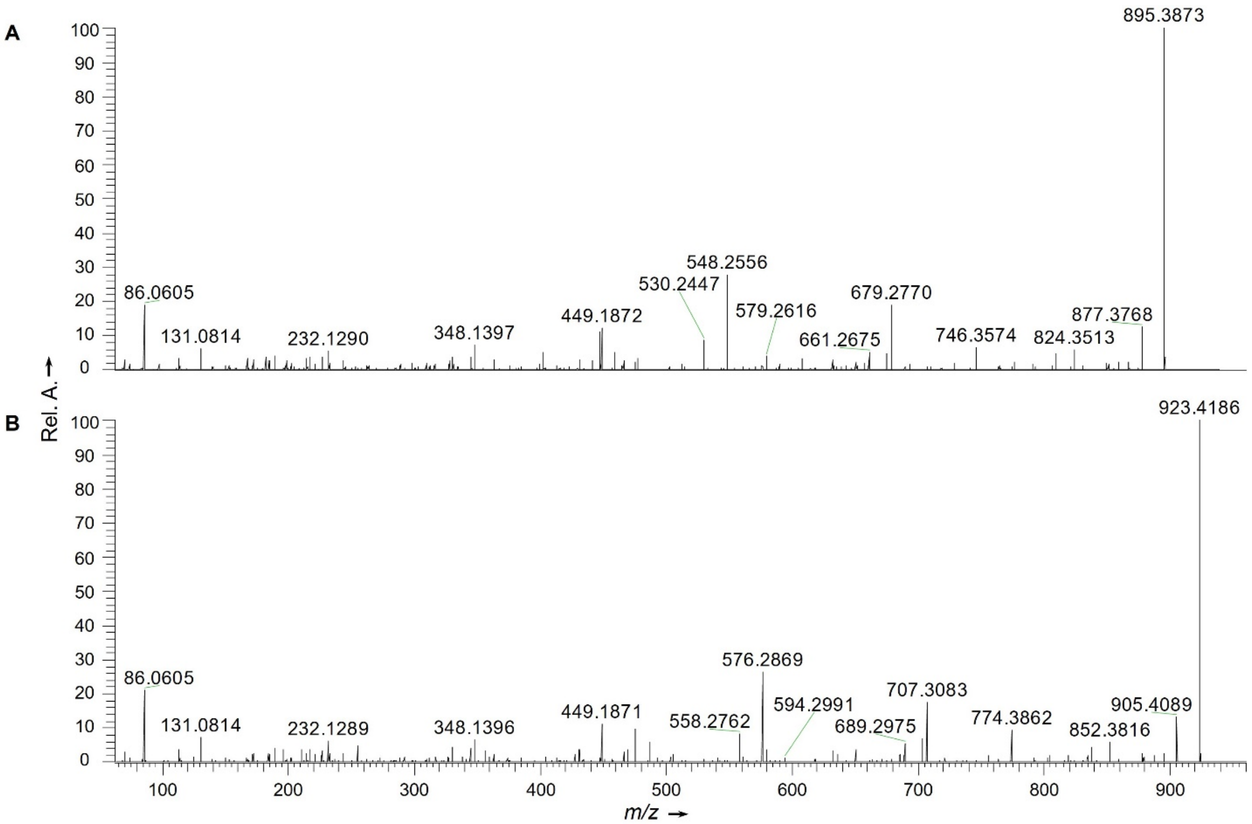


**Figure S1.** HRESI-MS/MS (positive ionization mode) spectrum of **A** pandorabactin A (**1**) *m/z* = 895.3873 $[M+H]^+$ and **B** pandorabactin B (**2**) *m/z* = 923.4186 $[M+H]^+$ from *P. oxalativorans*.

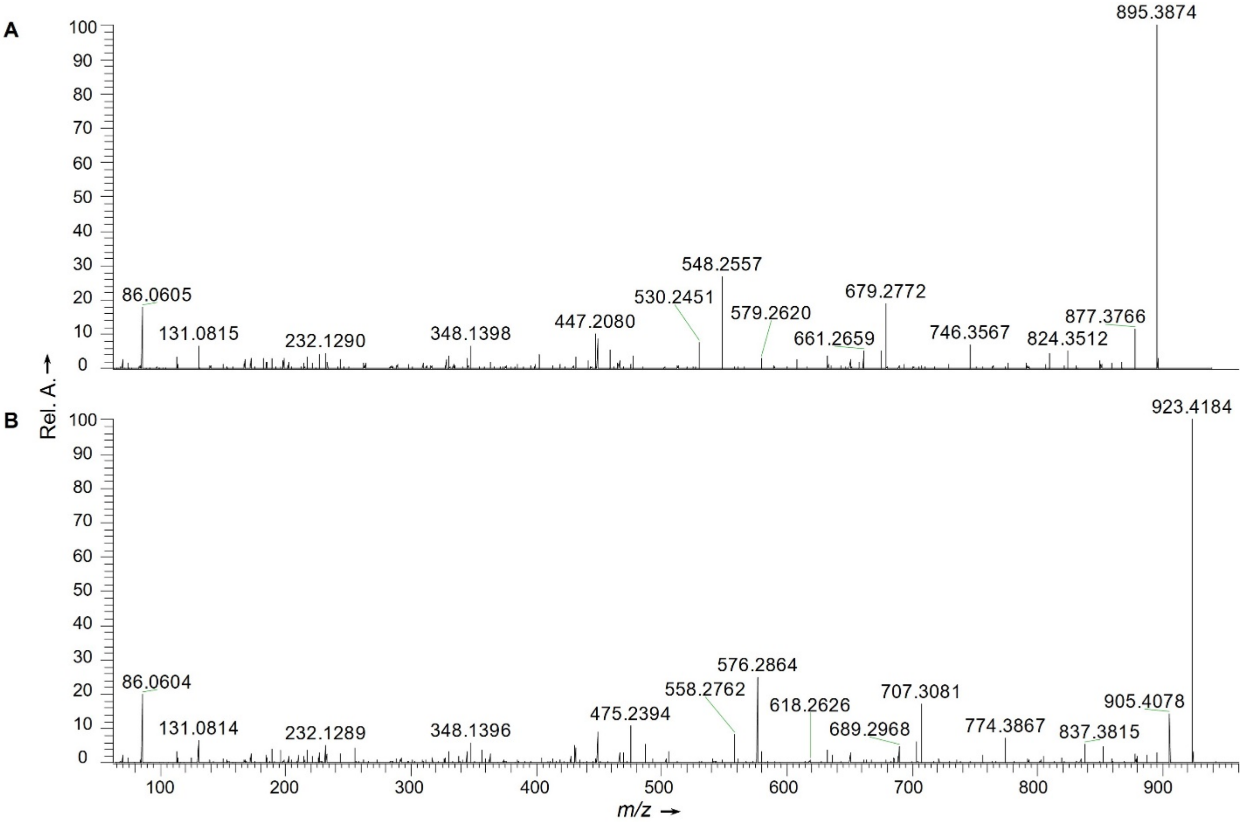


**Figure S2.** HRESI-MS/MS (positive ionization mode) spectrum of **A** pandorabactin A (**1**) *m/z* = 895.3874 [M+H]$^+$ and **B** pandorabactin B (**2**) *m/z* = 923.4184 [M+H]$^+$ from *P. sputorum*.

### Analysis of the putative *pan* biosynthetic gene cluster

Analyses of genome sequences of *P. sputorum* and *P. oxalativorans* were performed by antiSMASH version 7.0.0[4] and NCBI BLASTp[5] (database: non-redundant protein sequences). Strains showed analogous results. Predictions of A-domain substrate specificity were performed using antiSMASH version 7.0.0[4] and PKS/NRPS analysis tool.[6]

*Pandoraea sputorum*

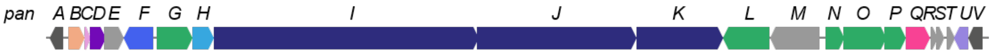


**Figure S3.** Putative *pan* biosynthetic gene cluster in genome from *Pandoraea sputorum*.

**Table S2.** Deduced proteins from *pan* biosynthetic gene cluster.

| Protein | Size (aa) | Putative function | Sequence (identity/similarity). | Query coverage | Accession No. |
|---|---|---|---|---|---|
| PanA | 190 | RNA polymerase sigma factor sigma-70 family | Full=RNA polymerase sigma factor WhiG (36%/54%) *Streptomyces coelicolor* A3(2) | 36% | P17211.1 |
| PanB | 245 | 4'-phosphopantetheinyl transferase superfamily protein | 4'-phosphopantetheinyl transferase superfamily protein (43%/52%) *Burkholderia cenocepacia* | 74% | WP_109341512.1 |
| PanC | 75 | MbtH family protein | MbtH family protein (74%/86%) *Marinobacter lutaoensis* | 88% | WP_076725231.1 |
| PanD | 234 | alpha/beta fold hydrolase | Chain A, Thioesterase (36%/50%) *Streptomyces* sp. WAC02707 | 89% | 6VAP_A |
| PanE | 301 | Siderophore-iron reductase FhuF | siderophore-iron reductase FhuF (43%/56%) *Paraburkholderia caribensis* | 72% | WP_060606979.1 |
| PanF | 458 | lysine N(6)-hydroxylase/L-ornithine N(5)-oxygenase family protein | SidA/IucD/PvdA family monooxygenase (55%/69%) *Pseudomonas lundensis* | 92% | WP_169880469.1 |
| PanG | 566 | cyclic peptide export ABC transporter | Chain B, Drug ABC transporter ATP-binding protein (26%/44%) *Mycolicibacterium thermoresistibile* | 47% | 6TEJ_B |
| PanH | 324 | TauD/TfdA family dioxygenase | TauD/TfdA family dioxygenase (73%/82%) *Pseudomonas mosselii* | 96% | WP_062363879.1 |
| PanI | 4425 | NRPS (CAL-C-A-E-C-A-C-A) | Chain A, linear gramicidin synthase subunit A (32%/49%) *Brevibacillus parabrevis* | 97% | 6MFZ_A |
| PanJ | 2650 | NRPS (C-A-E-C-A) | Chain A, Txo2 (38%/52%) *Eleftheria terrae* | 79% | 6P1J_A |
| PanK | 1411 | amino acid adenylation domain-containing protein | Chain A, Txo2 (38%/52%) *Eleftheria terrae* | 66% | 6P1J_A |
| PanL | 746 | TonB-dependent siderophore receptor | Chain A, Enantio-pyochelin receptor (41%/57%) *Pseudomonas fluorescens* | 97% | 3QLB_A |
| PanM | 804 | penicillin acylase family protein | Chain A, Protein related to penicillin acylase (40%/55%) *Acidovorax* sp. MR-S7 | 98% | 5C9I_A |
| PanN | 288 | ABC transporter ATP-binding protein | ABC transporter ATP-binding protein (61%/74%) *Paraburkholderia sp.* D15 | 89% | WP_281001275.1 |
| PanO | 701 | iron ABC transporter permease | Chain B, Iron(III)-hydroxamate import system permease protein FhuB(33%/51%) *Escherichia coli* K-12 | 87% | 7LB8_B |
| PanP | 333 | iron-siderophore ABC transporter substrate-binding protein | Hypothetical protein GQ56_0121240 (49%/64%) *Burkholderia paludis* | 81% | KFG95296.1 |
| PanQ | 371 | GNAT family N-acetyltransferase | GNAT family N-acetyltransferase (50%/61%) *Arboricoccus pini* | 96% | WP_207762091.1 |
| PanR | 88 | BrnT family toxin | BrnT family toxin (69%/81%) *Sulfuriferula nivalis* | 98% | WP_232526050.1 |
| PanS | 113 | BrnA antitoxin family protein | - | - | - |
| PanT | 106 | hypothetical protein | - | - | - |
| PanU | 205 | GNAT family protein | GNAT family protein (79%/86%) *Ralstonia* sp. UNC404CL21Col | 95% | WP_027679393.1 |
| PanV | 223 | Crp/Fnr family transcriptional regulator | Chain A, Transcriptional regulator, FNR/CRP family (26%/43%) *Thermus thermophilus* | 79% | 2ZCW_A |

**Table S3.** Predicted A-domain substrate specificity from *pan* NRPS genes from *P. sputorum*. X is unknown amino acid.

| A-domain | Stachelhaus code | antiSMASH | PKS/NRPS analysis |
|---|---|---|---|
| A1 | D G E G S G G M | X | 5hOrn |
| A2 | D L T K V G H V | Asp | Asp |
| A3 | D F W N V G M V | Thr | Thr |
| A4 | D G E G S G G V T | X | 5hOrn |
| A5 | D V W C V A M V | Ala | X |
| A6 | D L T K I G H V | X | Asp |

**Table S4.** Predicted A-domain substrate specificity from *pan* NRPS genes from *P. oxalativorans*. X is unknown amino acid.

| A-domain | Stachelhaus code | antiSMASH | PKS/NRPS analysis |
|---|---|---|---|
| A1 | D G E G S G G M | X | 5hOrn |
| A2 | D L T K V G H V | Asp | Asp |
| A3 | D F W N V G M V | Thr | Thr |
| A4 | D G E G S G G V T | X | 5hOrn |
| A5 | D V W C V A M V | Ala | X |
| A6 | D L T K I G H V | Asn | Asp |

**Table S5.** List of *Pandoraea* strains used for genomic analysis with antiSMASH version 7.0.0[4] possessing pandorabactin NRPS cluster.

| Bacterial organism | Strain | Genome accession No. | Isolation source |
|---|---|---|---|
| *Pandoraea sputorum* | DSM 21091 | NZ_CP010431.2 | human sputum, cystic fibrosis patient |
| *Pandoraea oxalativorans*# | DSM 23570 | NZ_CP011253.3 | soil litter close to *Oxalis* sp. |
| *Pandoraea anapnoica* | LMG 31117 | GCF_902459765.1 | human throat, cystic fibrosis patient |
| *Pandoraea anhela* | LMG 31108 | GCF_902459655.1 | human throat, cystic fibrosis patient |
| *Pandoraea aquatica* | LMG 31011 | GCF_902459565.1 | water, greenhouse pond |
| *Pandoraea captiosa* | LMG 31118 | GCF_902459775.1 | human sputum, cystic fibrosis patient |
| *Pandoraea commovens* | LMG 31010 | GCF_902459615.1 | cystic fibrosis patient |
| *Pandoraea pulmonicola* | DSM 16583 | GCF_000815105.2 | sputum, cystic fibrosis patient |
| *Pandoraea terrigena* | LMG 31013 | GCF_902459705.1 | soil |
| *Pandoraea vervacti* | DSM 23571 | NZ_CP010897.2 | uncultivated field soil |

#: strain from which pandorabactin A (**1**) and B (**2**) were isolated.

### Extraction of sputum samples from cystic fibrosis patients

Sputum samples were collected at Heidelberg University Hospital and freeze-dried on the day of the visit.[7] Eight samples (approx. 250 µL) were freeze-dried and the residues were suspended in MeOH (1 mL) and allowed to stand for 1 h. The solutions were filtered and dried under nitrogen flow. The samples were redissolved in 20 µL MeOH for HPLC-HRMS analysis.

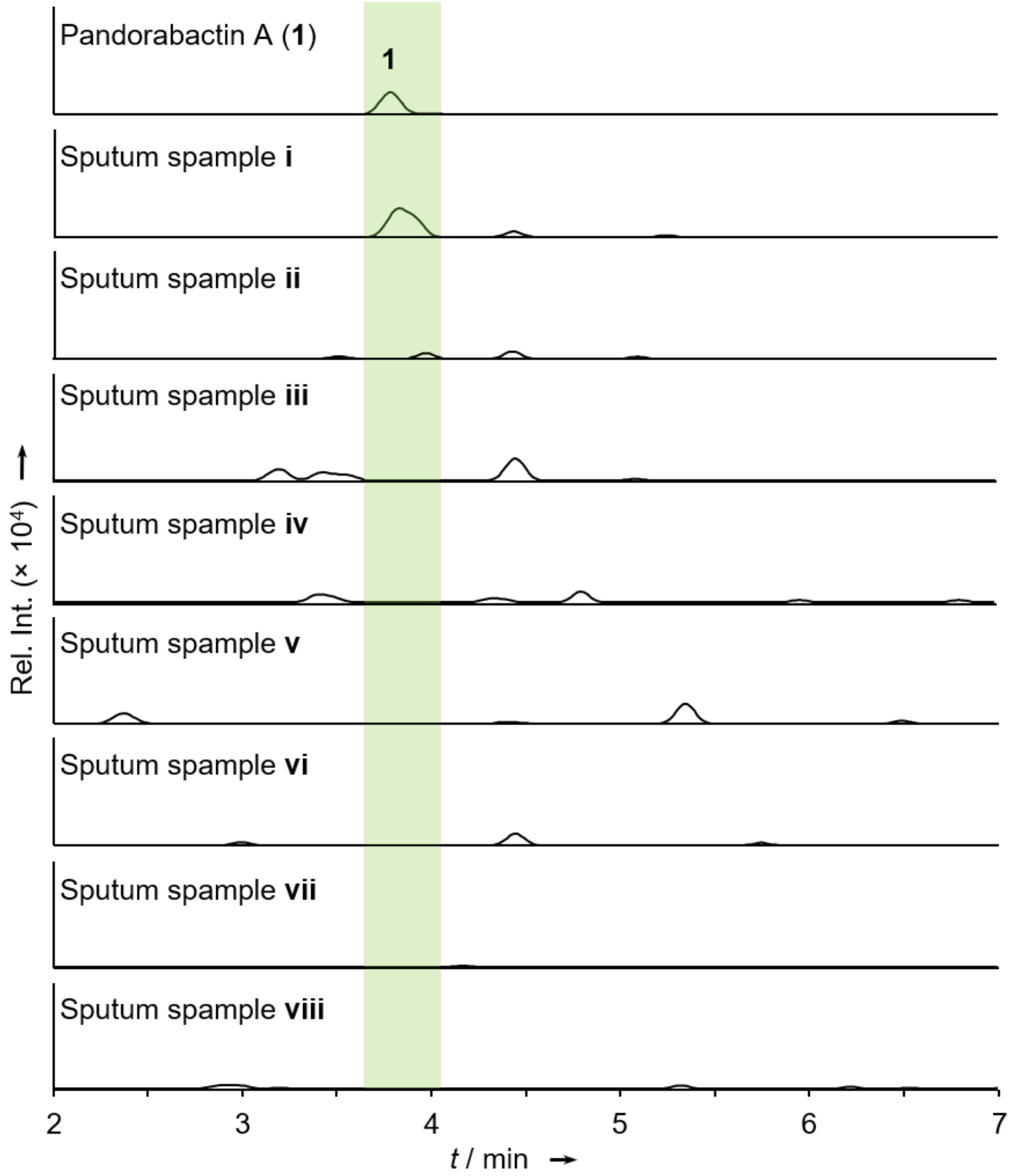


**Figure S4.** HPLC-HRMS ($ESI^+$) (EIC, Extracted Ion Chromatogram, *m/z* 895.3891 ± 0.1 ppm) profiles of the pandorabactin A (**1**) as a standard and extracted sputum samples (**i**–**viii**).

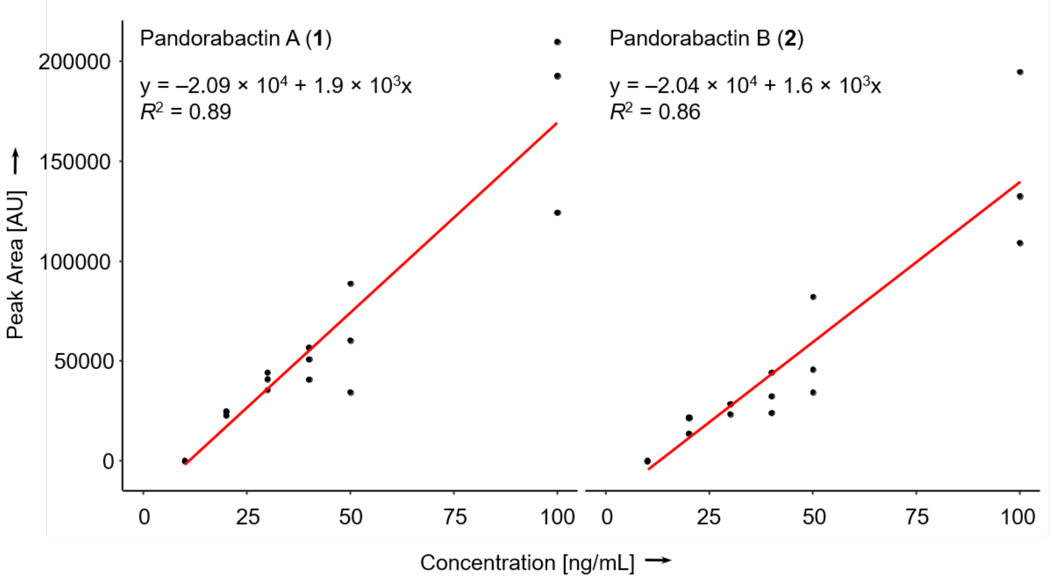


**Figure S5.** Calibration line of pandorabactin A (**1**) (left) and pandorabactin B (**2**) (right) for the determination of limit of detection (LOD).

### Determination of the structure of pandorabactin A (1).

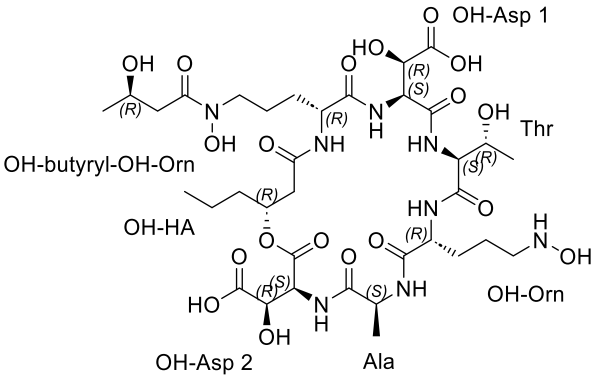


**Figure S6.** Structure of pandorabactin A (**1**), OH-HA, 3-OH-hexanoic acid; OH-butyryl-OH-Orn, *N*$^{\delta}$-3-OH-butyryl-*N*$^{\delta}$-OH-D-ornithine; OH-Asp 1, L-*erythro*-3-OH-aspartate 1; Thr, threonine, OH-Orn, *N*$^{\delta}$-OH-D-ornithine; Ala, alanine; OH-Asp 2, L-*erythro*-3-OH-aspartate 2.

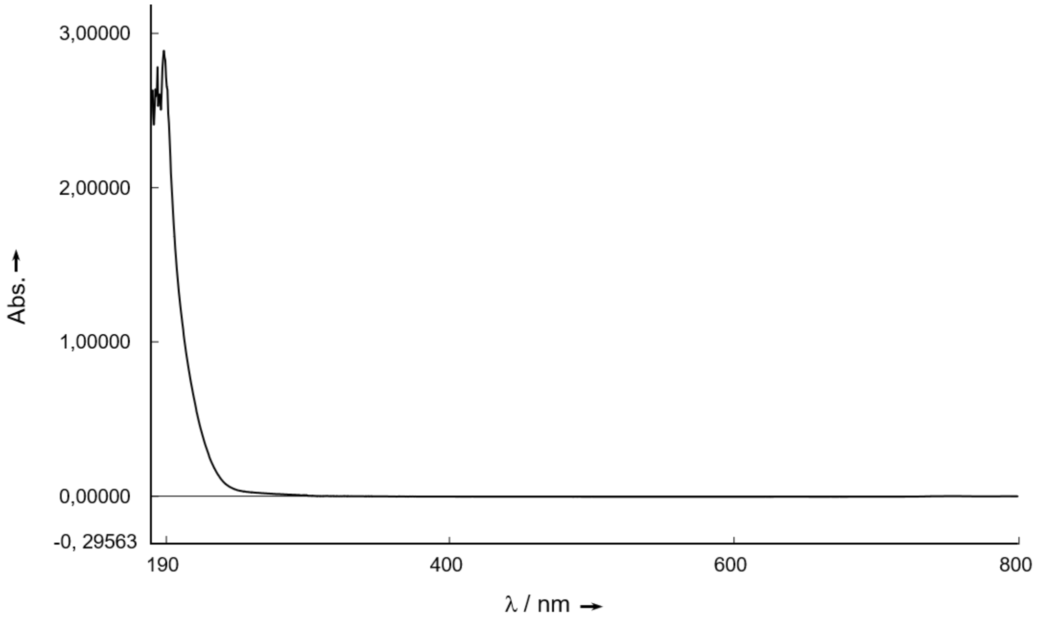


**Figure S7.** UV-Vis spectrum of pandorabactin A (**1**). Spectrum was measured in MeOH with a concentration of 50.0 µg/mL.

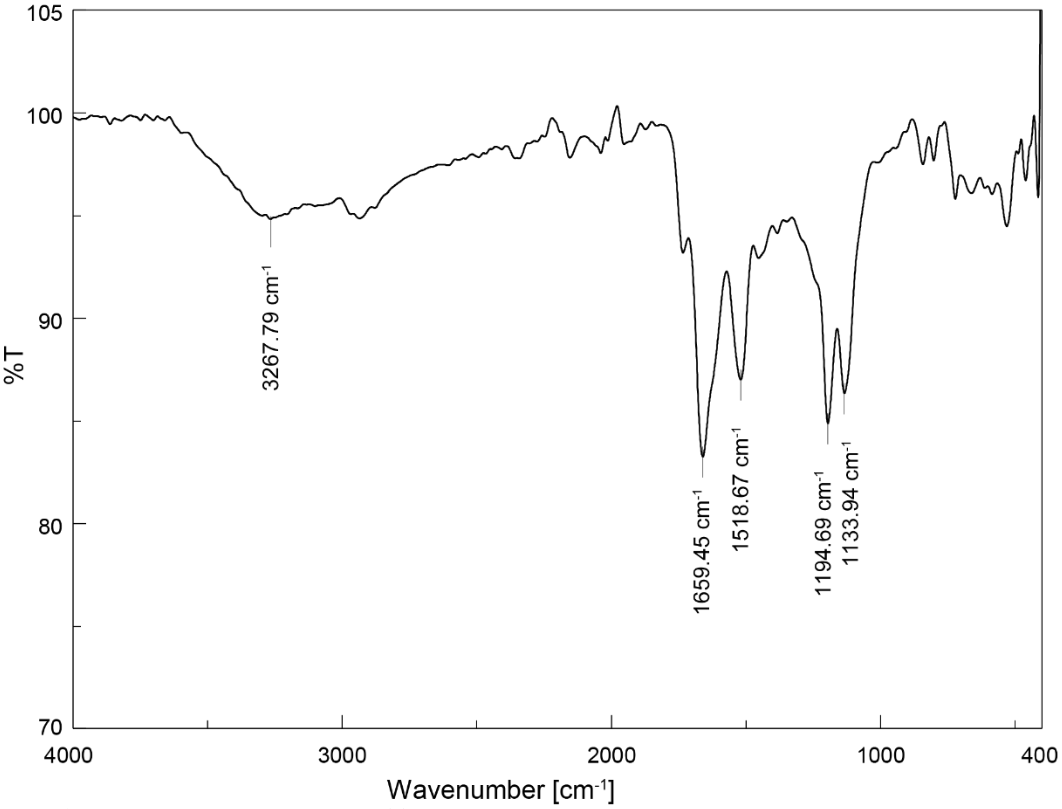


**Figure S8.** FTIR spectrum of pandorabactin A (**1**).

**Table S6.** $^{1}$H- and $^{13}$C-NMR data of pandorabactin A (**1**) (600 MHz, DMF-$d_7$).

| Partial structure | Position | $\delta_C$ [ppm] (m) | $\delta_H$ [ppm] (m, *J* [Hz]) |
|---|---|---|---|
| 3-OH-Hexanoic acid | CO | 170.7 (C) | - |
| | $C_\alpha$ | 37.7 ($CH_2$) | 2.96 (1H, m), 2.51 (1H, d, 14.2) |
| | $C_\beta$ | 73.6 (CH) | 5.33 (1H, m) |
| | $C_\gamma$ | 36.7 ($CH_2$) | 1.64 (1H, m), 1.55 (1H, m) |
| | $C_\delta$ | 18.4 ($CH_2$) | 1.40 (1H, m), 1.33 (1H, m) |
| | $C_\varepsilon$ | 13.7 ($CH_3$) | 0.88 (3H, t, 7.3) |
| $N^\delta$-3-OH-Butyryl-$N^\delta$-OH-D-ornithine | NH | - | 8.55 (1H, br. s) |
| | CO | 170.5 (C) | - |
| | $C_\alpha$ | 53.2 (CH) | 4.39 (1H, m) |
| | $C_\beta$ | 29.0 ($CH_2$) | 2.01 (2H, m) |
| | $C_\gamma$ | 22.4 ($CH_2$) | 1.86 (2H, m) |
| | $C_\delta$ | 47.0 ($CH_2$) | 3.72 (1H, m), 3.63 (1H, m) |
| | CO | 172.6 (C) | - |
| | $C'_\alpha$ | 41.6 ($CH_2$) | 2.66 (1H, dd, 15.2, 7.3), 2.57 (1H, dd, 15.2, 5.4) |
| | $C'_\beta$ | 64.2 (CH) | 4.16 (1H, m) |
| | $C'_\gamma$ | 23.2 ($CH_3$) | 1.16 (3H, d, 6.2) |
| L-*erythro*-3-OH-Aspartate 1 | NH | - | 9.21 (1H, d, 6.9) |
| | CO | 169.2 (C) | - |
| | $C_\alpha$ | 57.2 (CH) | 4.92 (1H, dd, 7.5, 4.9) |
| | $C_\beta$ | 71.6 (CH) | 4.47 (1H, d, 4.8) |
| | COOH | 172.9 (C) | - |
| L-Threonine | NH | - | 7.89 (1H, d, 7.2) |
| | CO | 171.2 (C) | - |
| | $C_\alpha$ | 60.1 (CH) | 4.34 (1H, dd, 7.0, 4.2) |
| | $C_\beta$ | 67.2 (CH) | 4.26 (1H, m) |
| | $C_\gamma$ | 19.7 ($CH_3$) | 1.20 (3H, d, 6.3) |
| $N^\delta$-OH-D-Ornithine | NH | - | 8.04 (1H, m) |
| | CO | 172.5 (C) | - |
| | $C_\alpha$ | 54.2 (CH) | 4.29 (1H, m) |
| | $C_\beta$ | 29.5 ($CH_2$) | 1.86 (1H, m), 1.66 (1H, m) |
| | $C_\gamma$ | 22.6 ($CH_2$) | 1.86 (1H, m), 1.54 (1H, m) |
| | $C_\delta$ | 47.9 ($CH_2$) | 4.06 (1H, m), 3.15 (1H, m) |
| L-Alanine | NH | - | 8.10 (1H, d, 6.6) |
| | CO | 173.2 (C) | - |
| | $C_\alpha$ | 50.9 (CH) | 4.21 (1H, m) |
| | $C_\beta$ | 16.7 ($CH_3$) | 1.32 (3H, d, 7.4) |
| L-*erythro*-3-OH-Aspartate 2 | NH | - | 7.30 (1H, d, 6.4) |
| | CO | 169.0 (C) | - |
| | $C_\alpha$ | 56.6 (CH) | 4.87 (1H, dd, 6.3, 2.3) |
| | $C_\beta$ | 71.3 (CH) | 4.55 (1H, d, 2.3) |
| | COOH | 172.6 (C) | - |

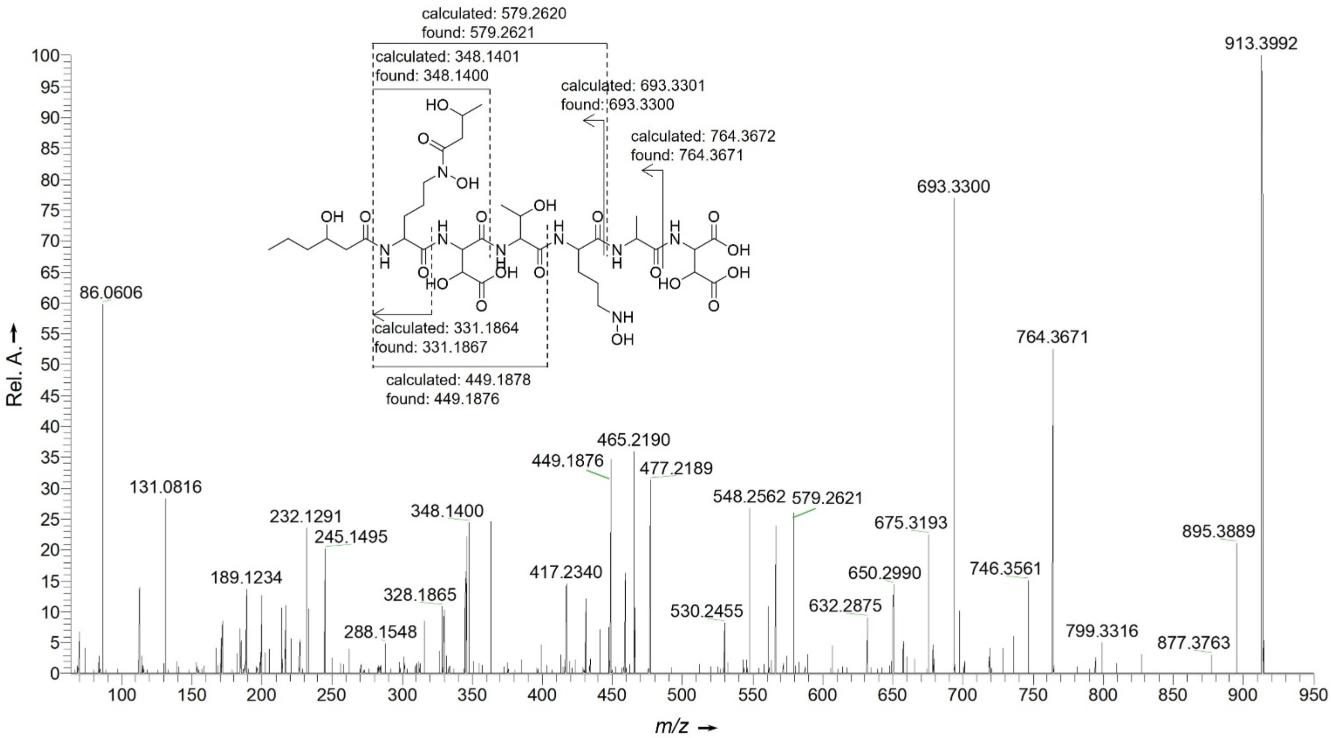


**Figure S9.** MS/MS fragmentation pattern of *m/z* 913.3997 [M+H]+, linearized pandorabactin A (**1**).

### Determination of the structure of pandorabactin B (2)

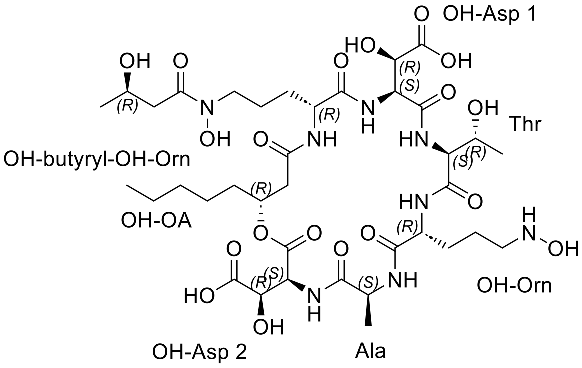


**Figure S10.** Structure of pandorabactin B (**2**), OH-OA, 3-OH-octanoic acid; OH-butyryl-OH-Orn, $N^{\delta}$-3-OH-butyryl-$N^{\delta}$-OH-D-Ornithine; OH-Asp 1, L-*erythro*-3-OH-Aspartate 1; Thr, Threonine, OH-Orn, $N^{\delta}$-OH-D-Ornithine; Ala, Alanine; OH-Asp 2, L-*erythro*-3-OH-Aspartate 2.

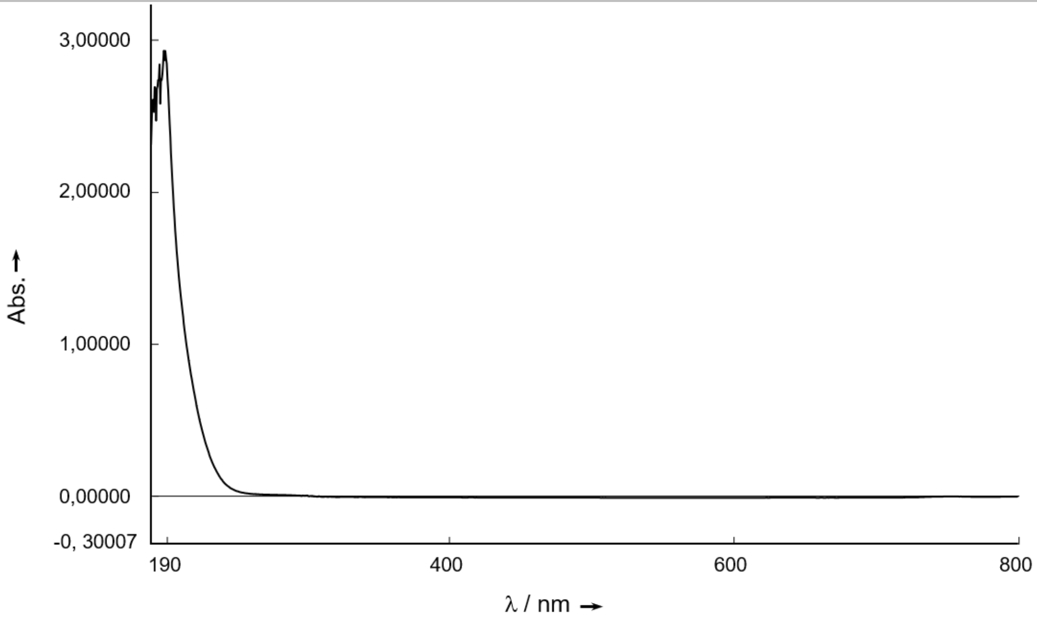


**Figure S11.** UV-Vis spectrum of pandorabactin B (**2**). Spectrum was measured in MeOH with a concentration of 50.0 µg/mL.

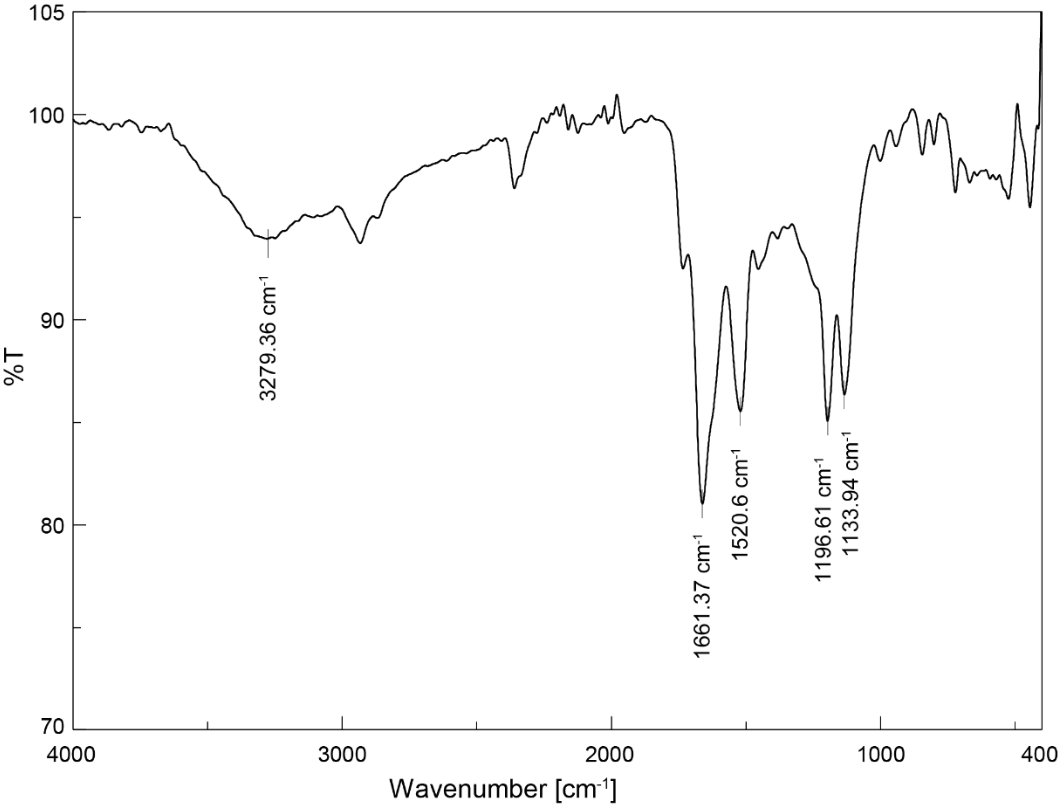


**Figure S12.** FTIR spectrum of pandorabactin B (**2**).

**Table S7.** $^{1}$H- and $^{13}$C-NMR data of pandorabactin B (**2**) (600 MHz, DMF-$d_7$).

| Partial structure | Position | $\delta_C$ [ppm] (m) | $\delta_H$ [ppm] (m, *J* [Hz]) |
|---|---|---|---|
| 3-OH-Octanoic acid | CO | 170.8 (C) | - |
| | $C_\alpha$ | 37.5 ($CH_2$) | 3.00 (1H, m), 2.50 (1H, d, 14.3) |
| | $C_\beta$ | 74.1 (CH) | 5.29 (1H, m) |
| | $C_\gamma$ | 34.5 ($CH_2$) | 1.62 (1H, m), 1.29 (1H, m) |
| | $C_\delta$ | 24.9 ($CH_2$) | 1.39 (1H, m), 1.29 (1H, m) |
| | $C_\varepsilon$ | 31.8 ($CH_2$) | 1.29 (1H, m), 1.26 (1H, m) |
| | $C_\zeta$ | 22.5 ($CH_2$) | 1.52 (1H, m), 1.29 (1H, m) |
| | $C_\eta$ | 13.8 ($CH_3$) | 0.87 (3H, t, 7.1) |
| $N^\delta$-3-OH-Butyryl-$N^\delta$-OH-D-ornithine | NH | - | 8.58 (1H, br. s) |
| | CO | 171.1 (C) | - |
| | $C_\alpha$ | 53.2 (CH) | 4.40 (1H, br. s) |
| | $C_\beta$ | 28.9 ($CH_2$) | 2.03 (2H, m) |
| | $C_\gamma$ | 22.3 ($CH_2$) | 1.90 (2H, m) |
| | $C_\delta$ | 46.9 ($CH_2$) | 3.74 (1H, m), 3.64 (1H, m) |
| | CO | 172.6 (C) | - |
| | $C'_\alpha$ | 41.6 ($CH_2$) | 2.66 (1H, dd, 15.2, 7.4), 2.57 (1H, dd, 15.2, 5.2) |
| | $C'_\beta$ | 64.1 (CH) | 4.16 (1H, m) |
| | $C'_\gamma$ | 23.2 ($CH_3$) | 1.16 (3H, d, 6.2) |
| L-*erythro*-3-OH-Aspartate 1 | NH | - | 9.26 (1H, m) |
| | CO | 169.1 (C) | - |
| | $C_\alpha$ | 56.7 (CH) | 4.89 (1H, m) |
| | $C_\beta$ | 71.4 (CH) | 4.56 (1H, d, 3.5) |
| | COOH | 172.9 (C) | - |
| L-Threonine | NH | - | 8.04 (overlapping with solvent signal) |
| | CO | 171.2 (C) | - |
| | $C_\alpha$ | 60.3 (CH) | 4.35 (1H, m) |
| | $C_\beta$ | 67.2 (CH) | 4.31 (1H, m) |
| | $C_\gamma$ | 20.0 ($CH_3$) | 1.21 (3H, d, 6.3) |
| $N^\delta$-OH-D-Ornithine | NH | - | 7.87 (1H, m) |
| | CO | 172.1 (C) | - |
| | $C_\alpha$ | 54.1 (CH) | 4.26 (1H, m) |
| | $C_\beta$ | 29.6 ($CH_2$) | 1.90 (1H, m), 1.61 (1H, m) |
| | $C_\gamma$ | 22.5 ($CH_2$) | 1.90 (1H, m), 1.53 (1H, m) |
| | $C_\delta$ | 47.8 ($CH_2$) | 4.20 (1H, m), 3.10 (1H, m) |
| L-Alanine | NH | - | 8.19 (1H, d, 6.7) |
| | CO | 173.3 (C) | - |
| | $C_\alpha$ | 50.9 (CH) | 4.21 (1H, m) |
| | $C_\beta$ | 16.9 ($CH_3$) | 1.33 (3H, d, 7.3) |
| L-*erythro*-3-OH-Aspartate 2 | NH | - | 7.36 (1H, d, 6.3) |
| | CO | 169.2 (C) | - |
| | $C_\alpha$ | 57.7 (CH) | 4.88 (1H, m) |
| | $C_\beta$ | 71.5 (CH) | 4.50 (1H, d, 3.5) |
| | COOH | 172.7 (C) | - |

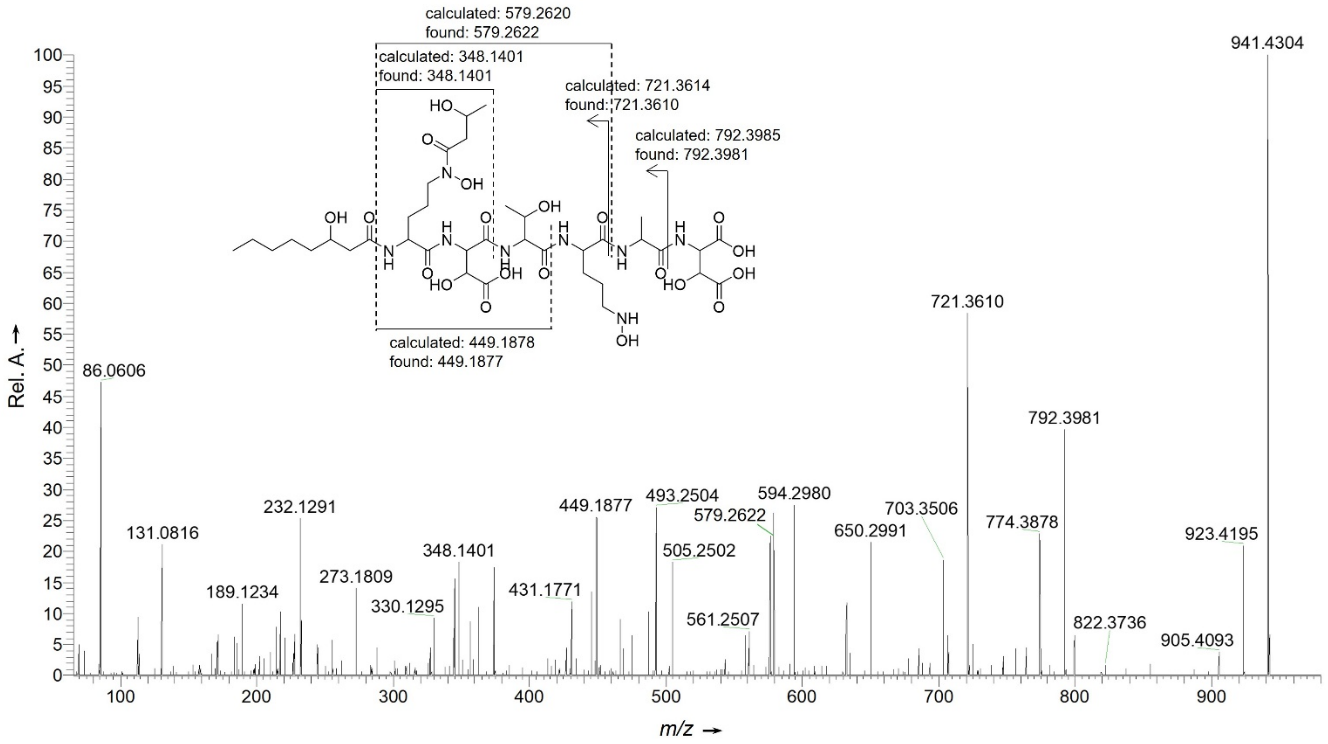


**Figure S13.** MS/MS fragmentation pattern of *m/z* 941.4304 $[M+H]^+$, linearized pandorabactin B (**2**).

### Marfey's analysis of acid hydrolysates obtained from 1 and 2

For determination of absolute configuration of amino acids from pandorabactin A (**1**) and B (**2**), 1-fluoro-2,4-dinitrophenyl-5-L-alanine amide (L-FDAA) was used. **1** and **2** (1.5 to 2.0 mg) were hydrolyzed with 500 µL of 6 M HCl or conc. HI at 105 °C for 16 h, respectively. The resulting mixture of amino acids were dried under reduced pressure and redissolved in 100 µL of water and then 50 µL of 1 M $NaHCO_3$ were added. Further 10 µL of a 10 mg · $mL^{-1}$ solution of L-FDAA in acetone were added to the reaction solution. The reaction was stirred for 1 h at 40 °C. To quench the reaction 25 µL of 2 M HCl were added to the mixture. To dilute the reaction mixture 25 µL methanol were added. A 1:2 dilution of the mixtures was analyzed using LC-MS with a reversed-phase HPLC column (Betasil $C_{18}$ 5 µm, 150 × 2.1 mm) and an elution gradient (solvent A: $H_2O$ + 0.1% HCOOH, solvent B: acetonitrile + 0.1% HCOOH, time program: 1 min 5% B, 5% B to 98% B in 15 min, 3 min 98% B, 98% B to 5% B in 1 min, 13 min 5% B, flow rate: 0.2 mL · $min^{-1}$). For analysis of D/L-*erythro*-OH-Asp an elution gradient (solvent A: $H_2O$ + 0.1% HCOOH, solvent B: acetonitrile + 0.1% HCOOH, time program: 1 min 5% B, 5% B to 28% B in 2 min, 15 min 28% B, 28% B to 98% B in 3 min, 3 min 98% B, 98% B to 5% B in 1 min, 9 min 5% B, flow rate: 0.2 mL · $min^{-1}$) was used. The elution order of D/L-*erythro*-OH-Asp was shown before to be D→ L.[8]

**Table S8.** Retention times in min of reference compounds and pandorabactin A (**1**) and B (**2**) hydrolysate.

| | D-Orn | L-*erythro*-OH-Asp | L-Thr | L-Ala |
|---|---|---|---|---|
| Reference | 8.19 and 8.65 | 10.37 | 9.79 | 10.55 |
| pandorabactin A hydrolysate | 8.20 and 8.65 | 10.30 | 9.69 | 10.52 |
| pandorabactin B hydrolysate | 8.21 and 8.67 | 10.34 | 9.67 | 10.52 |

### Phylogenetic analysis of the C-domains from *pan* gene cluster

C-domain amino acid sequences were downloaded from the NaPDos2[9] and added to C-domain protein sequences from pandorabactin A (**1**) and B (**2**) according to antiSMASH version 7.0.0[4]. Additional C-domains were added to the NAPDos2 amino sequences, and they are summarized in table S6. The sequence alignment was done using MAFFT 7[10-11] with default settings. Maximum likelihood phylogeny was constructed using the IQ-tree web server with default settings.[12] The tree was displayed with the program MEGA 7.[13]

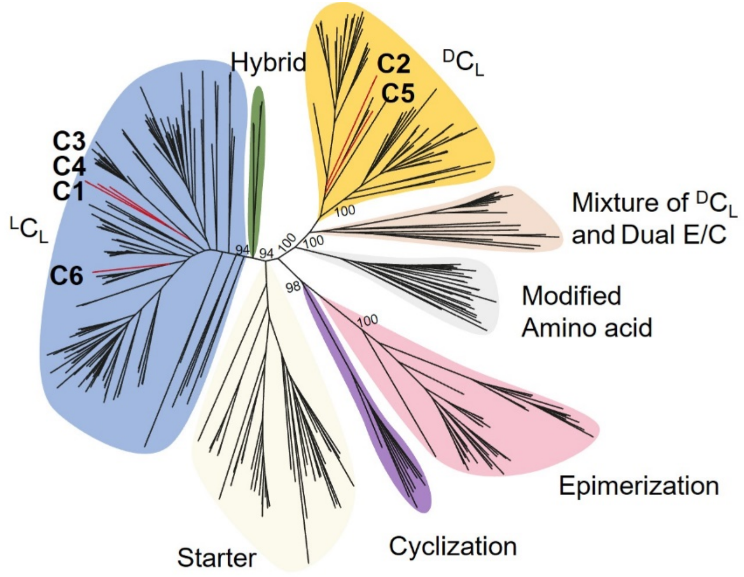


**Figure S14.** Phylogenetic analysis of the C-domains, which subdivide into the canonical clades $^LC_L$; Hybrid, occurs in PKS-NRPS biosynthetic gene clusters; $^DC_L$; Dual E/C, catalyzes both condensation and epimerization; Modified amino acids; Epimerization; Cyclization and Starter. C1–C6 show the C-domains of *P. oxalativorans* and *P. sputorum*. Bootstrap values are given for the main branches.

**Table S9.** Additional C-domains used for phylogeny analysis.

| Accession No. | name of protein | Natural product | Organism |
|---|---|---|---|
| AIZ66879.1 | IgnD | Legonmycin | *Streptomyces* sp. MA37 |
| CCM44337.1 | Zmn17 | Zeamine | *Serratia plymuthica* RVH1 |
| ABP57749.1 | DepE | FK228 | *Chromobacterium violaceum* |
| CZT62784.1 | HasO | Hassallidin | *Planktothrix serta* PCC 8927 |
| AIW82283.1 | PuwF | Puwainaphycin | *Cylindrospermum alatosporum* CCALA 988 |
| AIW82284.1 | PuwG | Puwainaphycin | *Cylindrospermum alatosporum* CCALA 988 |
| WP_003139386.1 | AmbE | AMB | *Pseudomonas aeruginosa* PAO1 |
| WP_003113143.1 | AzeB | Azabicyclene | *Pseudomonas aeruginosa* PAO1 |
| AWI62628.1 | VioC | Vioprolide | *Cystobacter violaceus* Cb vi35 |
| AVI26390.1 | TnaC | Theonellamide | *Candidatus Entotheonella serta* |
| PHM32986.1 | FclJ | Fabclavine | *Xenorhabdus szentirmaii* DSM 16338 |
| ABL74940.1 | TlmVI | Tallysomycin | *Streptoalloteichus hindustanus* E465-94 ATCC 31158 |
| ABL74936.1 | TlmX | Tallysomycin | *Streptoalloteichus hindustanus* |
| ACG60776.1 | ZbmVI | Zorbamycin | *Streptomyces pilosus* ATCC21892 |
| ACG60782.1 | ZbmX | Zorbamycin | *Streptomyces pilosus* ATCC21892 |
| CDF96614.1 | BraB | Brabantamide | *Pseudomonas* sp. SHC52 |
| WP_018674291.1 | BogB | Bogorol | *Brevibacillus laterosporus* |
| CCJ67645.1 | JagA | Jagaricin | *Janthinobacterium agaricidamnosum* |
| CCJ67647.1 | JagC | Jagaricin | *Janthinobacterium agaricidamnosum* |
| WP_150244304.1 | AlbB | Albopeptide | *Streptomyces albofaciens* |
| WP_052498266.1 | HptC | Haereoplantin | *Burkholderia plantarii* |

**Methylation and derivatization with the Mosher reagent**

For elucidation of absolute configurations of 3-hydroxy fatty acids pandorabactin A (3.0 mg to 6.0 mg) was hydrolyzed with 200 µL of 6 M HCl and stirred at 105 °C for 2.5 h. Pandorabactin B (5.0 mg) was hydrolyzed with 400 µL of 1 M $H_2SO_4$ stirred at 80 °C for 2 h. The hydrolysates were extracted with EtOAc (4×) and solvent was removed under reduced pressure. The dried products were dissolved in 0.3 mL dry MeOH. Next $TMSCHN_2$ (2.0 M in hexane) were added until the reaction turned yellow. The reactions were stirred for 5–10 min at room temperature.[14-15] Afterwards, reactions were quenched with 5 µL formic acid until color disappeared and the solvent was removed under reduced pressure. Crude methylation products were dissolved in 0.3 mL dry $CH_2Cl_2$. Next 10 µL anhydrous pyridine and a catalytic amount of DMAP was added. Further 20 µL of (*S*)-(+)-MTPA-Cl were added. The reactions were stirred for 3 h at room

temperature. After quenching the reactions with 500 µL water, the phases were separated, and the aqueous phase was extracted with $CH_2Cl_2$ (4×). Combined organic phases were dried with $Na_2SO_4$ and the solvent was removed under reduced pressure. The MTPA derivatives were analyzed using GC-MS.
Reference substances 3-OH-buryric acid, (*S*)-3-OH-butyric acid, 3-OH-hexanoic acid, (*S*)-3-OH-hexanoic acid, 3-OH-octanoic acid and (*S*)-3-OH-octanoic acid were derivatized and analyzed the same way.

For pandorabactin A (**1**) 3-OH-hexanoic acid and 3-OH-buryric acid found to be *R*-configurated. For pandorabactin B (**2**) 3-OH-octanoic acid found to be *R*-configurated. The absolute configuration of 3-OH-buryric acid could not be determined.

**Preparation of mutants of *P. oxalativorans* DSM 23570**

*P. oxalativorans* DSM 23570 genomic DNA was isolated by using Promega Wizard® DNA Purification kit (Promega GmbH). Two gene fragments were amplified by PCR with the primer pairs PoNRPS-KO1-HindIII/PoNRPS-KO1-PacI and PoNRPS-KO2-KpnI/PoNRPS-KO2-NheI using Phusion Polymerase (New England Biolabs). The obtaining amplicons were purified using the illustra GFX PCR DNA and Gel Band Purification Kit (GE Healthcare Life Sciences) and cloned into pCR bluntII vector, resulting in pCR-PoNRPS-KO1 and pCR-PoNRPS-KO2, respectively. *Pac*I/*Kpn*I restricted chloramphenicol resistance cassette gene from pGEM-Cm$^R$ [16], *Hin*dIII/*Pac*I restricted pCR-PoNRPS-KO1, and *Kpn*I/*Nhe*I restricted pCR-PoNRPS-KO2 were cloned into *Hin*dIII/*Spe*I restricted pGL42a_T251A[2], generating pGL42a_T251A-Δ*PoNRPS*. pGL42a_T251A-Δ*PoNAT* was prepared as above-mentioned procedure using the primer pairs PoNAT-KO1-HindIII/PoNAT-KO1-PacI and PoNAT-KO2-KpnI/PoNAT-KO2-NheI.

*P. oxalativorans* DSM 23570 was cultured in TSB medium (2 mL) with orbital shaking at 30 °C. Overnight cultured cells were inoculated in TSB medium (1/100 and/or 1/50 dilution) and cultured up to $OD_{600}$ = 0.4 to 0.6 with orbital shaking at 30 °C. The obtained culture broth was centrifuged at 3,000 × *g* and the supernatant was discarded. The cell pellet was resuspended in 10% glycerol (*v*/*v*) and centrifuged again. After repeating this washing step three times, the washed cells were resuspended in 10% glycerol (*v*/*v*) and subjected to electroporation (2,500 V) with mutant plasmids (*ca.* 1 µg). Transformed cells were cultured in TSB medium (1 mL) for 4 h at 30 °C with orbital shaking and then plated on nutrient agar plates with chloramphenicol (50 µg · $mL^{-1}$). After 4 days, several positive colonies were observed and then inoculated on nutrient agar plates with chloramphenicol (50 µg · $mL^{-1}$) and D,L-*p*-chlorophenylalanine (2 mg · $mL^{-1}$). These resistant mutants were cultured at 30 °C for 2 days and checked by colony PCR using the primer pairs PoNRPS-fw/PoNRPS-rv or PoNAT-fw/PoNAT-rv. Final PCR confirmation of knock-out mutants was performed using genomic DNA as a template, which was purified with The Wizard® Genomic DNA Purification Kit.

**Table S10.** The primers used in this study. Underlines show restriction sites.

| Primer | Nucleotide (5' to 3') | Source |
|---|---|---|
| PoNRPS-KO1-HindIII | GGTAAGCTTTCGTTATGCTGGCGAGACGG | This study |
| PoNRPS-KO1-PacI | GGTTTAATTAACCAGAGAGACCTCGGTGAG | This study |
| PoNRPS-KO2-KpnI | GGTGGTACCGATCAACTTCGACGGTGCGC | This study |
| PoNRPS-KO2-NheI | GGTGCTAGCAAGTTGTCGTCGCGGCTCAC | This study |
| PoNAT-KO1-HindIII | GGTAAGCTTACGCTCTCGACCGCATGCTG | This study |
| PoNAT-KO1-PacI | GGTTTAATTAATCACGAACCGCTGCGACGAG | This study |
| PoNAT-KO2-KpnI | GGTGGTACCCTTGGCGAAGATGCAGGCCG | This study |
| PoNAT-KO2-NheI | GGTGCTAGCAGAACTTCCGGCGACAACCG | This study |
| PoNRPS-fw | ATGATCGCAGGACTGCTCGG | This study |
| PoNRPS-rv | CACCGAACGAGAGCAAACGC | This study |
| PoNAT-fw | TCAGGCGTTGTTGCCGGTGC | This study |
| PoNAT-rv | AAACCTCGGTCGTAGTCGGC | This study |
| PanQ-fw-BamHI2 | GGTGGATCCACTGCCCCTGTTGAATCGTCCGC | This study |
| PanQ-rv-HindIII | GGTAAGCTTTCACCACGGCGCGTATTGCTCG | This study |

**Table S11.** Plasmids used in this study.

| Plasmid | Character | Source or reference |
|---|---|---|
| pCR bluntII | General blunt end cloning vector | Thermo Fisher Scientific |
| pGL42a-T251A | Suicide plasmid using *pheS* modified with T251A | [2] |
| pGEM-Cm$^R$ | pGEM T-easy with chloramphenicol resistance gene cassette (Cm$^R$) | [16] |
| pCR-PoNRPS-KO1 | pCR bluntII with partial NRPS gene | This study |
| pCR-PoNRPS-KO2 | pCR bluntII with partial NRPS gene | This study |
| pCR-PoNAT-KO1 | pCR bluntII with partial NAT gene | This study |
| pCR-PoNAT-KO2 | pCR bluntII with partial NAT gene | This study |
| pGL42a_T251A-ΔPoNRPS | pGL42a_T251A containing NRPS gene insertional Cm$^R$ | This study |
| pGL42a_T251A-ΔPoNAT | pGL42a_T251A containing NAT gene insertional Cm$^R$ | This study |
| pCRblunt-panQ | pCR bluntII with panQ gene | This study |
| pET28a-panQ | pET28a with panQ gene | This study |

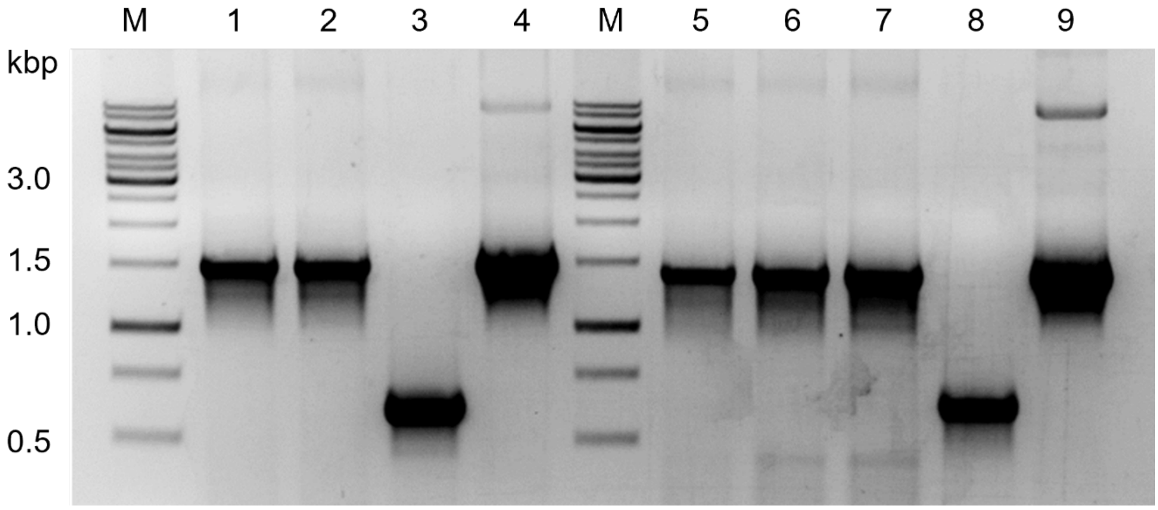


**Figure S15.** PCR confirmation of *P. oxalativorans* mutants. Template DNA: *P. oxalativorans* Δ*NRPS* (1, 2), wild type (3, 8), pGL42a_T251A-Δ*NRPS* (4), *P. oxalativorans* Δ*NAT* (5–7), pGL42a_T251A-Δ*NAT* (9). Primer pairs PoNRPS-fw/PoNRPS-rv (lanes 1−4) and PoNAT-fw/PoNAT-rv (lanes 5−9). M: marker. The estimated size of amplicons, lane 1, 2, 4; 1,485 bp, 3; 609 bp, 5–7, 9; 1,401 bp, 8; 609 bp.

### Extraction of mutants of *P. oxalativorans* DSM 23570

*P. oxalativorans* Δ*panQ and* Δ*panI* were precultured in MM9 medium supplemented with trace elements and chloramphenicol (25 µg · mL$^{-1}$), with orbital shaking at 110 × *g* and 30 °C overnight. The obtained cultures were added to 150 mL MM9 medium without trace elements and chloramphenicol (25 µg · mL$^{-1}$), which was distributed in baffled Erlenmeyer flasks and orbital shaken at 110 × *g* and 30 °C for two days, with a start $OD_{600}$ of 0.05. For extraction Amberlite® XAD16 resin was used. The resin was prepared by washing it with methanol (3x) and acetone (2x) and equilibrated with $ddH_2O$. Before extraction the cultures were centrifuged at 5,000 × *g* for 20 min at room temperature. Then, the resin was added to the supernatant, and they were shaken at 110 × *g* and 30 °C for 2 h. The resin was filtered off and eluted with methanol. Volatiles were removed under reduced pressure. The samples were dissolved in MeOH for HPLC-HRMS analysis.

### Preparation of the synthetic substrate for the complementation assay

The substrate $N^{\delta}$-Hydroxy-$N^{\delta}$-((*R*)-3-OH-butyryl)-L-ornithine for the complementation assay was synthesized according to the procedures of Heemstra *et. al.*[17] and Williams *et. al.*[18]

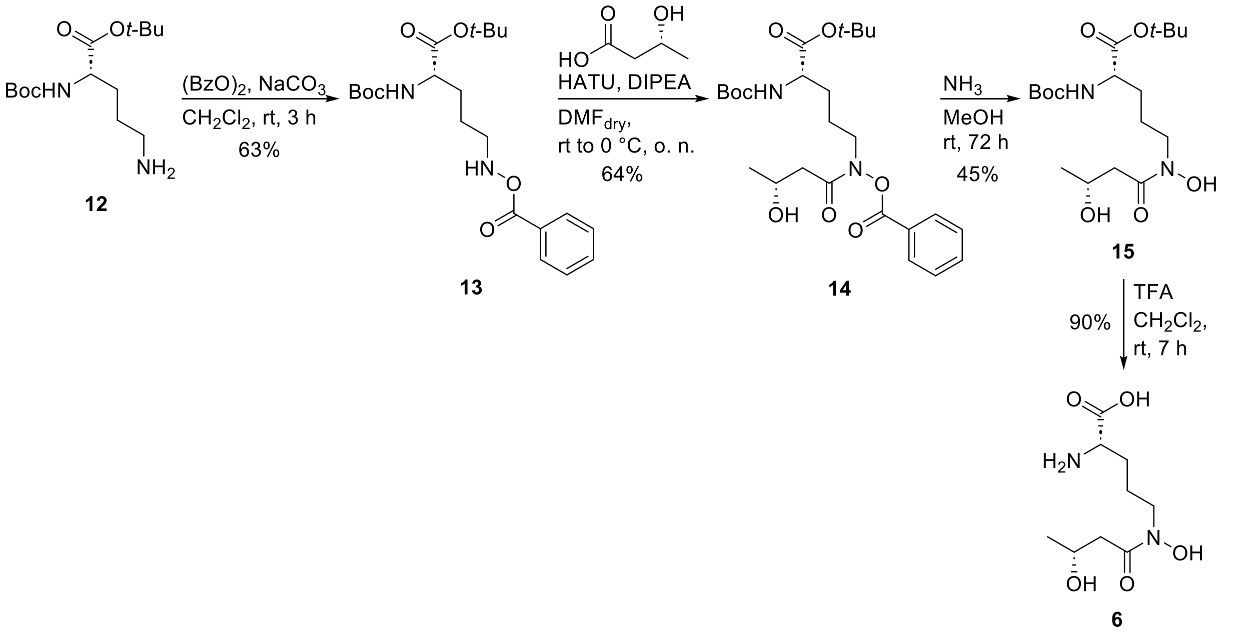

**Scheme S1:** Synthesis of $N^{\delta}$-Hydroxy-$N^{\delta}$-((*R*)-3-OH-butyryl)-L-ornithine.

### Synthesis of $N^{\delta}$-benzoyloxy-$N^{\alpha}$-Boc-L-ornithine *tert*-butyl ester (13)

$N^{\alpha}$-Boc-L-ornithine *tert*-butyl ester (**12**) (1.10 g, 3.81 mmol, 1.0 eq.) was dissolved in sat. aq. sodium carbonate (17.6 mL). Separately, benzoyl peroxide (with 25% $H_2O$) (1.85 g, 7.63 mmol, 2.0 eq.) was suspended in $CH_2Cl_2$ (17.6 mL) and added dropwise to the reaction with rapid stirring. The reaction mixture was stirred for 3 h at room temperature and then diluted with $H_2O$ (25 mL). The reaction mixture was extracted with $CH_2Cl_2$ (3×). The organic layers were combined, dried over $Na_2SO_4$, filtered and dried under reduced pressure. The white residue was purified by column chromatography (Cyclohexane:EtOAc 4:1) to afford the product (0.97 g, 2.38 mmol, 63%) as a white solid.

$\boldsymbol{R_f}$(Cyclohexane:EtOAc 4:1): 0.26.
$[\alpha]_D^{22.8}$ = –15.84 (c = 1.40 in MeOH)
**$^1$H NMR** ($CDCl_3$, 500 MHz): $\delta$ [ppm] 8.01 (dd, *J* = 8.4, 1.2 Hz, 2 H), 7.59 (t, *J* = 7.5 Hz, 1 H), 7.46 (t, *J* = 7.6 Hz, 2 H), 5.10 (br s, 1 H), 4.21 (br s, 1 H), 3.17 (m, 2 H), 1.93 (m, 1 H), 1.73 (m, 3 H), 1.45 (s, 9 H,), 1.43 (s, 9 H).
**$^{13}$C NMR** ($CDCl_3$, 125 MHz): $\delta$ [ppm] 171.8 (q), 166.9 (q), 155.5 (q), 133.6 (t), 129.5 (t), 128.7 (t), 128.3 (q), 82.2 (q), 79.9 (q), 53.8 (t), 52.2 (s), 30.7 (s), 28.5 (p), 28.1 (p), 23.2 (s).
**HRMS (ESI$^+$):** $[M+H]^+$ *m/z* calcd. for $C_{21}H_{33}N_2O_6^+$: 409.2333; found: 409.2331.

### Synthesis of $N^{\delta}$-benzoyloxy-$N^{\delta}$-(*R*)-3-hydroxy-butyryl-$N^{\alpha}$-Boc-L-ornithine *tert*-butyl ester (14)

(*R*)-3-Hydroxy-butyric acid (48.9 mg, 0.47 mmol, 1.0 eq) was dissolved in $DMF_{dry}$ (0.97 mL) and HATU (357.4 mg, 0.94 mmol, 2.0 eq) was added at 0 °C. After 30 min, a solution of $N^{\delta}$-benzoyloxy-$N^{\alpha}$-Boc-L-ornithine *tert*-butyl ester (**13**) (200.0 mg, 0.49 mmol, 1.05 eq.) in $DMF_{dry}$ (0.42 mL) was added. DIPEA (104 µL, 0.61 mmol, 1.3 eq) was added dropwise to the reaction mixture at 0 °C. The reaction was warmed to room temperature and stirred overnight. The reaction mixture was extracted with $CH_2Cl_2$ (3×) and washed with brine. The combined organic layers were dried over $Na_2SO_4$, filtered and dried under reduced pressure. The residue was purified by RP-HPLC (solvents: 83% acetonitrile/$H_2O$, gradient: 0–30 min 50%→100% acetonitrile, hold 6 min at 100% acetonitrile) to give the product (148.1 mg, 0.30 mmol, 64%) as a colorless oil.

$\boldsymbol{t_R}$ = 13.3 min
$[\alpha]_D^{23.4}$ = –11.96 (c = 0.72 in MeOH)
**$^1$H NMR** ($CDCl_3$, 500 MHz): $\delta$ [ppm] 8.08 (d, *J* = 7.5 Hz, 2 H), 7.69 (t, *J* = 6.9 Hz, 1 H), 7.52 (t, *J* = 7.4 Hz, 2 H), 5.10 (d, *J* = 7.9 Hz, 1 H), 4.23 (m, 1 H), 4.18 (m, 1 H), 3.83 (m, 2 H), 2.39 (m, 2 H), 1.87 (m, 1 H), 1.68 (m, 3 H), 1.43 (s, 9 H,), 1.40 (s, 9 H), 1.17 (s, 3 H).
**$^{13}$C NMR** ($CDCl_3$, 125 MHz): $\delta$ [ppm] 173.3 (q), 171.7 (q), 164.4 (q), 155.5 (q), 134.9 (t), 130.2 (t), 129.1 (t), 126.4 (q), 82.2 (q), 79.8 (q), 64.1 (t), 53.6 (t), 47.5 (s), 40.1 (s), 30.3 (s), 28.4 (p), 28.1 (p), 23.0 (s). 22.3 (p).
**HRMS (ESI$^+$):** $[M+H]^+$ *m/z* calcd. for $C_{25}H_{39}N_2O_8^+$: 495.2701; found: 495.2694.

### Synthesis of $N^{\delta}$-hydroxy-$N^{\delta}$-(*R*)-3-hydroxy-butyryl$N^{\alpha}$-Boc-L-ornithine *tert*-butyl ester (15)

$N^{\delta}$-Benzoyloxy-$N^{\delta}$-(*R*)-3-hydroxy-butyryl-$N^{\alpha}$-Boc-L-ornithine *tert*-butyl ester (**14**) (111.4 mg, 0.23 mmol, 1.0 eq) was dissolved in $NH_3$ in MeOH (7.7 mL, 2 M). The reaction mixture was stirred for 72 h at room temperature. The solvent was removed under reduced pressure and the residue was purified by RP-HPLC (solvents: 83% acetonitrile/$H_2O$, gradient: 0–30 min 50%→100% acetonitrile, hold 6 min at

100% acetonitrile) to afford the product (40.8 mg, 0.10 mmol, 45%) as a light-yellow oil. A side reaction was detected in which the benzoyloxy protection group was shifted to the hydroxy function of the butyryl moiety.
The NMR data showed a complex mixture of rotomers, so the product was used without full NMR characterization.

**$t_R$** = 7.2 min
**HRMS (ESI⁺):** $[M+H]^+$ *m/z* calcd. for $C_{18}H_{35}N_2O_7^+$: 391.2439; found: 391.2436.

**Synthesis of $N^\delta$-hydroxy-$N^\delta$-(*R*)-3-hydroxy-butyryl-L-ornithine (6)**

$N^\delta$-Hydroxy-$N^\delta$-(*R*)-3-hydroxy-butyryl-$N^\alpha$-Boc-L-ornithine *tert*-butyl ester (**15**) (40.8 mg, 0.10 mmol, 1.0 eq) was dissolved in $CH_2Cl_2$ (0.5 mL). TFA (0.5 mL) was then added dropwise, and the reaction mixture was stirred at room temperature for 7 h. The solvent was concentrated under reduced pressure. $H_2O$ was added to the residue and the solution was extracted with $CH_2Cl_2$ (3×). The combined aqueous layers were freeze-dried to afford the product (22.0 mg, 0.09 mmol, 90%) as a white powder.

**$[\alpha]_D^{23.7}$** = –1.71 (c = 0.70 in MeOH)
**$^1$H NMR** ($D_2O$, 500 MHz): $\delta$ [ppm] 4.20 (m, 1 H), 3.85 (t, *J* = 6.2 Hz 1 H), 3.66 (t, *J* = 6.6 Hz 2 H), 2.76 (dd, *J* = 14.8, 8.2 Hz 1 H), 2.57 (dd, *J* = 14.8, 5.1 Hz 1 H), 1.86 (m, 2 H), 1.72 (m, 2 H), 1.20 (d, *J* = 6.2, 3 H).
**$^{13}$C NMR** ($D_2O$ 125 MHz): $\delta$ [ppm] 174.1 (q), 173.8 (q), 65.3 (t), 54.1 (t), 47.9 (s), 41.3 (s), 27.9 (s), 22.7 (p). 22.4 (s).
**HRMS (ESI⁺):** $[M+H]^+$ *m/z* calcd. for $C_9H_{19}N_2O_5^+$: 235.1288; found: 235.1287.

**Chemical complementation of *P. oxalativorans* Δ*panQ***

The bacteria were precultured in 2 mL iron-deficient MM9 medium with orbital shaking at 120 × *g* and 30 °C overnight. The obtained cultures were added to 1.9 mL (final volume) iron-deficient MM9 medium with a starting $OD_{600}$ of 0.05 with orbital shaking at 120 × *g* and 30 °C for 3 days. To supplement the cultures of the mutant strain, a solution of the synthetic $N^\delta$-Hydroxy- $N^\delta$-(*R*)-3-hydroxy-butyryl-L-ornithine (**13**) in dd$H_2O$ was prepared (2 mM). The solution was sterilized by filtration using 33 mm Ezee Syringe Filters (0.22 µm, PVDF) from Elkay. 100 µL of the substrate was added to the bacterial culture (100 µM final concentration). For the negative control 100 µL of sterile dd$H_2O$ were added to the mutant culture. *P. oxalativorans* wild-type was used as positive control. For extraction Amberlite® XAD16 resin was used. The resin was prepared by washing it with methanol (3x) and acetone (2x) and equilibrated with dd$H_2O$. Before extraction the cultures were centrifuged at 5,000 × *g* for 10 min at room temperature. Then, the resin was added to the supernatant, and they were shaken at 120 × *g* at room temperature for 1 h. The resin was filtered off and eluted with MeOH. The solvent was removed under reduced pressure and the samples were dissolved in MeOH for HPLC-HRMS analysis. The assay was performed in triplicates. The extracts were also analyzed by HPLC-MS, AmaZon Speed (Bruker Daltonics GmbH & Co.) ion trap mass spectrometry coupled with an Agilent Technologies 1260 Infinity series liquid chromatogram system. The reversed-phase HPLC column was used Phenomenex Hydro-RP, diameter 4 µm, pore size 80 Å, 4.6 × 250 mm (Agilent technologies) at a flow-rate at 0.8 mL $min^{-1}$, using a gradient system; solvent A (dd$H_2O$ containing 0.1% formic acid), solvent B (100% acetonitrile), 0% B for 5 min to 40% B in 15 min.

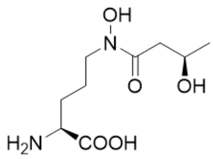


**6**

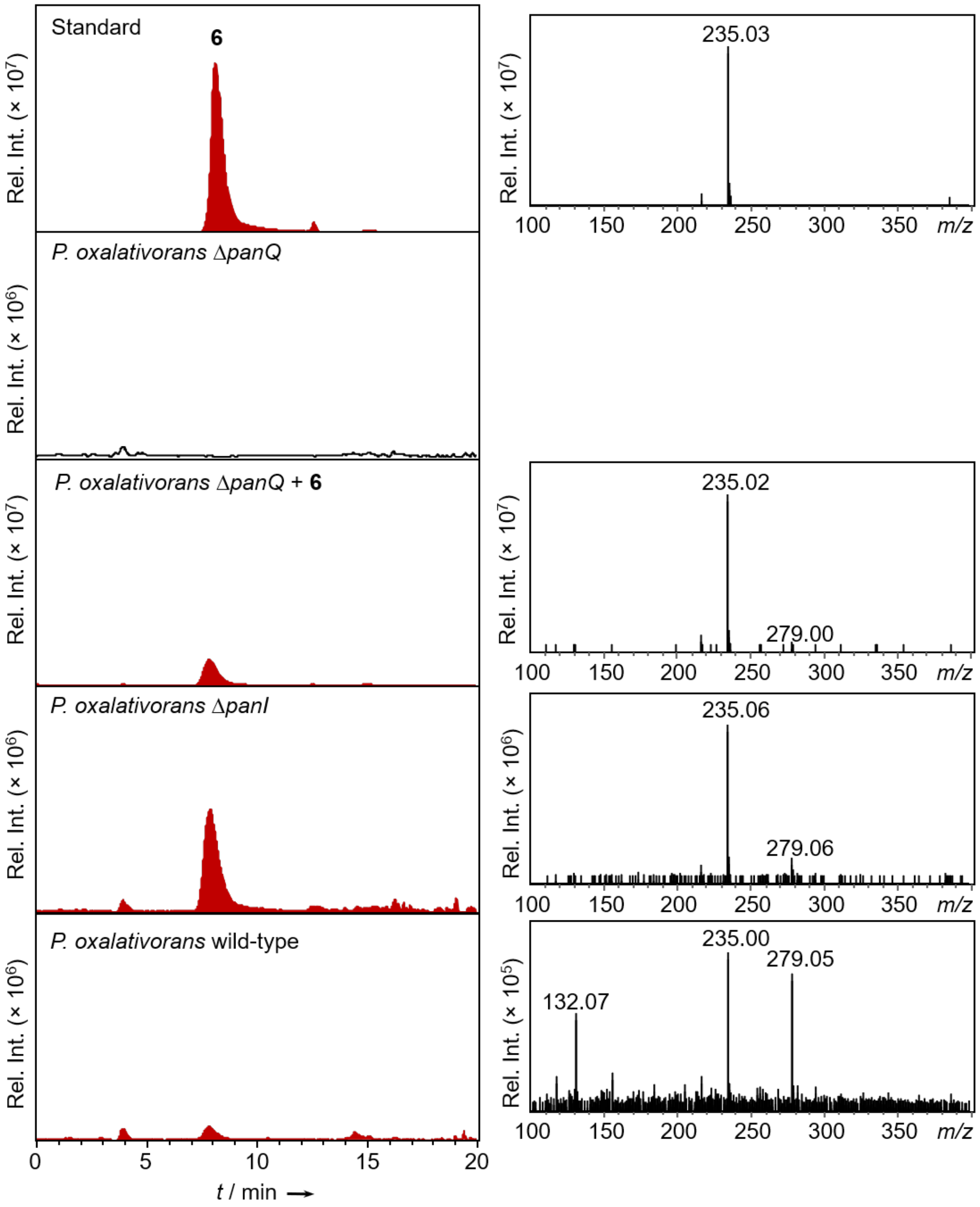


**Figure S16.** HPLC-ESIMS (EIC, Extracted Ion Chromatogram, *m/z* 235.05 ± 0.2 ppm) profiles of the chemical substrate $N^{\delta}$-hydroxy-$N^{\delta}$-(*R*)-3-hydroxy-butyryl-L-ornithine (**6**), the *P. oxalativorans* Δ*panQ*, *P. oxalativorans* Δ*panI*, and the *P. oxalativorans* wild-type. Left panel indicates EIC profiles. Right panel shows observed ions of **6**.

### Construction of panQ heterologous expression plasmid

*P. oxalativorans* DSM 23570 genomic DNA was isolated by using Promega Wizard® DNA Purification kit (Promega GmbH). A panQ gene was amplified by PCR with the primer pair panQ-fw-BamHI2/panQ-rv-HindIII using Phusion Polymerase. The obtaining amplicon was purified using the illustra GFX PCR DNA and Gel Band Purification Kit and cloned into pCR bluntII vector, resulting in pCRblunt-panQ. *Bam*HI/*Hind*III restricted pCRblunt-panQ was cloned into *Bam*HI/*Hind*III restricted pET28a vector, generating pET28a-panQ.

**Heterologous produciton and purification of PanQ**

*E. coli* BL21 (DE3) pET28a-panQ was cultured in 2 mL LB medium with kanamycin (50 µg $mL^{-1}$) at 37 °C for overnight. One mL of pre-culture was inoculated into 100 mL LB medium (2 × 300 mL buffled Erlenmeyer flask) with kanamycin (50 µg $mL^{-1}$) and cultivated with orbital shaking at 37 °C for 2–3 h until the $OD_{600}$ reached 0.5–0.6. This culture was cooled down on an ice for 15 min and then 0.2 mM IPTG was added to the culture medium. This culture was further incubated with orbital shaking at 16 °C for 16 h. The cells were harvested by centrifugation and stored at −20 °C.
Frozen cells from 2 × 100 mL cultured cells were suspended in 50 mM Tris HCl (pH 8.0) buffer with 300 mM NaCl, 5% glycerol (*w*/*v*), 500 mM L-arginine, 10 mM imidazole, 1 mg $mL^{-1}$ lysozyme (Carl Roth), and 10 µL DNase I (NEB). After this solution was incubated at 37 °C for 30 min, this solution was sonicated by Sonoplus HD4100 (Bandelin, 90% power, 5 cycles for 2 min, repeated at 3 times) on an ice. After the centrifugation of destroyed cells at 10,000 × *g* for 30 min at 4 °C, the supernatant was filtered by Chromafil (0.45 µm, Macherey-Nagel) and subjected to a Ni-NTA column (2 mL, Quiagen) and washed with 50 mL of 50 mM Tris HCl (pH 8.0) buffer with 300 mM NaCl, 5% glycerol (*w*/*v*), and 10 mM imidazole. His6-PanQ was eluted with 10 mL of 50 mM Tris HCl (pH 8.0) buffer with 300 mM NaCl, 5% glycerol (*w*/*v*), and 500 mM imidazole. This eluent was concentrated by Amicon Ultra centrifugal filter 30 kDa (Merck Millipore) to obtain 9.5 mg (calculated from an absorbance at 280 nm) of $His_6$-PanQ. This solution was adjusted as 50 mM HEPES pH 7.5, 300 mM NaCl, and 40% glycerol and stored at −20 °C.

**Preparation of $N^{\delta}$-hydroxy-L-ornithine (5) and 3-(*R*)-Hydroxy-butyryl-CoA (7)**

$N^{\delta}$-OH-L-ornithine (**5**) was prepared by the method reported by Stattely and coworkers.[17] Preparation of 3-(*R*)-hydroxy-butyryl-CoA (**7**) is prepared as follows. To 5.2 mg (50 µmol) of 3-(*R*)-hydroxy-butyric acid in dry DMF (3 mL), *N*,*N*-diisopropylethylamine (10 µL, 60 µmol), and was added under argon. To this mixture, HBTU (2-(1H-benzotriazol-1-yl)-1,1,3,3-tetramethyluronium-hexafluorophosphate, 23 mg, 60 µmol), HOBt (1-hydroxybenzotriazole, 8 mg, 60 µmol), and Coenzyme A (38 mg, 50 µmol) were added and further stirred at room temperature for 16 h. The reaction mixture was stirred under argon at room temperature for 2 h. After the solvent was removed under the reduced pressure, the residue was dissolved in $H_2O$ and then subjected to the reversed-phase HPLC (Nucleodur HTec C18 diameter 5 µm, pore size 100 Å, 21.2 × 250 mm, Macherey Nagel) at a flow-rate of 12 mL $min^{-1}$ using a gradient system; solvent A (dd$H_2O$ containing 0.1% TFA), solvent B (83% acetonitrile), 1% B for 5 min to 100% B in 35 min to yield 3-(*R*)-hydroxy-butyryl-CoA (**7**, 21.2 mg, 29 µmol) as white solid.

**$^1$H NMR** (DMSO-$d_6$, 600 MHz): δ [ppm] 8.65 (s, 1H), 8.39 (s, 1H), 8.12 (t, *J* = 5.6 Hz, 1H), 7.74 (t, *J* = 5.5 Hz, 1H), 5.98 (d, *J* = 5.2 Hz, 1H), 4.79 (m, 1H), 4.71 (t, *J* = 5.2 Hz, 1H), 4.41 (s, 1H), 4.14–4.25 (m, 2H), 4.02 (m, 1H), 3.90 (dd, *J* = 9.2, 5.3 Hz, 1H), 3.74 (s, 1H), 3.57 (m, 2H), 3.31 (m, 1H), 3.22 (m, 1H), 3.15 (m, 2H), 2.88 (m, 2H), 2.64 (dd, *J* = 14.4, 7.4 Hz, 1H), 2.58 (dd, *J* = 14.4, 5.5 Hz, 1H), 2.25 (t, J = 7.2 Hz, 2H), 1.08 (d, *J* = 6.3 Hz, 3H), 0.93 (s, 3H), 0.75 (s, 3H).
**HRMS (ESI$^+$):** $[M+H]^+$ *m/z* calcd. for $C_{25}H_{43}N_7O_{18}P_3S^+$: 854.1593; found: 854.1593.

***In vitro* reconstitutiuon of PanQ activity**

The *in vitro* $N^{\delta}$-hydroxyornitine ($N^{\delta}$-OHOrn) $N^{\delta}$-3-(*R*)-hydroxybutyryl transfer activity assay was carried out in analogy to the reported procedure.[17] The 50 µL reaction mixture consisted of 2.5 µL of $His_6$-PanQ (200 µM in 50 mM HEPES pH7.5, 300 mM NaCl, 40 % glycerol), 5 µL of $N^{\delta}$-OHOrn (10 mM in $H_2O$), 5 µL of 3-(*R*)-hydroxybutyryl-CoA (10 mM in 50 mM Tris-HCl, pH 7.5 ), 2.5 µL of 1 M Tris-HCl pH 7.5, 35 µL of $H_2O$. This mixture was incubated at 30 °C for 10 min and then the reaction as stopped by the addition of 5 µL of formic acid. This enzyme mixture was cooled at –20 °C for 30 min and then centrifuged to remove enzymes. The supernatant was analyzed by HPLC-MS, AmaZon Speed (Bruker Daltonics GmbH & Co.) ion trap mass spectrometry coupled with an Agilent Technologies 1260 Infinity series liquid chromatogram system. The reversed-phase HPLC column was used Eclipse plus C18, diameter 3.5 µm, pore size 95 Å, 4.6 × 100 mm (Agilent technologies) at a flow-rate at 1 mL $min^{-1}$, using a gradient system; solvent A (dd$H_2O$ containing 0.1% formic acid), solvent B (100% acetonitrile), 1% B in 2 min to 50% B in 15 min.

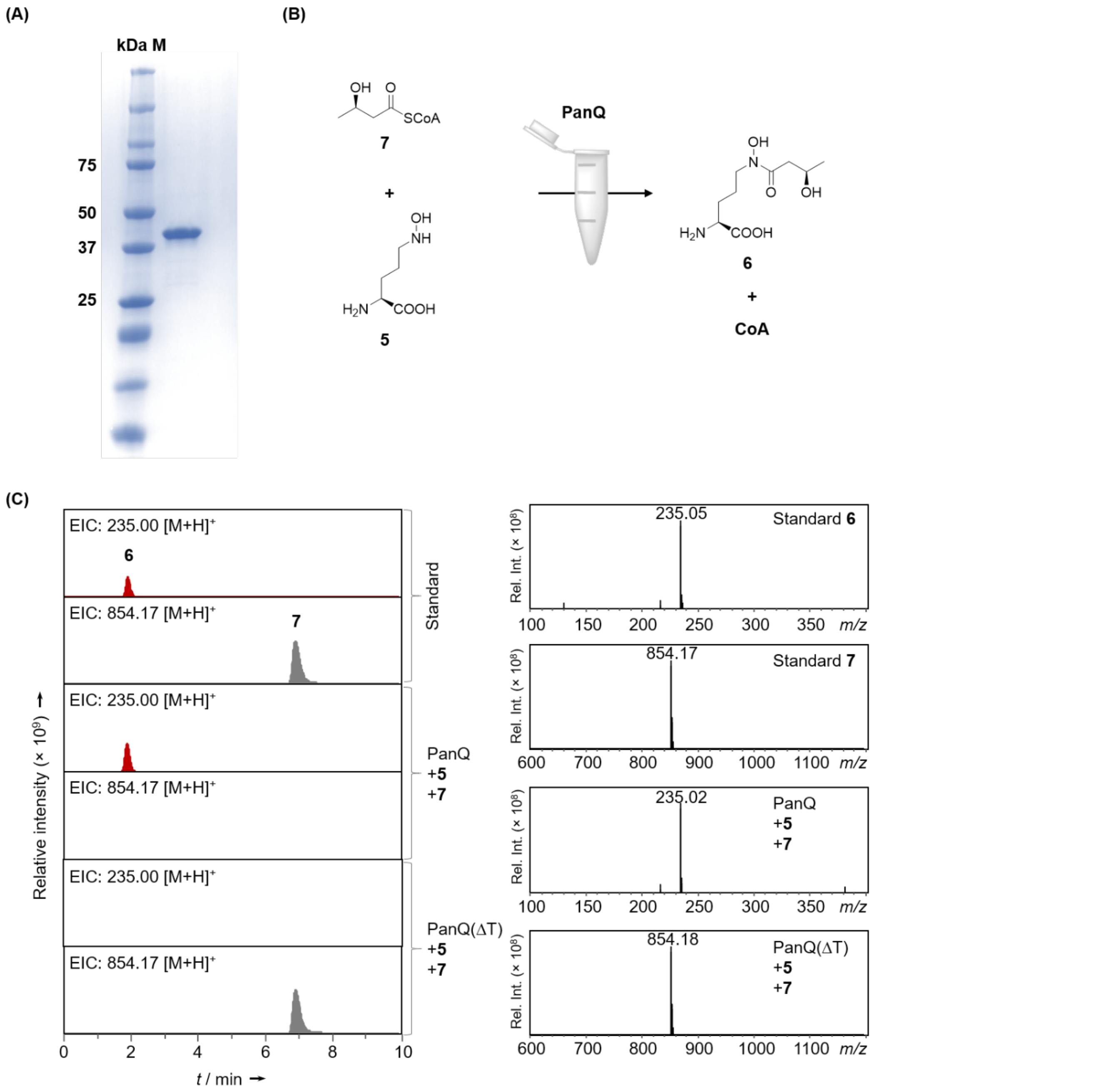


**Figure S17.** Purified $His_6$-PanQ and in vitro reconstitution of CoA-dependent $N^{\delta}$-hydroxy-L-ornithine (**5**) *N*-((*R*)-3-hydroxy-butryl)-transferase activity. (A) SDS-PAGE (4–20% gradient gel) of $His_6$-PanQ (44.98 kDa), which is purified by Ni-NTA resin. M: Molecular marker. (B) A schematic representation of in vitro reconstitution of PanQ function which transfers *N*-((*R*)-3-hydroxy-butryl) group of CoA thioester to $N^{\delta}$ of $N^{\delta}$-hydroxy-L-ornithine. (C) HPLC-ESIMS (EIC, Extracted Ion Chromatogram, *m/z* 235.05 ± 0.5 ppm) profiles of in vitro activity assay of PanQ. The left panel indicates EIC profiles. The right panel shows observed ions of **6** and **7**.

### CAS assay solution preparation

The chrome azurol S (CAS) assay was used to analyze the iron-binding capabilities of pandorabactin A (**1**) and B (**2**). The CAS solution was prepared according to literature.[19] The compounds were dissolved in $ddH_2O$ with a concentration of 15 µM. EDTA solution was used as a positive control with a concentration of 15 µM. As a negative control $ddH_2O$ was used. 250 µL of the compounds and control solutions were mixed with 250 µL of the CAS solution.

### CAS agar preparation

1 L of CAS agar was prepared according to literature[20]: In order to obtain the CAS reagent solution, a solution 1 (60.5 mg of chrome azurol S dissolved in 50 mL $ddH_2O$ were mixed with 10 mL of 1 mM $FeCl_3$ solution in 10 mM HCl) was prepared. Under stirring a solution 1 was slowly added to solution 2 (72.9 mg of HDTMA (hexadecyltrimethylammonium bromide) dissolved in 40 mL $ddH_2O$), and the resulting dark blue solution was autoclaved. The obtained solution was cooled down to ca. 50 °C and slowly added to 900 mL of the

autoclaved MM9 medium pH 6.8 (without trace elements) with 30.2 g 1,4-piperazinediethanesulfonic acid (PIPES) and 15 g · $L^{-1}$ agar. To this solution, 16.7 mL of L-Leu (100 mM), 5 mL of L-His (60 mM), 10 mL of L-Lys (100 mM), 10 mL of L-Trp (40 mM), 10 mL of L-Met (40 mM), and 20 mL of glucose (50% (*w/v*)) were added. *P. oxalativorans* wild-type, *P. oxalativorans* Δ*panI* and *P. oxalativorans* Δ*panQ* were inoculated from NAG (nutrient agar (Roth) + 1% glycerol) agar plates and then cultured at 30 °C for 5 days.

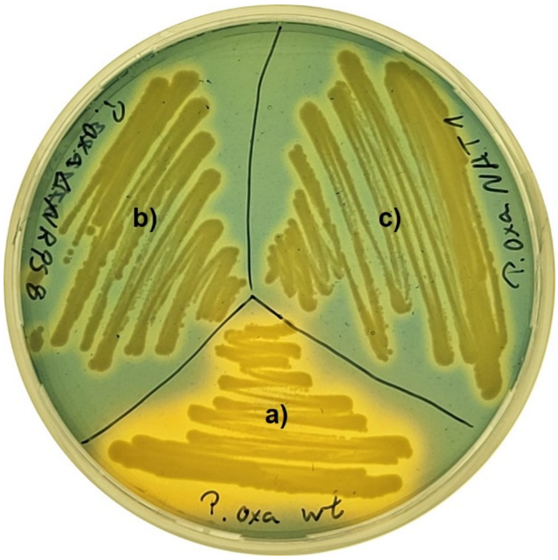


**Figure S18.** CAS agar plate with cultivated **a)** *P. oxalativorans* wild-type, **b)** *P. oxalativorans* Δ*panI and* **c)** *P. oxalativorans* Δ*panQ*.

### Preparation of $Fe^{3+}$-pandorabactin A (3)

2.8 mg (3.07 µmol, 1.0 eq) of pandorabactin A (**1**) and 3.60 mg $FeCl_3 \cdot 6\,H_2O$ (13.3 µmol, 4.3 eq) were dissolved in 0.5 mL $H_2O$. After adding 9.2 µL of a 1 M NaOH solution (9.21 µmol, 3.0 eq) the reaction mixture was stirred at room temperature for 2 h. The crude product was purified by preparative RP-HPLC using a Phenomenex Synergi 4u Fusion RP (250 × 10 mm, 4 µm, 80 Å) and gradient elution (solvent A: $H_2O$ + 0.01% TFA, solvent B: 83% acetonitrile, time program: 3 min 5% B, 5% B to 25% B in 20 min, 25% B to 100% B in 2 min and 10 min 100% B, flow rate 5 mL · $min^{-1}$), $t_R$ = 15.3 min. The complex (**3**) was obtained as a yellow/orange powder with a yield of 2.0 mg.

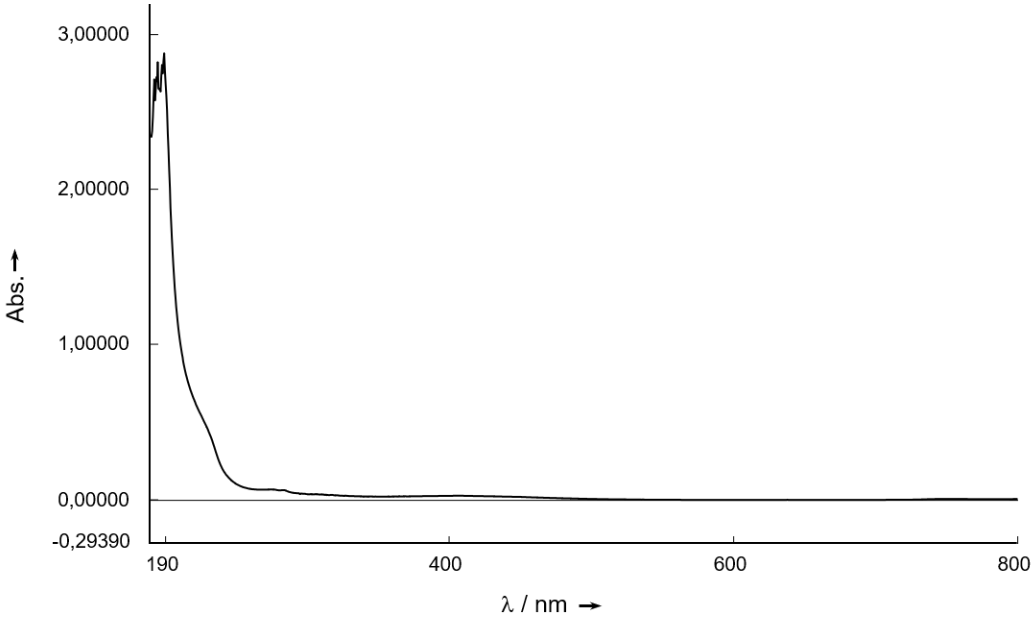


**Figure S19.** UV-Vis spectrum of $Fe^{3+}$-pandorabactin A (**3**). Spectrum was measured in MeOH with a concentration of 22.5 µg/mL.

### Preparation of $Fe^{3+}$-pandorabactin B (4)

2.0 mg (2.17 µmol, 1.0 eq) of pandorabactin B (**2**) and 2.0 mg $FeCl_3 \cdot 6\,H_2O$ (7.40 µmol, 3.4 eq) were dissolved in 0.5 mL $H_2O$. After adding 6.5 µL of a 1 M NaOH solution (6.51 µmol, 3.0 eq) the reaction mixture was stirred at room temperature for 2 h. The crude product was purified by preparative RP-HPLC using a Phenomenex Synergi 4u Fusion RP (250 × 10 mm, 4 µm, 80 Å) and gradient

elution (solvent A: $H_2O$ + 0.01% TFA, solvent B: 83% acetonitrile, time program: 3 min 10% B, 10% B to 25% B in 20 min, 25% B to 100% B in 2 min and 10 min 100% B, flow rate 5 mL · min$^{-1}$), $t_R$ = 19.3 min. The complex (**4**) was obtained as an orange/brown powder with a yield of 1.7 mg.

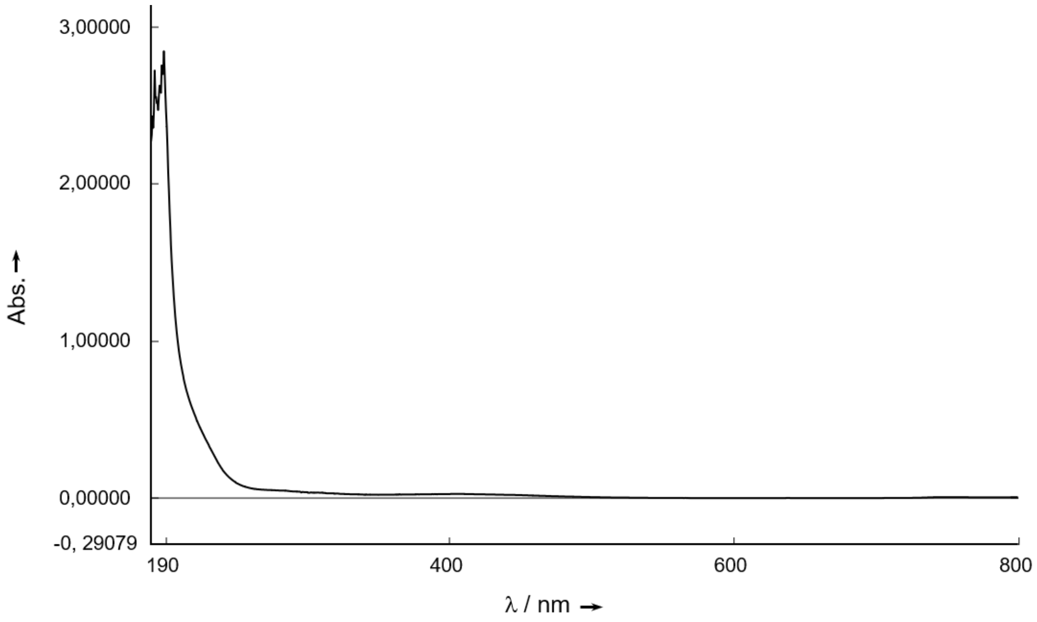


**Figure S20.** UV-Vis spectrum of $Fe^{3+}$-pandorabactin B (**4**). Spectrum was measured in MeOH with a concentration of 20.0 µg/mL.

**Preparation of $Ga^{3+}$-pandorabactin A (8)**

2.0 mg (2.23 µmol, 1.0 eq) of pandorabactin A (**1**) and 2.86 mg $Ga(NO_3)_3 \cdot xH_2O$ were dissolved in 0.5 mL MeOH. After adding 6.7 µL of a 1 M NaOH solution (6.69 µmol, 3.0 eq) the reaction mixture was stirred at room temperature for 2 h. Volatiles were removed under reduced pressure and the crude product was purified by preparative RP-HPLC using a Phenomenex Synergi 4u Fusion RP (250 × 10 mm, 4 µm, 80 Å) and gradient elution (solvent A: $H_2O$ + 0.01% TFA, solvent B: acetonitrile, time program: 3 min 2% B, 2% B to 40% B in 20 min, 40% B to 100% B in 3 min and 6 min 100% B, flow rate 5 mL · min$^{-1}$), $t_R$ = 15.1 min. The complex (**8**) was obtained as a white powder with a yield of 2.1 mg.

**Table S12.** $^{1}H$- and $^{13}C$-NMR data of $Ga^{3+}$-pandorabactin A (**8**) (600 MHz, DMSO-$d_6$).

| Partial structure | Position | $\delta_C$ [ppm] (m) | $\delta_H$ [ppm] (m, *J* [Hz]) |
|---|---|---|---|
| 3-OH-Hexanoic acid | CO | 161.7 (C) | - |
| | $C_\alpha$ | 33.6 ($CH_2$) | 2.86 (1H, dd, 15.4, 11.3), 2.64 (1H, dd, 15.3, 4.7) |
| | $C_\beta$ | 69.9 (CH) | 5.31 (1H, m) |
| | $C_\gamma$ | 36.0 ($CH_2$) | 1.43 (2H, m) |
| | $C_\delta$ | 17.5 ($CH_2$) | 1.18 (2H, m) |
| | $C_\eta$ | 13.7 ($CH_3$) | 0.84 (3H, t, 7.4) |
| $N^{\delta}$-3-OH-Butyryl-$N^{\delta}$-OH-D-ornithine | NH | - | 8.18 (1H, d, 4.1) |
| | CO | 171.1 (C)* | - |
| | $C_\alpha$ | 50.6 (CH) | 4.22 (1H, m) |
| | $C_\beta$ | 28.0 ($CH_2$) | 1.85 (1H, m), 1.63 (1H, m) |
| | $C_\gamma$ | 21.3 ($CH_2$) | 1.78 (2H, m) |
| | $C_\delta$ | 49.4 ($CH_2$) | 4.00 (1H, m), 3.46 (1H, m) |
| | CO | 161.4 (C) | - |
| | $C'_\alpha$ | 38.8 ($CH_2$) | 2.58 (1H, dd 14.1, 7.0), 2.42 (1H, dd 14.1, 5.7) |
| | $C'_\beta$ | 64.1 (CH) | 3.95 (1H, m) |
| | $C'_\gamma$ | 23.3 ($CH_3$) | 1.11 (3H, d, 6.2) |
| L-*erythro*-3-OH-Aspartate 1 | NH | - | 9.01 (1H, d, 8.4) |
| | CO | 167.3 (C) | - |
| | $C_\alpha$ | 55.0 (CH) | 5.30 (1H, m) |
| | $C_\beta$ | 74.5 (CH) | 4.39 (1H, s) |
| | COOH | 176.4 (C) | - |
| L-Threonine | NH | - | 7.31 (1H, d, 8.6) |
| | CO | 170.3 (C) | - |
| | $C_\alpha$ | 58.3 (CH) | 4.15 (1H, dd, 8.5, 0.9) |
| | $C_\beta$ | 65.1 (CH) | 4.43 (1H, m) |
| | $C_\gamma$ | 20.4 ($CH_3$) | 1.07 (3H, d, 6.5) |
| $N^{\delta}$-OH-D-Ornithine | NH | - | 8.29 (1H, br. s) |
| | CO | 167.9 (C) | - |
| | $C_\alpha$ | 53.3 (CH | 4.43 (1H, m) |
| | $C_\beta$ | 29.0 ($CH_2$) | 1.94 (1H, m), 1.70 (1H, m) |
| | $C_\gamma$ | 22.6 ($CH_2$) | 1.85 (1H, m), 1.55 (1H, m) |
| | $C_\delta$ | 50.0 ($CH_2$) | 4.00 (1H, m), 3.28 (1H, m) |
| L-Alanine | NH | - | 7.31 (1H, d, 8.6) |
| | CO | 171.5 (C) | - |
| | $C_\alpha$ | 48.3 (CH) | 4.29 (1H, m) |
| | $C_\beta$ | 16.8 ($CH_3$) | 1.31 (3H, d, 7.3) |
| L-*erythro*-3-OH-Aspartate 2 | NH | - | 6.99 (1H, d, 8.0) |
| | CO | 167.7 (C) | - |
| | $C_\alpha$ | 55.9 (CH) | 4.33 (1H, dd, 8.1, 3.3) |
| | $C_\beta$ | 73.3 (CH) | 3.83 (1H, d, 3.1) |
| | COOH | 174.6 (C)# | - |

*: used remaining carbon, #: Signal is not observed in $^{13}C$ NMR spectrum but assigned by HMBC.

**Preparation of $Ga^{3+}$-pandorabactin B (9)**

8.5 mg (9.21 µmol, 1.0 eq) of pandorabactin B (**2**) and 23.6 mg $Ga(NO_3)_3 \cdot xH_2O$ were dissolved in 1.0 mL MeOH. After adding 27.6 µL of a 1 M NaOH solution (27.6 µmol, 3.0 eq) the reaction mixture was stirred for 1 h at room temperature. Volatiles were removed under reduced pressure and the crude product was purified by preparative RP-HPLC using a Phenomenex Synergi 4u Fusion RP (250 × 10 mm, 4 µm, 80 Å) and gradient elution (solvent A: $H_2O$ + 0.01% TFA, solvent B: acetonitrile, gradient: 3 min 2% B, 2% B to 40% B in 20 min, 40% B to 100% B in 3 min and 6 min 100% B, flow rate 5 mL · min$^{-1}$), $t_R$ = 19.4 min. The complex (**9**) was obtained as a white powder with a yield of 3.2 mg.

**Table S13.** $^{1}$H- and $^{13}$C-NMR data of $Ga^{3+}$-pandorabactin B (**9**) (600 MHz, DMSO-$d_6$).

| Partial structure | Position | $\delta_C$ [ppm] (m) | $\delta_H$ [ppm] (m, *J* [Hz]) |
|---|---|---|---|
| 3-OH-Octanoic acid | CO | 161.7 (C) | - |
| | $C_\alpha$ | 33.8 ($CH_2$) | 2.85 (1H, m), 2.64 (1H, m) |
| | $C_\beta$ | 70.0 (CH) | 5.28 (1H, m) |
| | $C_\gamma$ | 33.7 ($CH_2$) | 1.43 (2H, m) |
| | $C_\delta$ | 23.8 ($CH_2$) | 1.18 (2H, m) |
| | $C_\varepsilon$ | 31.0 ($CH_2$) | 1.18 (2H, m) |
| | $C_\zeta$ | 21.9 ($CH_2$) | 1.24 (2H, m) |
| | $C_\eta$ | 13.9 ($CH_3$) | 0.85 (3H, t, 6.9) |
| $N^\delta$-3-OH-Butyryl-$N^\delta$-OH-D-ornithine | NH | - | 8.19 (1H, m) |
| | CO | 172.2 (C) | - |
| | $C_\alpha$ | 50.6 (CH) | 4.21 (1H, m) |
| | $C_\beta$ | 28.0 ($CH_2$) | 1.84 (1H, m), 1.62 (1H, m) |
| | $C_\gamma$ | 21.4 ($CH_2$) | 1.77 (2H, m) |
| | $C_\delta$ | 49.4 ($CH_2$) | 4.00 (1H, m), 3.47 (1H, m) |
| | CO | 161.4 (C) | - |
| | $C'_\alpha$ | 39.4 ($CH_2$) | 2.58 (1H, dd 14.1, 6.7) 2.41 (1H, dd 14.1, 5.7) |
| | $C'_\beta$ | 64.1 (CH) | 3.95 (1H, m) |
| | $C'_\gamma$ | 23.3 ($CH_3$) | 1.11 (3H, d, 6.0) |
| L-*erythro*-3-OH-Aspartate 1 | NH | - | 9.03 (1H, d, 6.3) |
| | CO | 167.4 (C) | - |
| | $C_\alpha$ | 55.1 (CH) | 5.31 (1H, m) |
| | $C_\beta$ | 74.6 (CH) | 4.37 (1H, d, 3.5) |
| | COOH | 176.5 (C) | - |
| L-Threonine | NH | - | 7.33 (1H, d, 8.4) |
| | CO | 170.3 (C) | - |
| | $C_\alpha$ | 58.2 (CH) | 4.15 (1H, d, 8.7) |
| | $C_\beta$ | 65.1 (CH) | 4.43 (1H, m) |
| | $C_\gamma$ | 20.4 ($CH_3$) | 1.06 (3H, d, 6.4) |
| $N^\delta$-OH-D-Ornithine | NH | - | 8.31 (1H, br. s) |
| | CO | 171.5 (C) | - |
| | $C_\alpha$ | 53.3 (CH) | 4.41 (1H, m) |
| | $C_\beta$ | 28.9 ($CH_2$) | 1.94 (1H, m), 1.69 (1H, m) |
| | $C_\gamma$ | 22.5 ($CH_2$) | 1.85 (1H, m), 1.53 (1H, m) |
| | $C_\delta$ | 49.9 ($CH_2$) | 4.00 (1H, m), 3.26 (1H, m) |
| L-Alanine | NH | - | 7.33 (1H, d, 8.4) |
| | CO | 171.0 (C) | - |
| | $C_\alpha$ | 50.6 (CH) | 4.28 (1H, m) |
| | $C_\beta$ | 16.9 ($CH_3$) | 1.30 (3H, d, 7.3) |
| L-*erythro*-3-OH-Aspartate 2 | NH | - | 6.95 (1H, d, 7.9) |
| | CO | 167.5 (C) | - |
| | $C_\alpha$ | 55.8 (CH) | 4.32 (1H, m) |
| | $C_\beta$ | 73.5 (CH) | 3.78 (1H, m) |
| | COOH | 167.9 (C) | - |

**Agar diffusion assay**

The assay was performed according to DIN 58940 guidelines.[21] The initial antimicrobial activity of pandorabactin A (**1**) and B (**2**) was tested against *Bacillus subtilis* subsp. *spizizenii* ATCC 6633 (DSM 347, JMRC: STI10880), *Staphylococcus aureus* SG 511 (DSM 6247, JMRC: STI10760), *Escherichia coli* SG 458 (DSM 30083, JMRC: ST033699), *Pseudomonas aeruginosa* SG 137 (DSM 50071, JMRC: ST033772) and K799/61 (antibiotic-susceptible penetration mutant of DSM 50071, JMRC: ST033771), *Staphylococcus aureus* 134/93 (MRSA) (JMRC: STH00435), *Enterococcus faecalis* 1528 (VRE) (JMRC: ST033700), *Mycobacterium vaccae* (DSM 43514, JMRC: STI10670), *Sporobolomyces salmonicolor* SBUG 0549 (JMRC: ST035974), *Candida albicans* BMSY 212 (JMRC: STI50163), *Penicillium notatum* JP 36 (JMRC: STI50164), *Stenotrophomonas maltophilia* (DSM 50170, JMRC: STI10402) and *Streptococcus mitis* (DSM 12643 JMRC: ST036313). The test strains are stored and maintained at the Jena Microbial Resource Collection (JMRC), a collective strain collection of the Leibniz Institute for Natural Product Research and Infection Biology, HKI and Friedrich Schiller university Jena localized at the Leibniz-HKI Jena, Germany. In a first step, agar plates were inoculated with 100 µL of the test organisms (except 250 µL for *Stenotrophomonas maltophilia* and 300 µL for *Streptococcus mitis* were used) and holes with a diameter of 9 mm were punched into the agar medium. Pandorabactin A (**1**) and B (**2**) (1 mg each) were dissolved in 1 mL MeOH and 50 µL of this solution was added to a hole. Ciprofloxacin (5 µg · $mL^{-1}$ in $H_2O$) and amphotericin B (10 µg · $mL^{-1}$ in methanol with 1% DMSO) were used as positive controls. MeOH was used as a negative control. After the solvent evaporated, the agar plates were incubated according to the growth conditions of the test organisms.

Additionally, the $Fe^{3+}$ complexes of pandorabactin A (**1**) and B (**2**) were tested against *Pseudomonas aeruginosa* SG 137 (DSM 50071, JMRC: ST033772) and K799/61 (antibiotic-susceptible penetration mutant of DSM 50071, JMRC: ST033771), *Mycobacterium vaccae* (DSM 43514, JMRC: STI10670), *Stenotrophomonas maltophilia* (DSM 50170, JMRC: STI10402) and *Streptococcus mitis* (DSM 12643 JMRC: ST036313) as described above.

**Table S14.** Agar diffusion assays with pandorabactin A (**1**) and pandorabactin B (**2**). Ciprofloxacin and amphotericin B were used as positive controls and MeOH was used as negative control. Inhibition zones are given in mm, with P: many colonies in the inhibition zone and p: some colonies in the inhibition zone.

| | Pandorabactin A in MeOH (1 mg · $mL^{-1}$) | Pandorabactin B in MeOH (1 mg · $mL^{-1}$) | Ciprofloxacin (5 µg · $mL^{-1}$) | Amphotericin B (10 µg · $mL^{-1}$) | MeOH |
|---|---|---|---|---|---|
| *Bacillus subtilis* ATCC 6633 | 23P | 22P | 29 | - | 0 |
| *Staphylococcus aureus* SG 511 | 18P | 18P | 20 | - | 0 |
| *Escherichia coli* SG 458 | 18P | 18P | 25/33p | - | 0 |
| *Pseudomonas aeruginosa* SG 137 | 25P | 25P | 25 | - | 10 |
| *Pseudomonas aeruginosa* K799/61 | 25P | 23P | 28/37p | - | 0 |
| *Staphylococcus aureus* 134/94 | 15P | 15P | 0 | - | 0 |
| *Enterococcus faecalis* 1528 | 19P | 17P | 16F | - | 11P |
| Mycobacterium vaccae 10670 | 21p | 22p | 20p | - | 0 |
| *Sporobolomyces salmonicolor* SBUG 549 | 18P | 17P | - | 18p | 10 |
| *Candida albicans* BMSY 212 | 0 | 0 | - | 20 | 0 |
| *Penicillium notatum* JP 36 | 10/20P | 10/18P | - | 17p | 10 |
| *Stenotrophomonas maltophilia* | 22p | 20p | 20p | - | 0 |
| *Streptococcus mitis* | 0 | 0 | 12P* | - | 0 |

* ciprofloxacin with a concentration of 10 µg · $mL^{-1}$ was used

**Table S15.** Agar diffusion assays with $Fe^{3+}$-pandorabactin A (**3**) and $Fe^{3+}$-pandorabactin B (**4**). Ciprofloxacin was used as positive control and $H_2O$ was used as negative control. Inhibition zones are given in mm, with P: many colonies in the inhibition zone and p: some colonies in the inhibition zone.

| | $Fe^{3+}$-pandorabactin A in $H_2O$ (1 mg · $mL^{-1}$) | $Fe^{3+}$-pandorabactin B in $H_2O$ (1 mg · $mL^{-1}$) | Ciprofloxacin (5 µg · $mL^{-1}$) | $H_2O$ |
|---|---|---|---|---|
| *Pseudomonas aeruginosa* SG 137 | 0 | 0 | 25 | 0 |
| *Pseudomonas aeruginosa* K799/61 | 0 | 0 | 25/33p | 0 |
| Mycobacterium vaccae 10670 | 0 | 0 | 21p | 0 |
| *Stenotrophomonas maltophilia* | 0 | 0 | 20p | 0 |
| *Streptococcus mitis* | 0 | 0 | 12P* | 0 |

* ciprofloxacin with a concentration of 10 µg · $mL^{-1}$ was used

**Minimal inhibitory concentration (MIC)**

The assay was performed according to NCCLS guidelines.[22] The minimal inhibitory concentration of pandorabactin A (**1**) and B (**2**) was tested against *Mycobacterium vaccae* (DSM 43514, JMRC: STI10670), *Pseudomonas aeruginosa* SG 137 (DSM 50071, JMRC: ST033772) and K799/61 (antibiotic-susceptible penetration mutant of DSM 50071, JMRC: ST033771) and *Stenotrophomonas maltophilia* (DSM 50170, JMRC: STI10402). Strains were cultivated in the appropriate medium and cell density was adjusted in Müller-Hinton-Bouillion (except for *Stenotrophomonas maltophilia* DSMZ medium M 92 was also used) to barely visible opacity with the McFarland Standard Nr. 0.5, which indicates a bacterial density of $10^8$ cells $mL^{-1}$. 50 µL of pandorabactin A (**1**) and B (**2**) in methanol were serially diluted by factor two with the culture medium in a 96-well plate starting with a concentration of 100 µg · $mL^{-1}$. Then, the wells were inoculated with the test organisms. The optical density was measured with the microplate reader Infinite$^R$ 200 Pro (TECAN, Männedorf, Switzerland) directly after inoculation and after 24 h of incubation. MIC was read as the lowest concentration that causes inhibition after 24 h of incubation. The test was performed in two technical replicates.

**Table S16.** MIC values in µg ·$mL^{-1}$ of pandorabactin A (**1**) and pandorabactin B (**2**) on different test organisms. Ciprofloxacin was used as positive controls and MeOH was used as negative control.

| | Pandorabactin A in MeOH | Pandorabactin B in MeOH | Ciprofloxacin | MeOH |
|---|---|---|---|---|
| Mycobacterium vaccae IMET 10670 | 12.5 | 12.5 | 0.4 | 100 |
| *Pseudomonas aeruginosa* SG 137 | 12.5 | 12.5 | 0.2 | 100 |
| *Pseudomonas aeruginosa* K 799/61 | 12.5 | 12.5 | 0.4 | 100 |
| *Stenotrophomonas maltophilia*# | 25 | 25 | 0.4 | 50 |
| *Stenotrophomonas maltophilia** | 50 | 50 | 0.2 | >100 |

# adjusted and cultivated in DSMZ medium M 92. * adjusted and cultivated in Müller-Hinton-Bouillion.

**MALDI mass spectrometry imaging**

MALDI-MSI was conducted on a timsTOF fleX MALDI-2 (Bruker, Bremen, Germany) in positive ion mode at a lateral step size of 100 µm and 500 laser pulses at 10 kHz repetition rate, per pixel. Mass spectra were acquired in the mass range from mass range from 215 Da to 1520 Da, while optimizing the ion optical parameters for the transmission of ions with *m/z* ≥ 800 Da. The laser energy was optimized on an experiment-to-experiment basis, to yield acceptable signal-to-noise for *m/z* 1129.42 Da, while simultaneously minimize the amount of matrix cluster ions.

The bacterial co-cultures were prepared for MALDI-MSI by submerging indium ton oxide coated (ITO) glass slides (Bruker) in the iron-deficient MM9 agar.[23-24] The co-cultured bacteria (3 µL) were inoculated in a distance of 1.5 cm on the agar above the glass slides. The ITO glass slides were cut out of the agar after 6 days of incubation at 30 °C and subsequently dried for sixteen hours at 40 °C. The dried samples were spray coated with α-Cyano-4-hydroxycinnamic acid (CHCA) matrix (Bruker) in a M3$^+$ Sprayer (HTX Technologies LLC, Chapel Hill NC, USA) by applying 5 layers of 10mg · $mL^{-1}$ CHCA in 3:1 acetonitrile:water, at 120µl · $min^{-1}$ flow rate,

a line spacing of 2 mm, 75 °C nozzle temperature, and a nozzle speed of 1200mm · min. The drying time between each layer was thirty seconds.

The resulting datasets were analyzed in the Lipostar MSI2 software[25] (v2.0.0, Mass Analytica, Sant Cugat del Vallés, Spain) by first converting to the .imzML data format and importing with the following parameters: a resolving power of 50000 at *m/z* 200, Savitzky-Golay smoothing of 2nd degree, with a window size of 7 and one iteration. The baseline was corrected with a segment size of 0.1 amu. Peaks were picked with a minimum SNR of 1.0, a noise window size of 0.1 amu, 0.00 minimum absolute intensity. Features with an intensity of less than 1.0% of the base peak were discarded. The minimum peak frequency was set to 1.0% with a minimal spatial chaos of 0.9. The spatial distributions of the picked peaks were calculated with a *m/z* tolerance of 0.017 amu, a mass error of 5 ppm, and applying hotspot removal by quantile thresholding (99.75 high quantile) as well as root mean square normalization.

**Cultivation of monocultures of *P. aeruginosa*, *P. sputorum* and *P. oxalativorans* ∆*panI* and co-cultures**

The bacteria were precultured in 2.5 mL iron-deficient MM9 medium with orbital shaking at 100 × *g* and 30 °C overnight. The obtained cultures were added to 20 mL iron-deficient MM9 medium with a starting $OD_{600}$ of 0.05 and 0.07 for *P. oxalativorans* ∆*panI* with orbital shaking at 100 × *g* and 30 °C until the cultures reached an $OD_{600}$ of 0.25. For co-cultivation (three biological replicates) 3 µL of each strain were placed on an iron-deficient MM9 medium agar plate in a distance of 1.5 cm between the strains. For monocultures (three biological replicates) 3 µL were placed in the middle of an iron deficient MM9 medium agar plate. The cultures were grown for 6 days at 30 °C. The assay was performed in triplicates.

**Extraction of monocultures of *P. aeruginosa*, *P. sputorum* and *P. oxalativorans* ∆*panI* and co-cultures**

Interaction area of the co-cultures and the area next to the monocultures was cut out and suspended in 3 mL of MeOH. After 3 h the supernatant was dried under a nitrogen stream at 40 °C. The extracts were redissolved in MeOH for HPLC-HRMS/MS analysis. For determination of pyoverdines in the extracts the peak area was measured and normalized with the weight of the used agar section.

**Table S17.** Determination of pyoverdine (**10**) in mono- and co-cultures.

| Culture | Pyoverdine average |
|---|---|
| *P. aeruginosa* | 3384.75 |
| *P. sputorum* with *P. aeruginosa* | 30727.67 |
| *P. oxalativorans* ∆*panI* with *P. aeruginosa* | 9362.32 |

**Metagenomic analysis**

The taxonomic profiling method of shotgun metagenomic sequencing data was described in Mirhakkak *et al.*[7] To determine the presence of the *pan* BGC in each lung sample, sequencing reads were aligned to the reference *pan* BGC sequence of *P. sputorum* by Diamond (v2.1.9.163).[26] A sample was considered to possess the *pan* BGC if it contained *Pandoraea* species and exhibited mapped reads corresponding to at least six core biosynthetic genes. Statistical different abundant species between samples with/without *pan* BGC were obtained by DESeq2.[27] The raw p values were corrected by applying Benjamini-Hochberg procedure. The R package genomes (v1.0.0) was used to draw read coverage of *pan* BGC.

**Table S18.** Fold-change values of the species detected in sputum samples from cystic fibrosis patients that differ significantly in abundancy when *pan* genes are present or absent.

| Bacterial organism | Mean abundance (%) | $log_2FC$ | p | adjusted p |
|---|---|---|---|---|
| *Prevotella jejuni* | 2.39 | –4.14 | 0.003 | 0.022 |
| *Schaalia odontolytica* | 1.82 | –2.98 | 0.040 | 0.098 |
| *Veillonella parvula* | 1.29 | –2.74 | 0.043 | 0.098 |
| *Veillonella atypica* | 2.04 | –2.80 | 0.027 | 0.078 |
| *Streptococcus pneumoniae* | 1.67 | –3.40 | 0.007 | 0.033 |
| *Streptococcus oralis* | 1.50 | –2.93 | 0.007 | 0.033 |
| *Streptococcus mitis* | 5.36 | –3.62 | 0.001 | 0.013 |
| *Staphylococcus aureus* | 12.2 | –5.56 | 0.0001 | 0.002 |
| *Haemophilus influenzae* | 8.06 | –3.84 | 0.023 | 0.077 |
| *Stenotrophomonas maltophilia* | 14.2 | –8.46 | 0.013 | 0.050 |

**NMR spectra of pandorabactin A (1)**

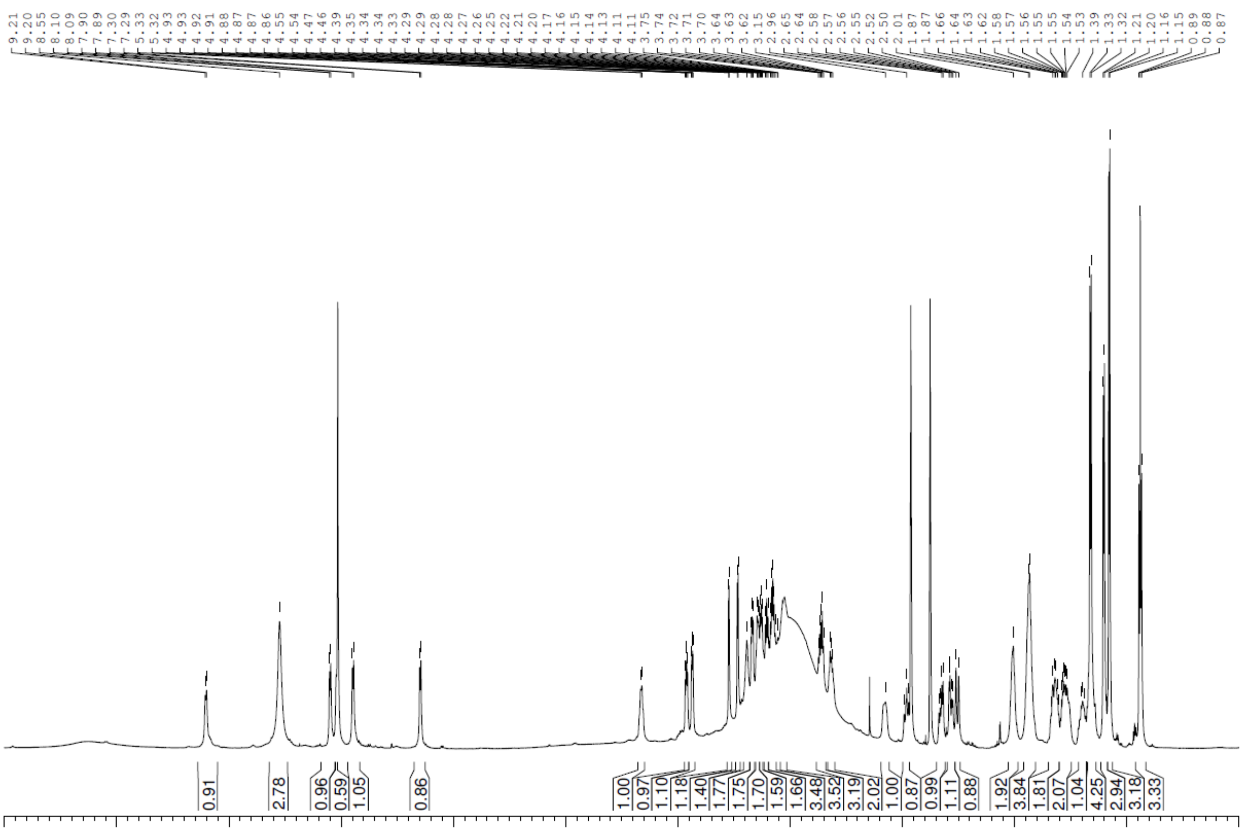


**Figure S21.** $^{1}$H-NMR spectrum of pandorabactin A (**1**) in DMF-$d_7$ (600 MHz).

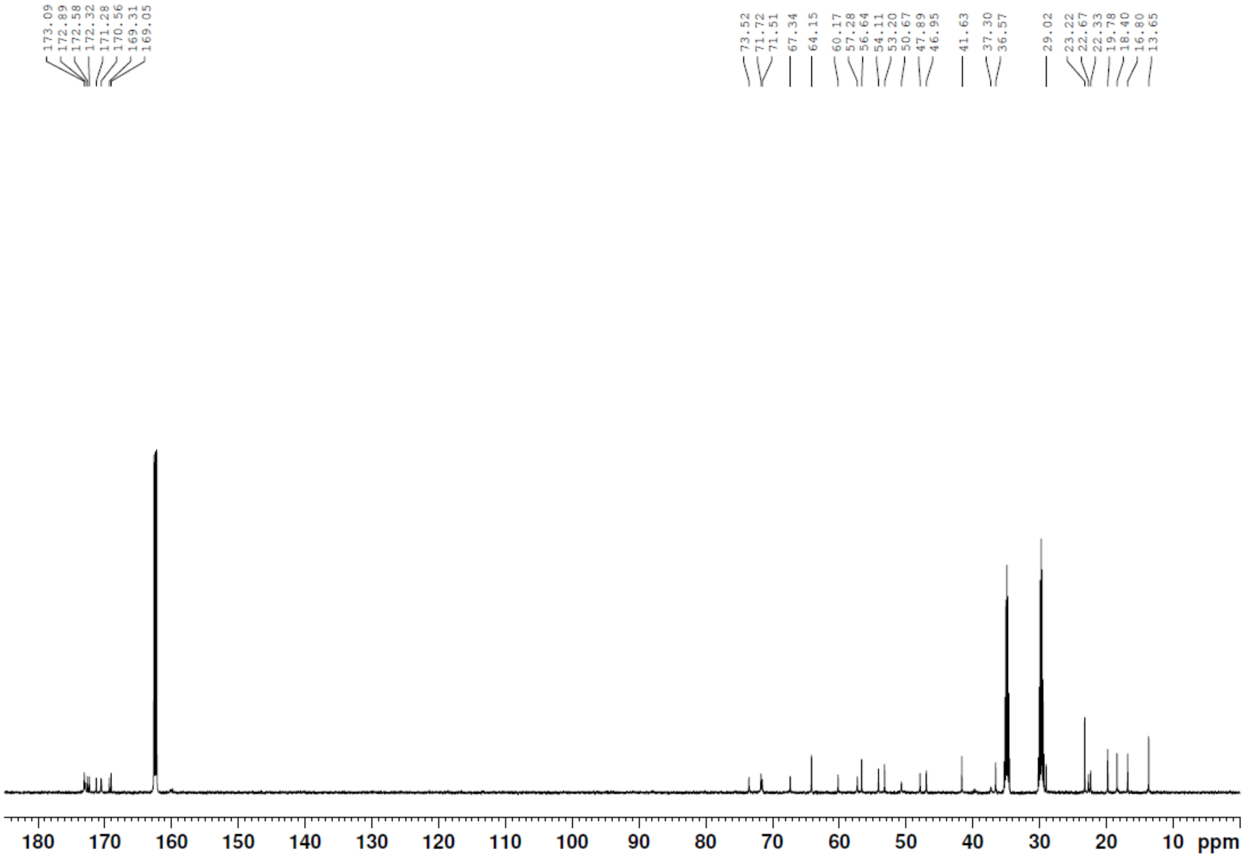


**Figure S22.** $^{13}$C-NMR spectrum of pandorabactin A (**1**) in DMF-$d_7$ (150 MHz).

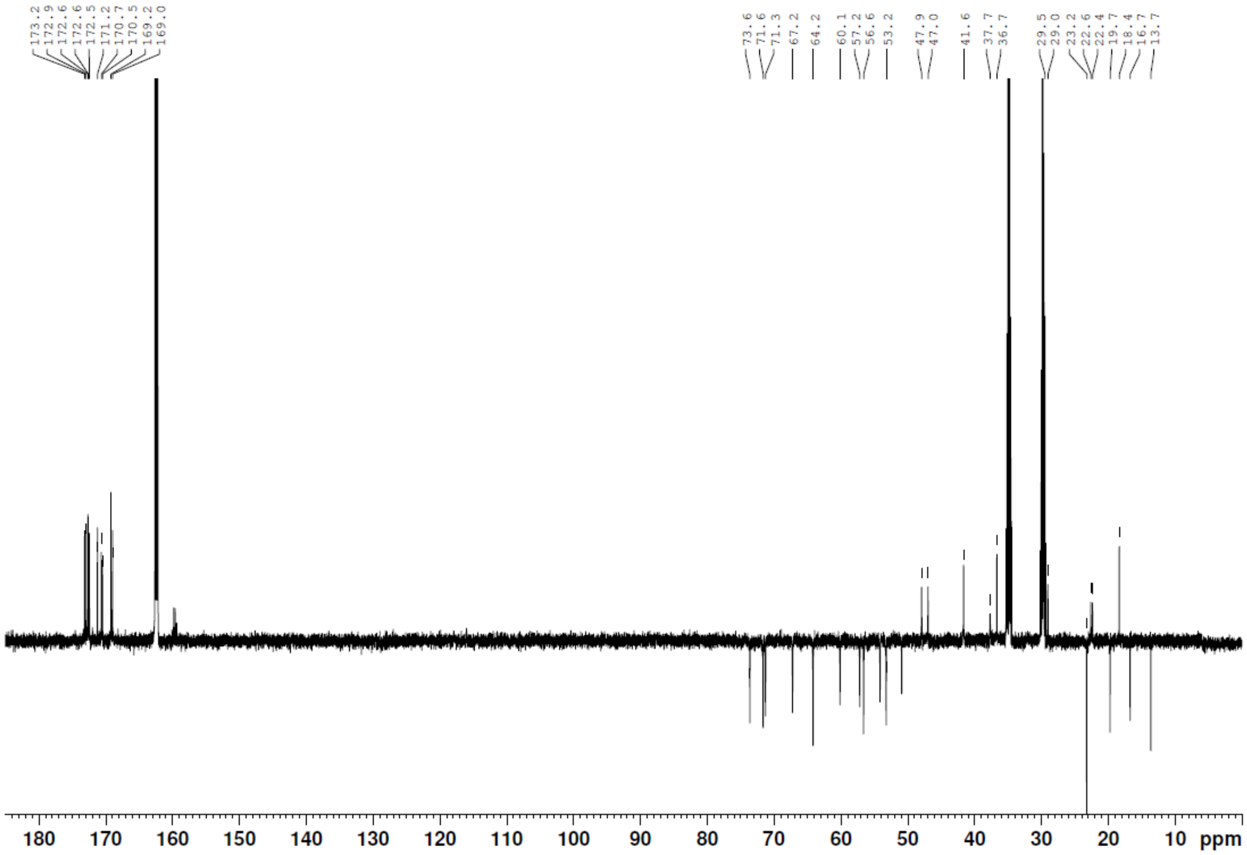


**Figure S23.** $^{13}C$-Jmod-NMR spectrum of pandorabactin A (**1**) in DMF-$d_7$ (150 MHz, C and $CH_2$ are positive, CH and $CH_3$ are negative).

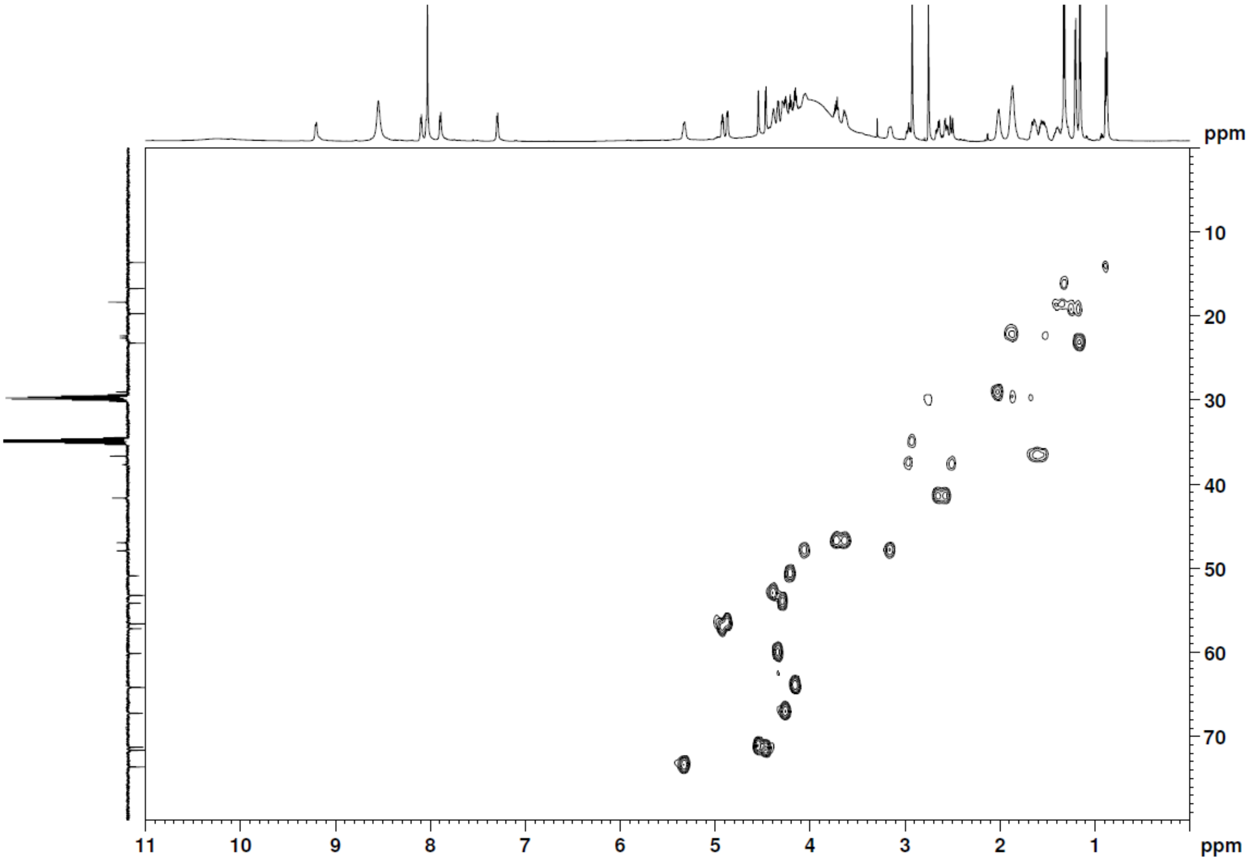


**Figure S24.** $^{1}H$-$^{13}C$-HSQC spectrum of pandorabactin A (**1**) in DMF-$d_7$ (600 MHz).

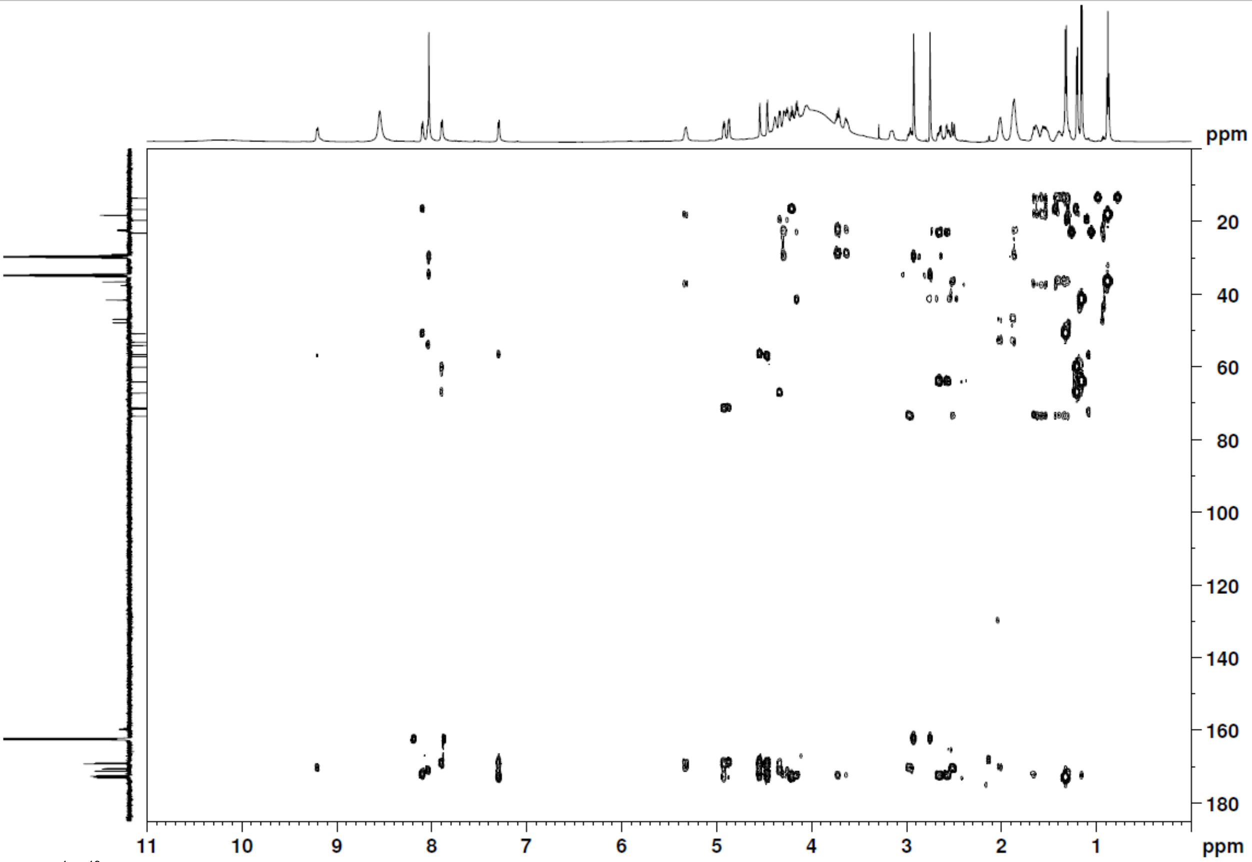


**Figure S25.** $^{1}H$-$^{13}C$-HMBC spectrum of pandorabactin A (**1**) in DMF-$d_7$ (600 MHz).

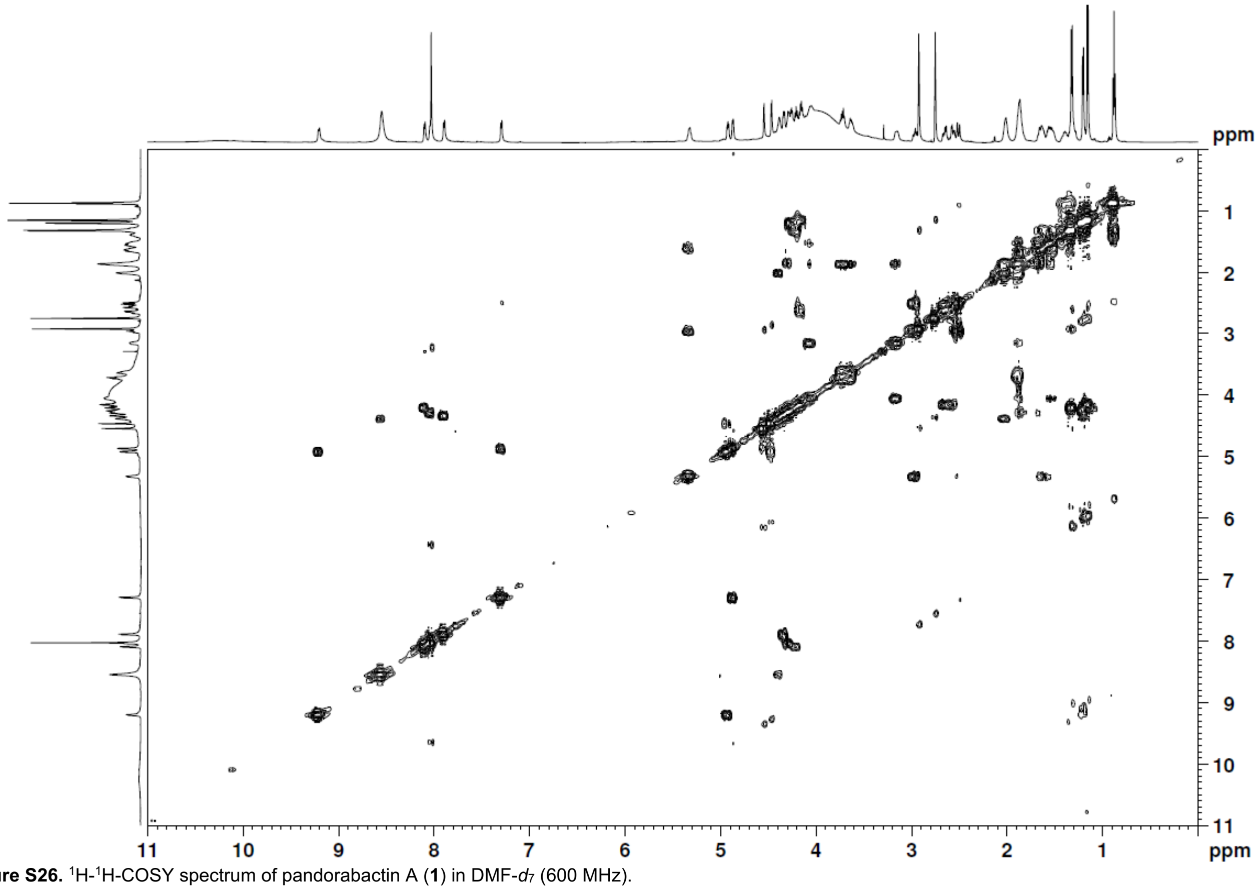


**Figure S26.** $^{1}H$-$^{1}H$-COSY spectrum of pandorabactin A (**1**) in DMF-$d_7$ (600 MHz).

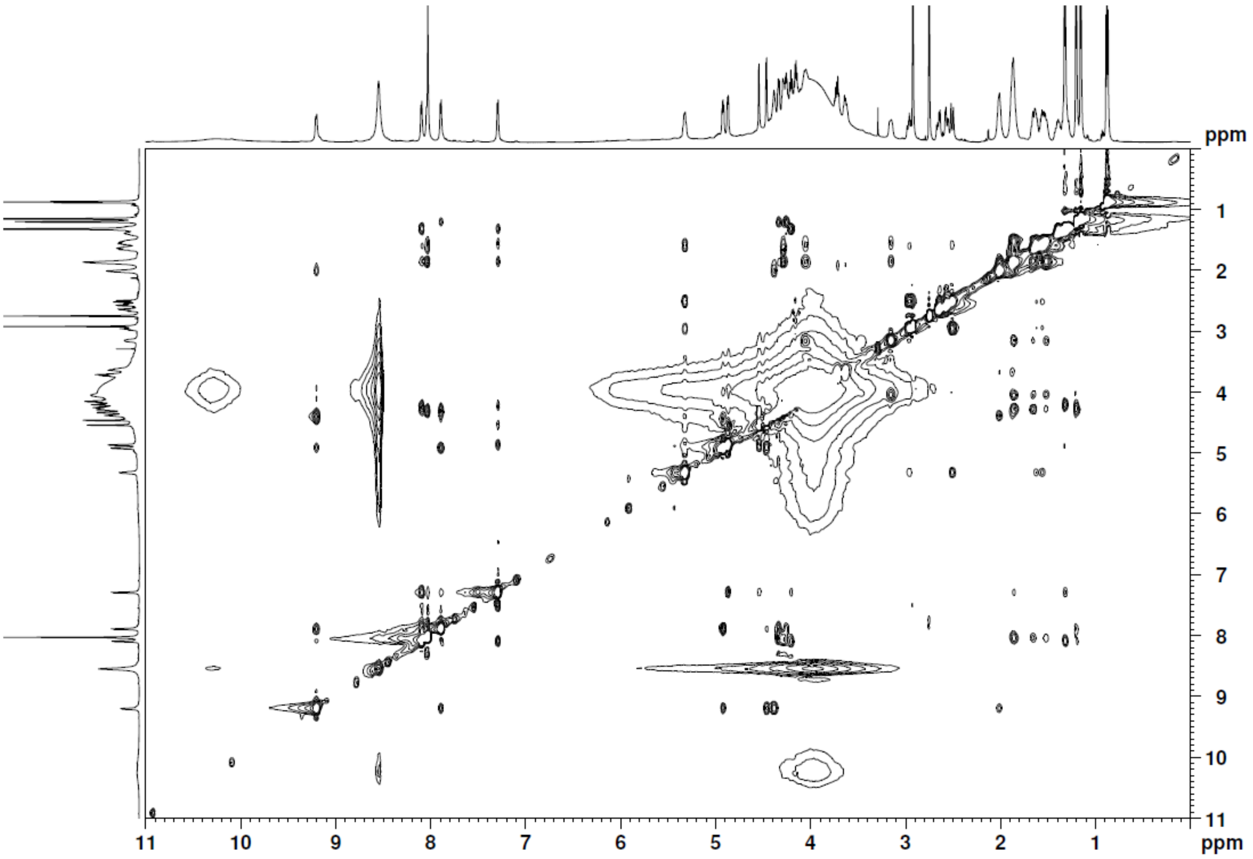


**Figure S27.** $^{1}H$-$^{1}H$-NOESY spectrum of pandorabactin A (**1**) in DMF-$d_7$ (600 MHz).

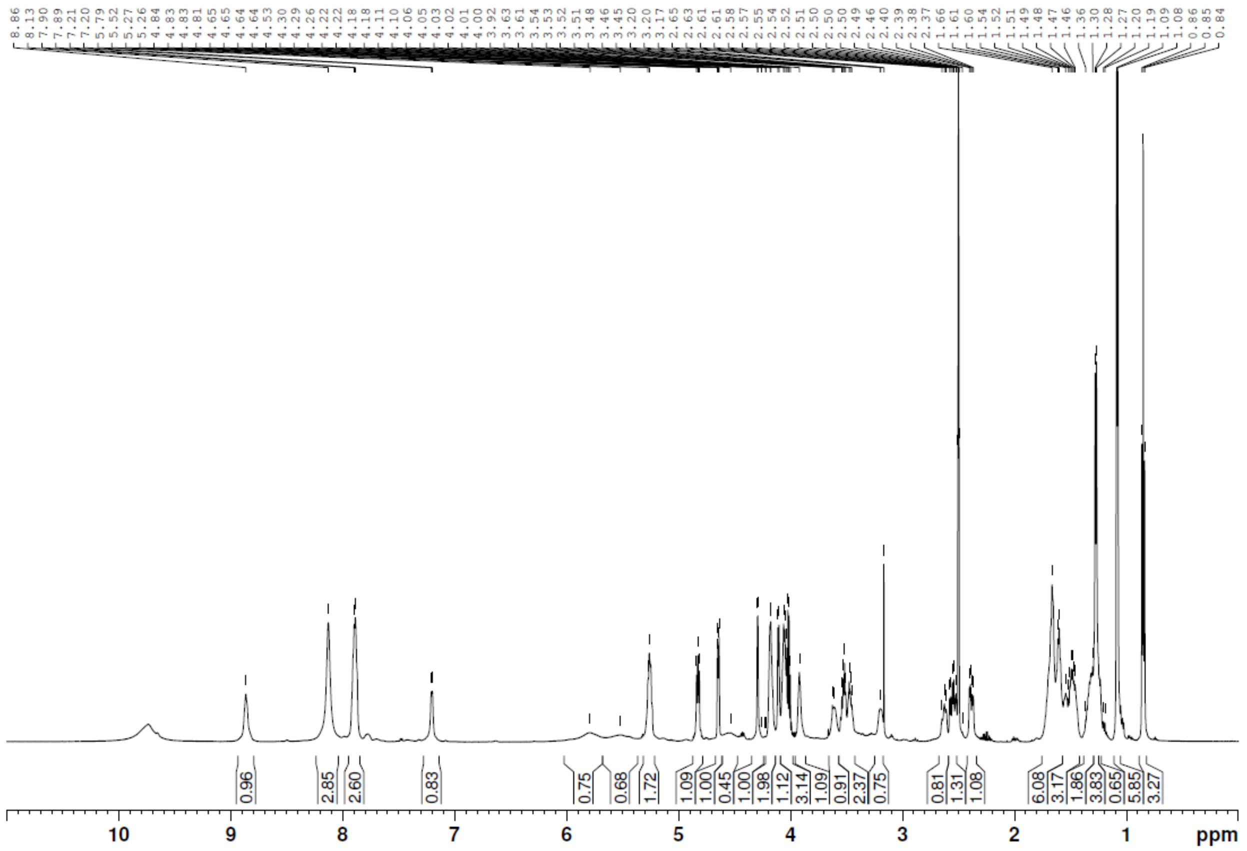


**Figure S28.** $^{1}H$-NMR spectrum of pandorabactin A (**1**) in DMSO-$d_6$ (600 MHz).

**NMR spectra of pandorabactin B (2)**

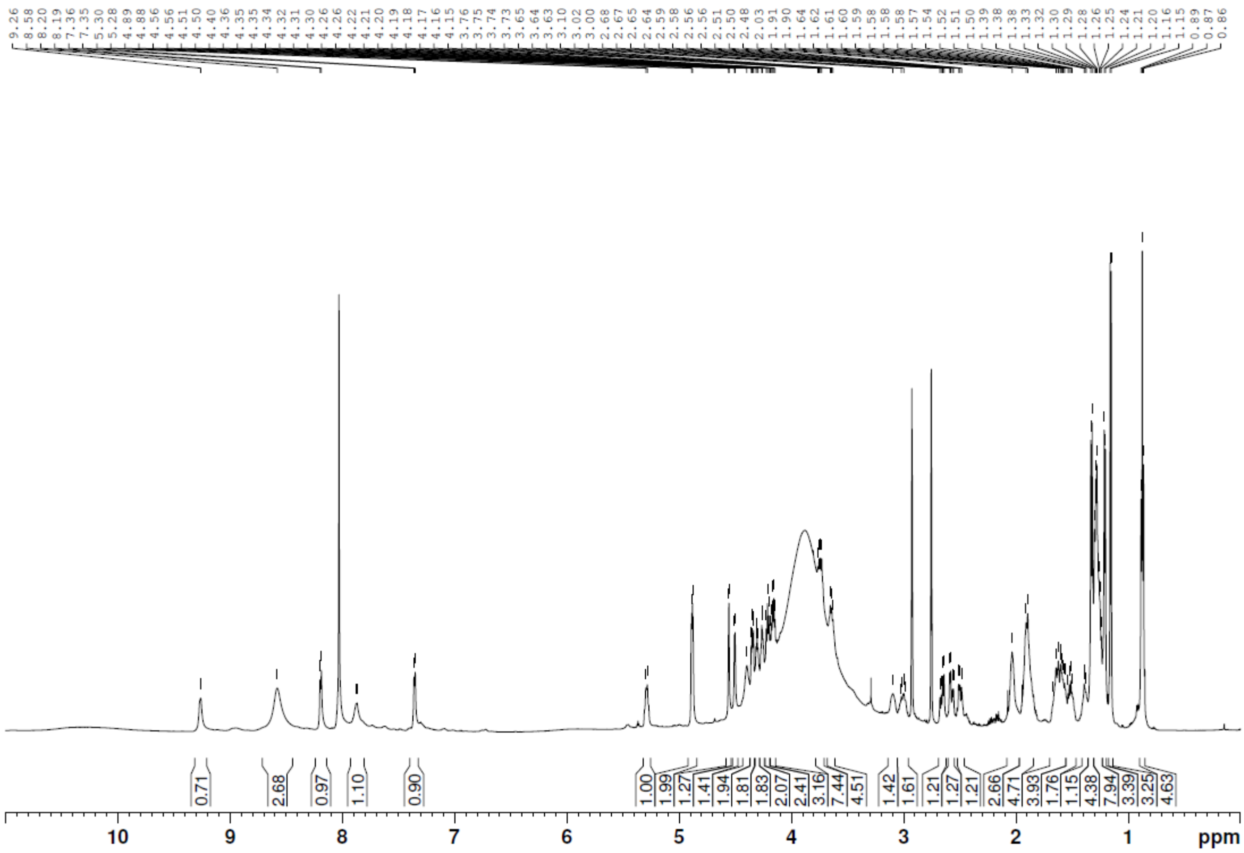


**Figure S29.** $^{1}$H-NMR spectrum of pandorabactin B (**2**) in DMF-$d_7$ (600 MHz).

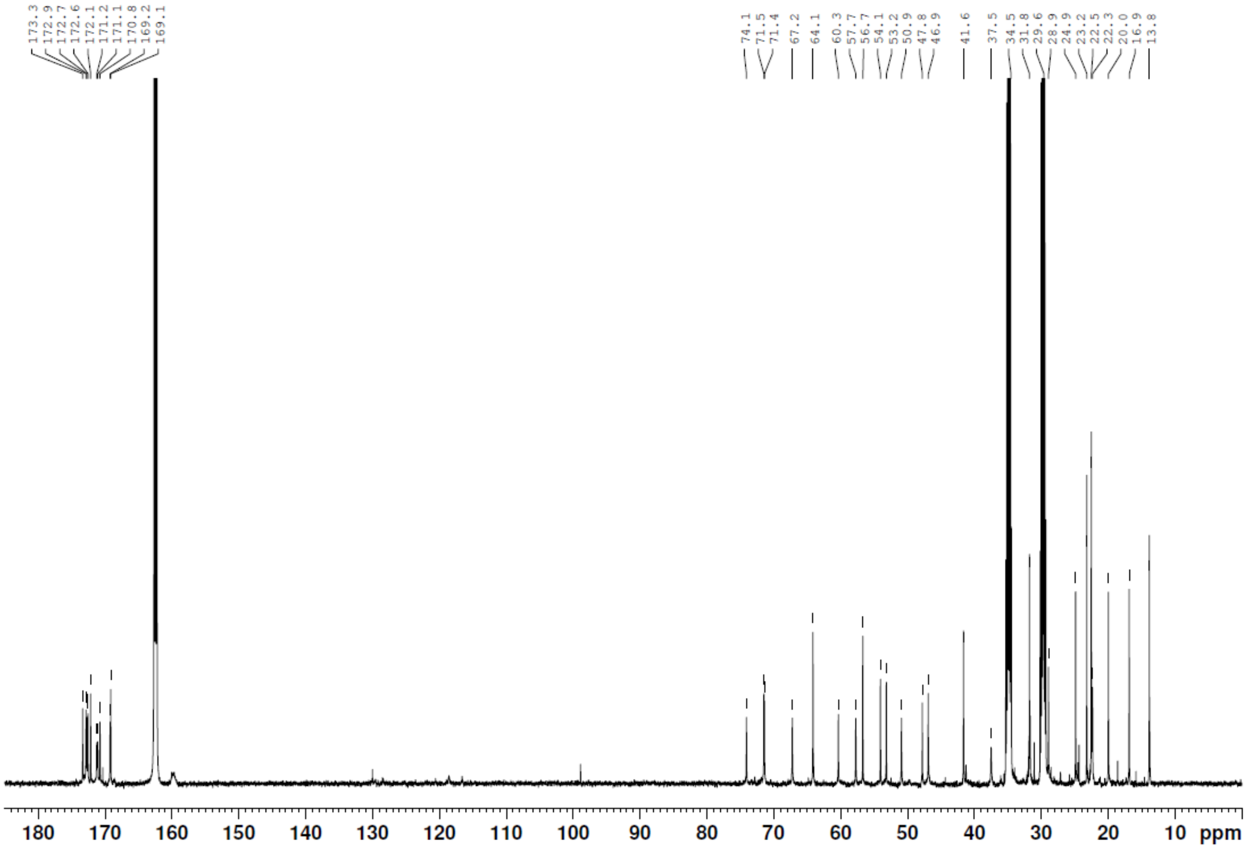


**Figure S30.** $^{13}$C-NMR spectrum of pandorabactin B (**2**) in DMF-$d_7$ (150 MHz).

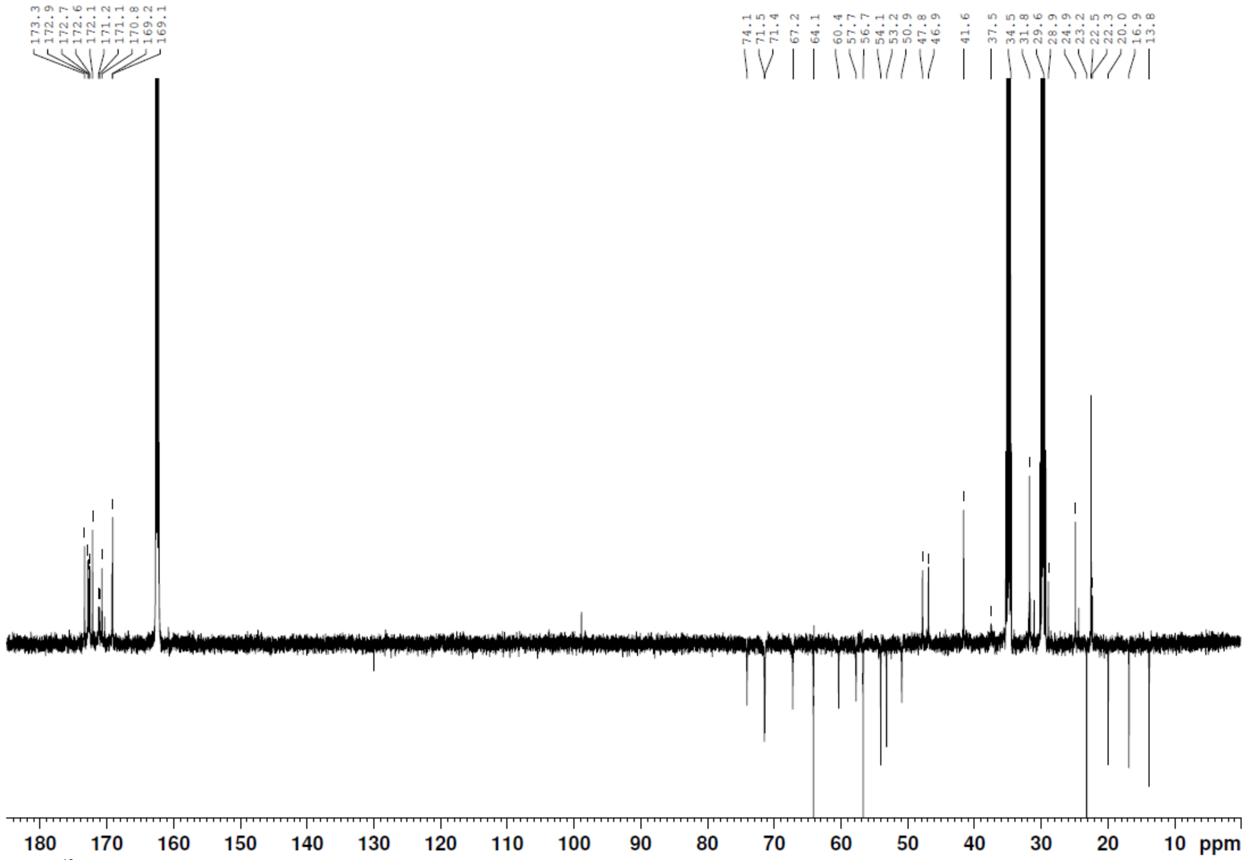


**Figure S31.** $^{13}$C-Jmod-NMR spectrum of pandorabactin B (**2**) in DMF-*d*$_7$ (150 MHz, C and $CH_2$ are positive, CH and $CH_3$ are negative).

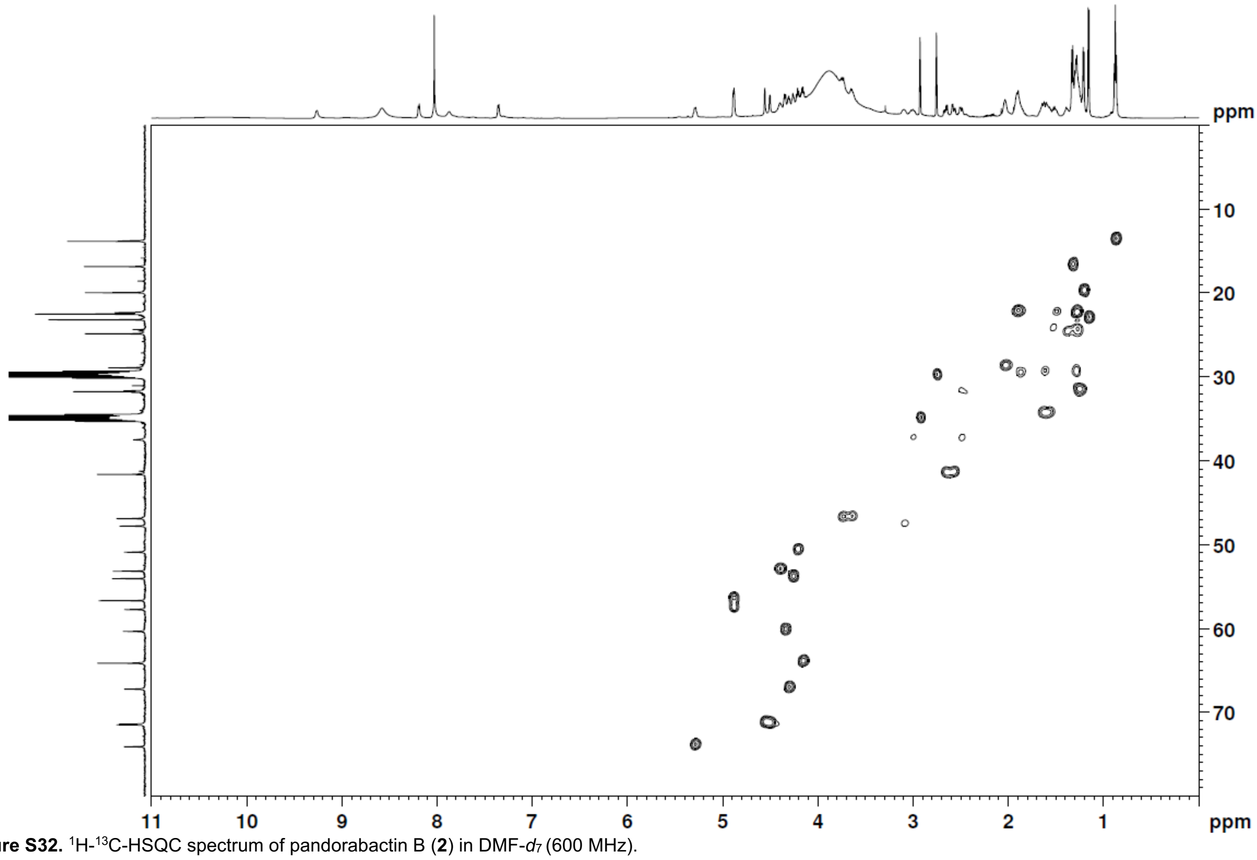


**Figure S32.** $^{1}$H-$^{13}$C-HSQC spectrum of pandorabactin B (**2**) in DMF-*d*$_7$ (600 MHz).

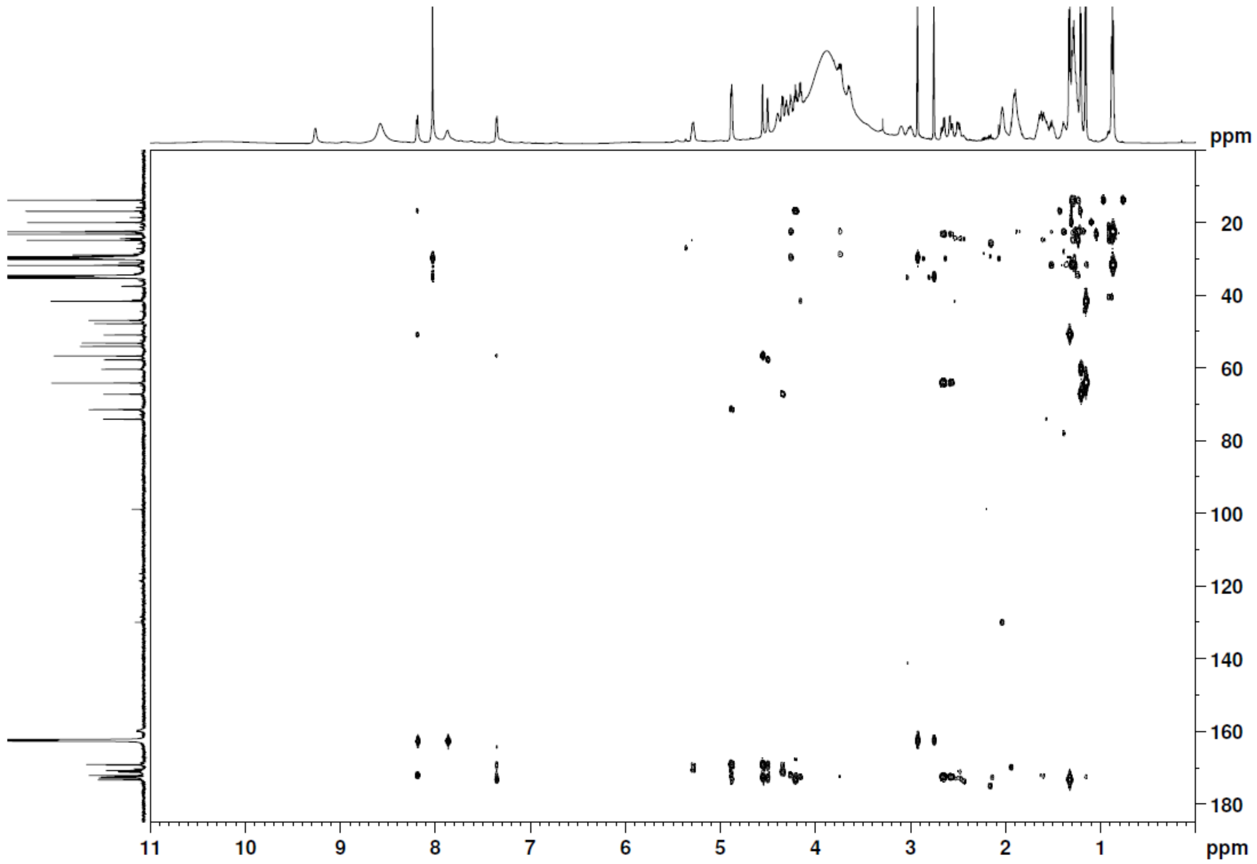


**Figure S33.** $^{1}H$-$^{13}C$-HMBC spectrum of pandorabactin B (**2**) in DMF-*d*₇ (600 MHz).

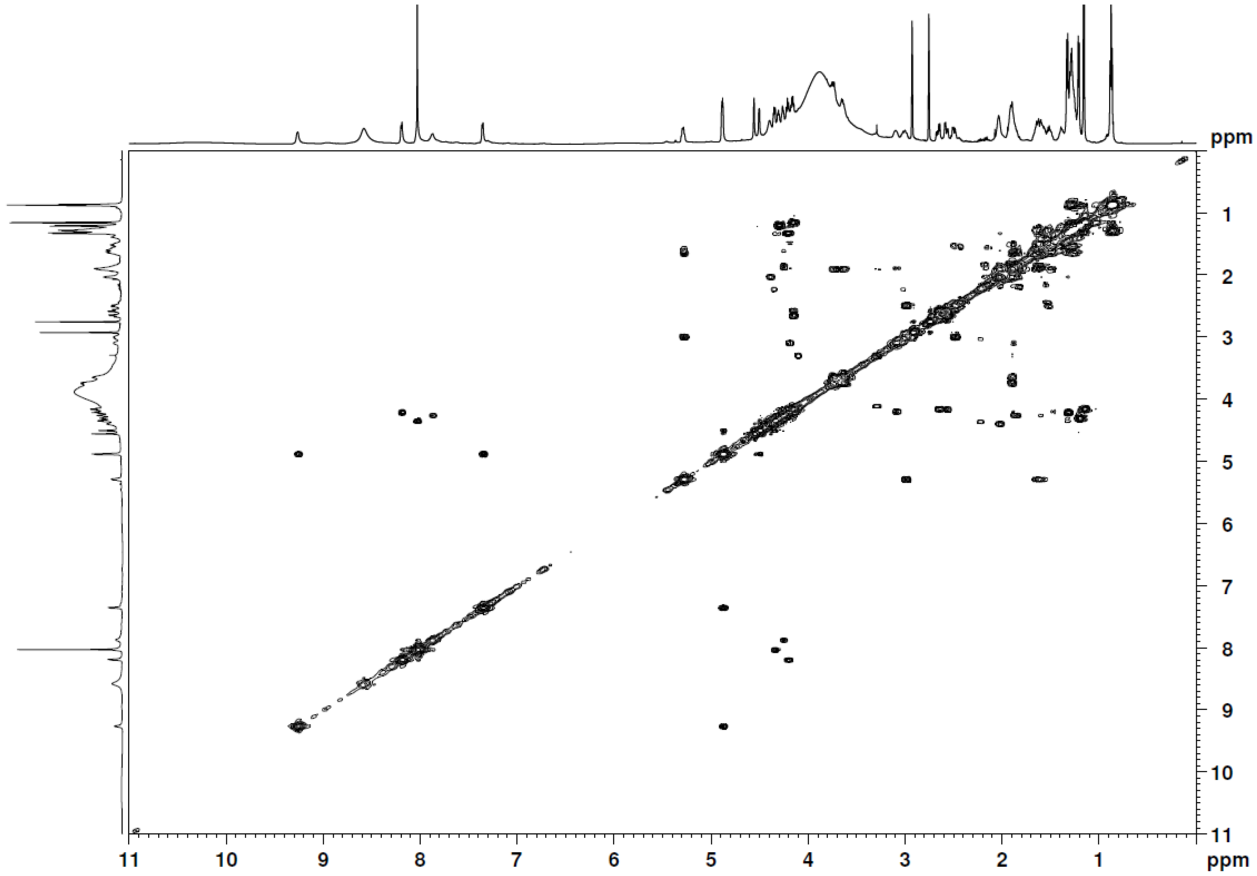


**Figure S34.** $^{1}H$-$^{1}H$-COSY spectrum of pandorabactin B (**2**) in DMF-*d*₇ (600 MHz).

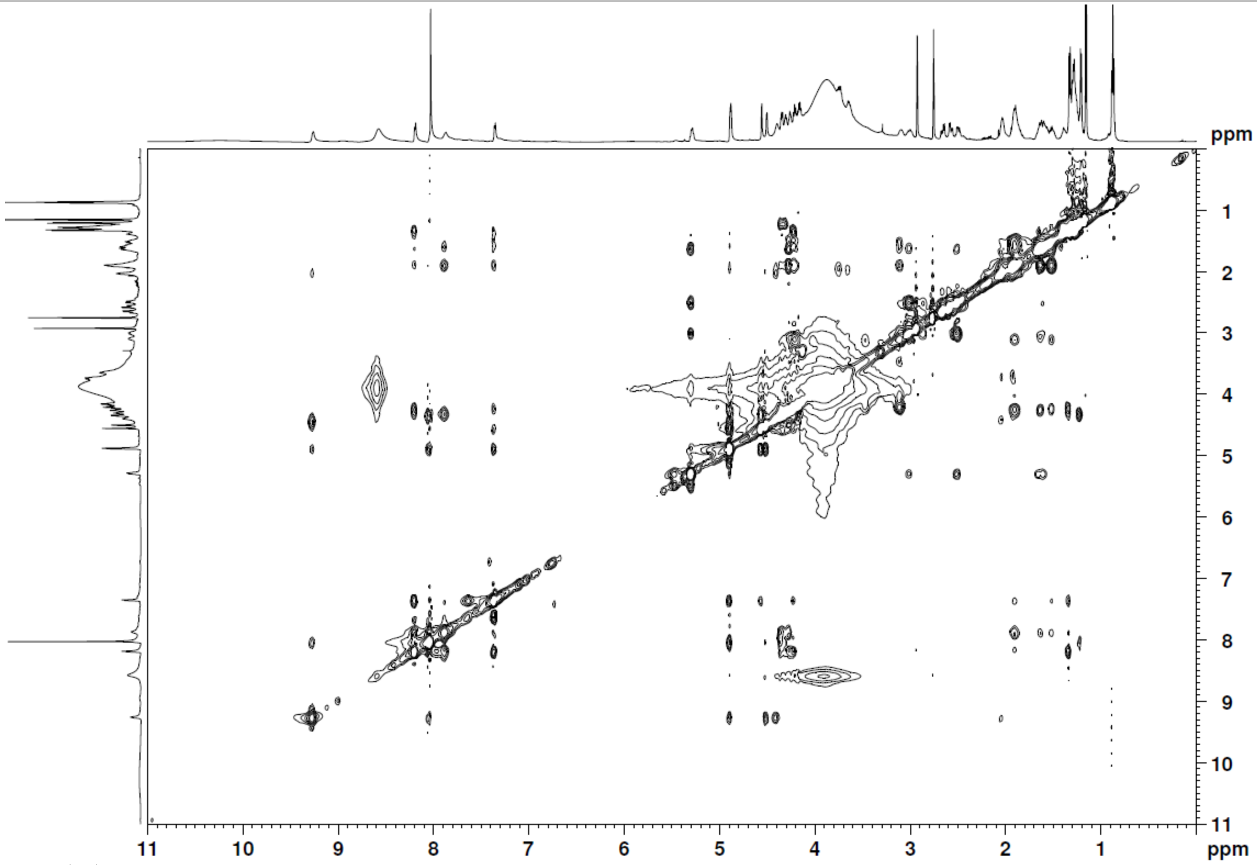


**Figure S35.** $^{1}H$-$^{1}H$-NOESY spectrum of pandorabactin B (**2**) in DMF-$d_7$ (600 MHz).

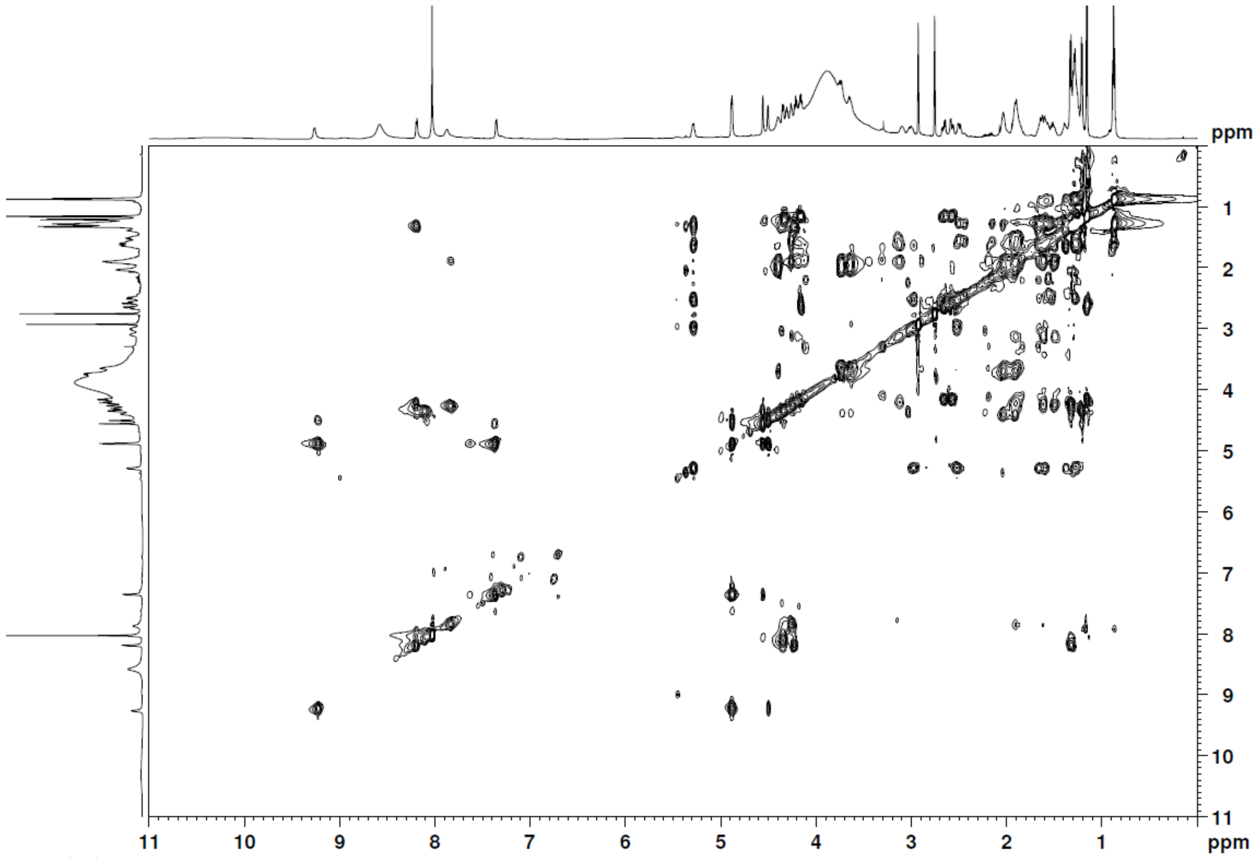


**Figure S36.** $^{1}H$-$^{1}H$-TOCSY spectrum of pandorabactin B (**2**) in DMF-$d_7$ (600 MHz).

**NMR spectra of *N*$^{\delta}$-benzoyloxy-*N*$^{\alpha}$-Boc-L-ornithine *tert*-butyl ester (13)**

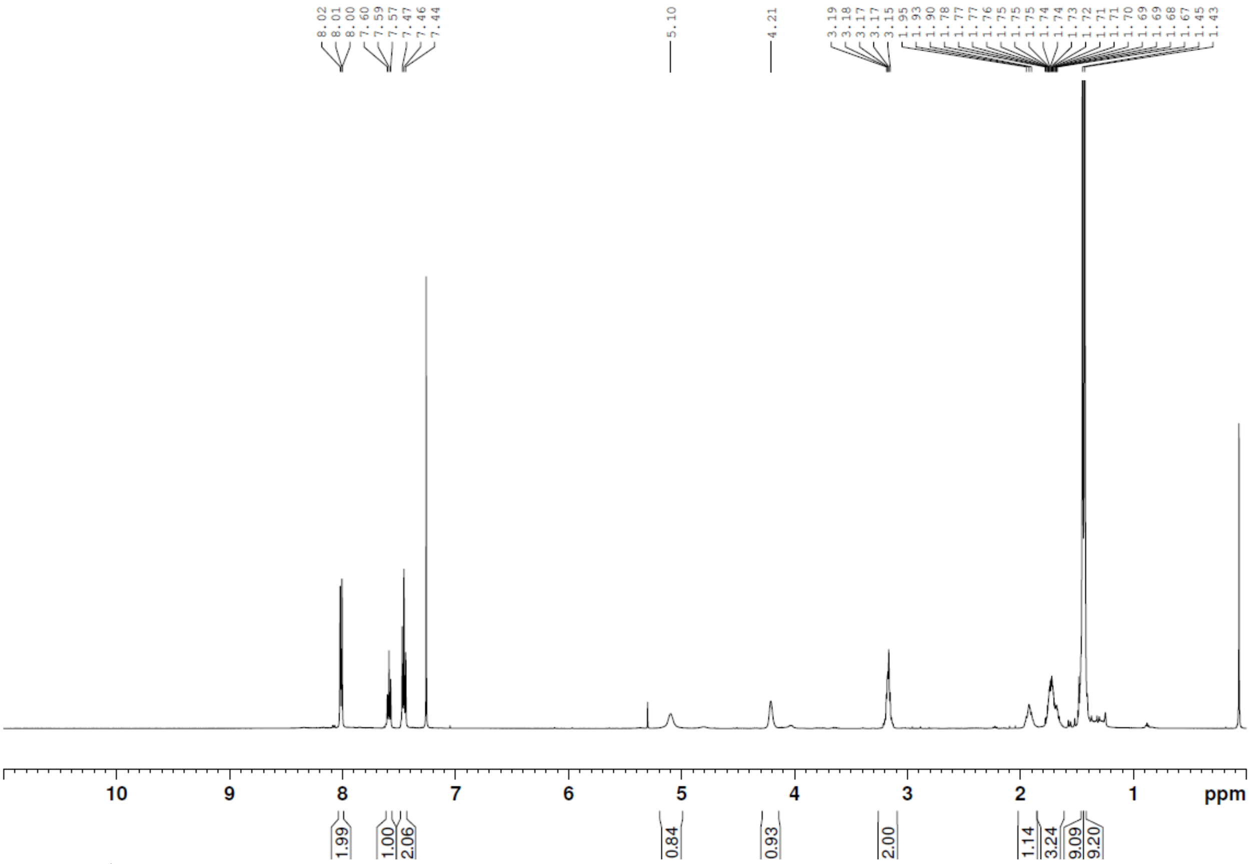

**Figure S37.** $^{1}$H-NMR spectrum of *N*$^{\delta}$-benzoyloxy-*N*$^{\alpha}$-Boc-L-ornithine *tert*-butyl ester (**13**) in $CDCl_3$ (500 MHz).

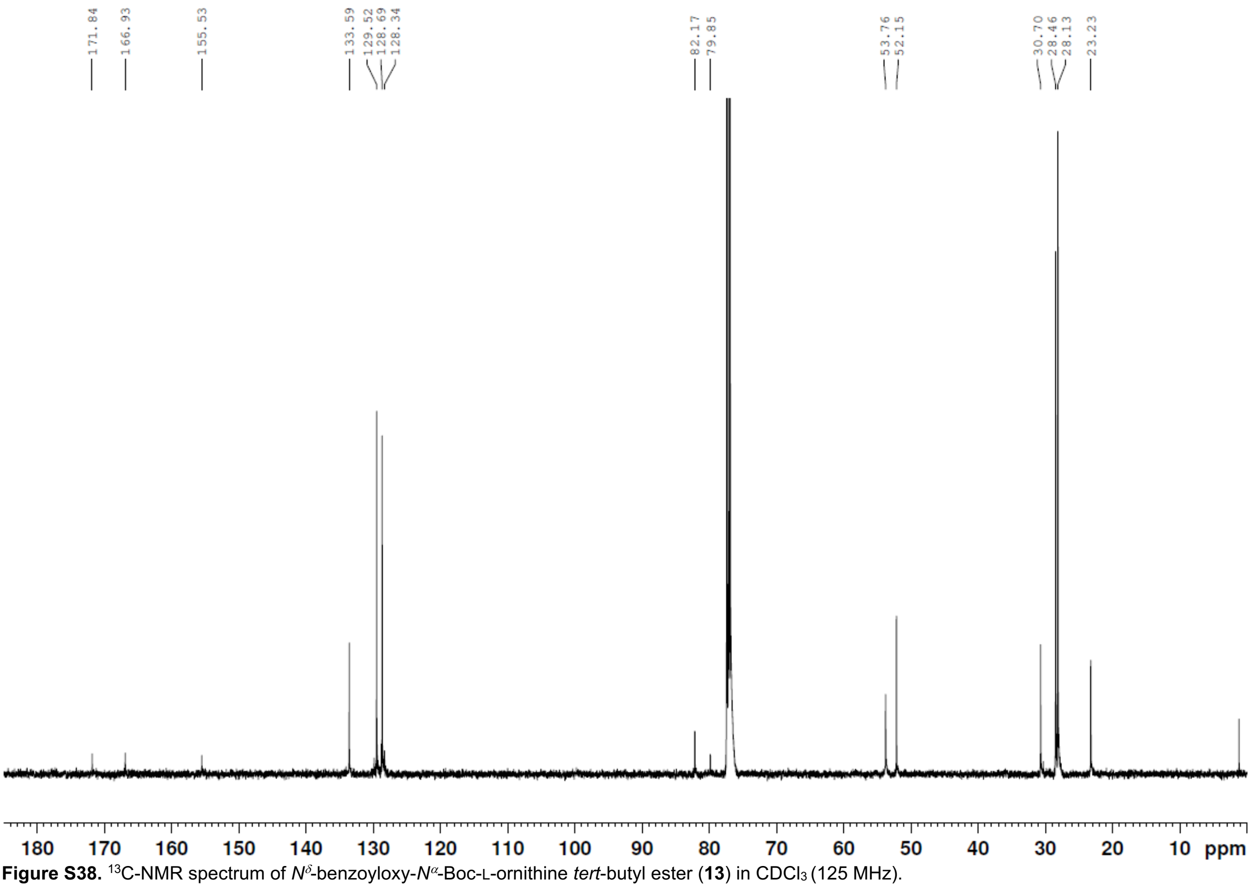

**Figure S38.** $^{13}$C-NMR spectrum of *N*$^{\delta}$-benzoyloxy-*N*$^{\alpha}$-Boc-L-ornithine *tert*-butyl ester (**13**) in $CDCl_3$ (125 MHz).

**NMR spectra of *N*$^{\delta}$-benzoyloxy-*N*$^{\delta}$-(*R*)-3-hydroxy-butyryl-*N*$^{\alpha}$-Boc-L-ornithine *tert*-butyl ester (14)**

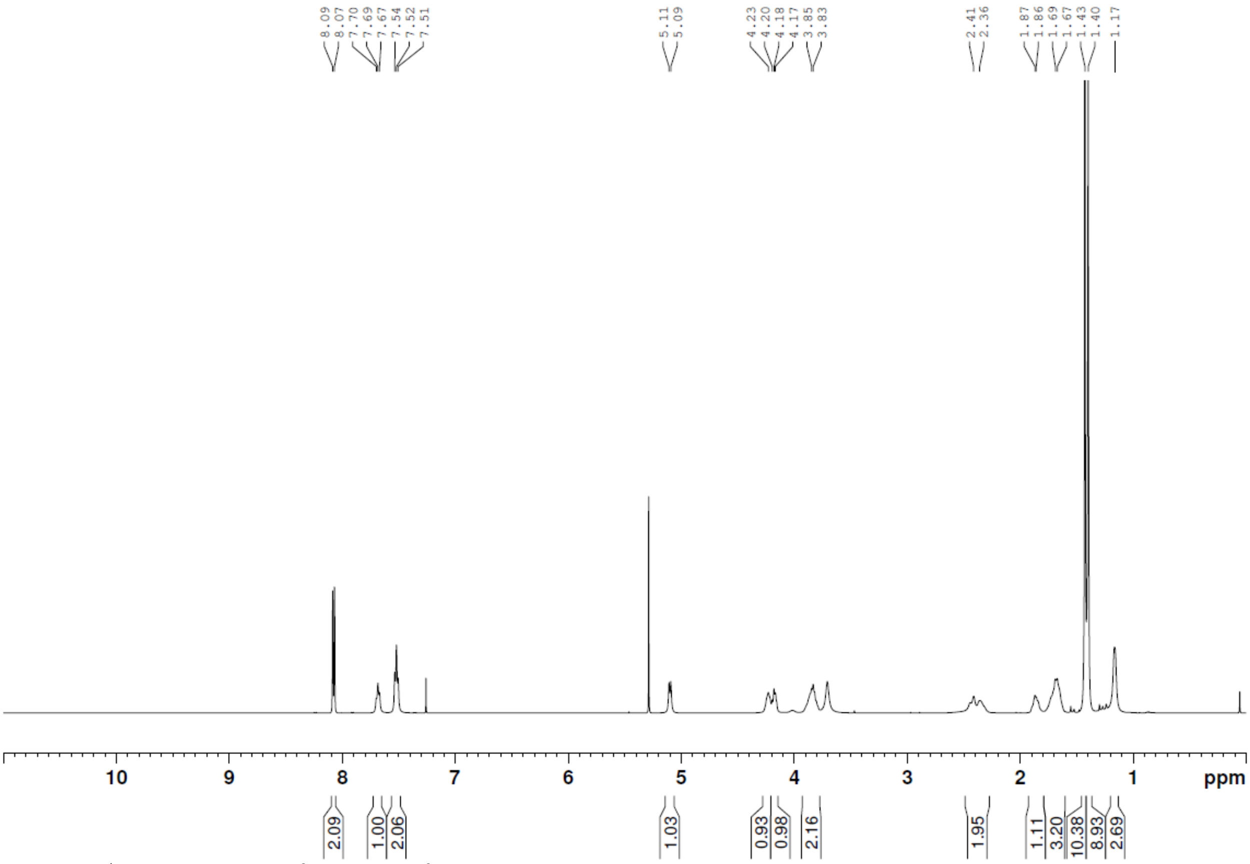


**Figure S39.** $^{1}$H-NMR spectrum of *N*$^{\delta}$-benzoyloxy-*N*$^{\delta}$-(*R*)-3-hydroxy-butyryl-*N*$^{\alpha}$-Boc-L-ornithine *tert*-butyl ester (**14**) in $CDCl_3$ (500 MHz).

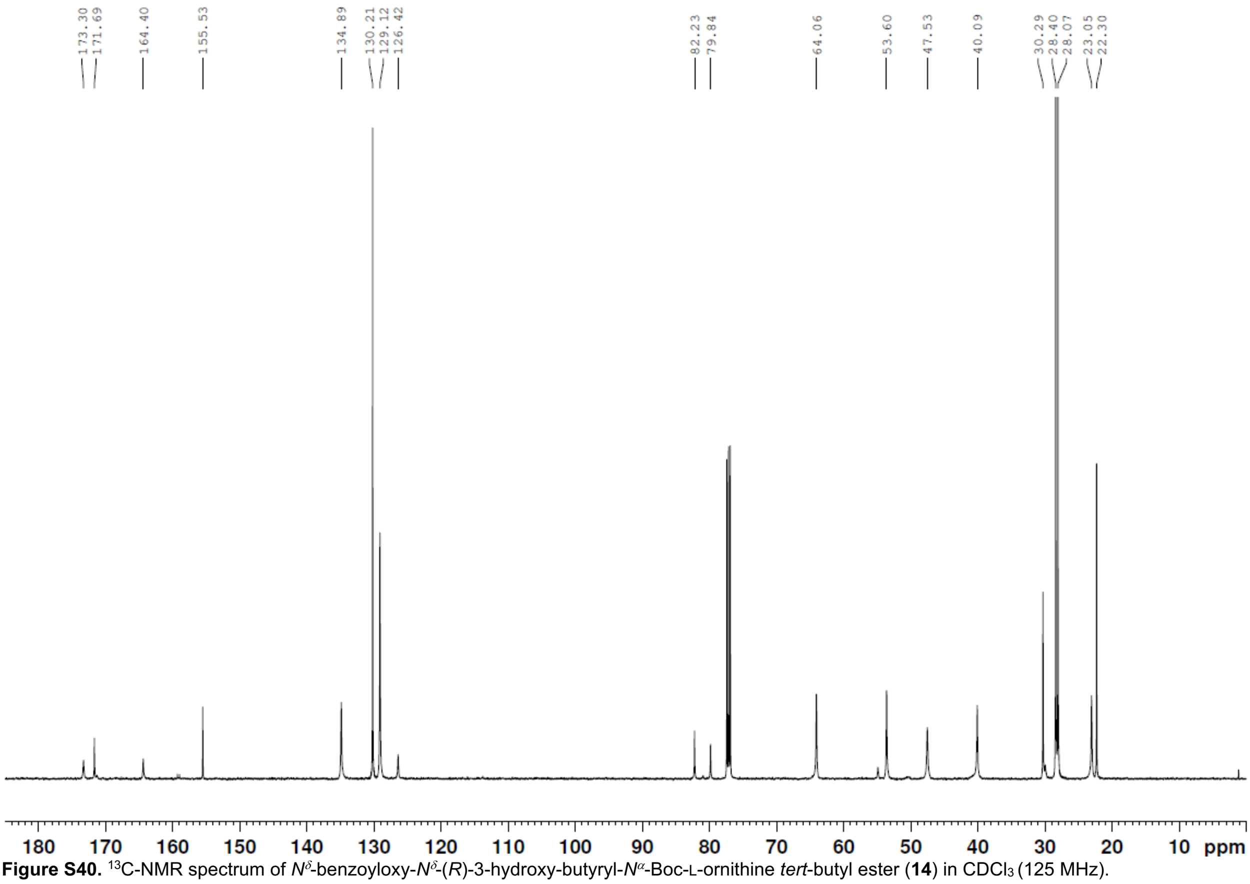


**Figure S40.** $^{13}$C-NMR spectrum of *N*$^{\delta}$-benzoyloxy-*N*$^{\delta}$-(*R*)-3-hydroxy-butyryl-*N*$^{\alpha}$-Boc-L-ornithine *tert*-butyl ester (**14**) in $CDCl_3$ (125 MHz).

**NMR spectrum of *N*$^{\delta}$-hydroxy-*N*$^{\delta}$-(*R*)-3-hydroxy-butyryl-*N*$^{\alpha}$-Boc-L-ornithine *tert*-butyl ester (15)**

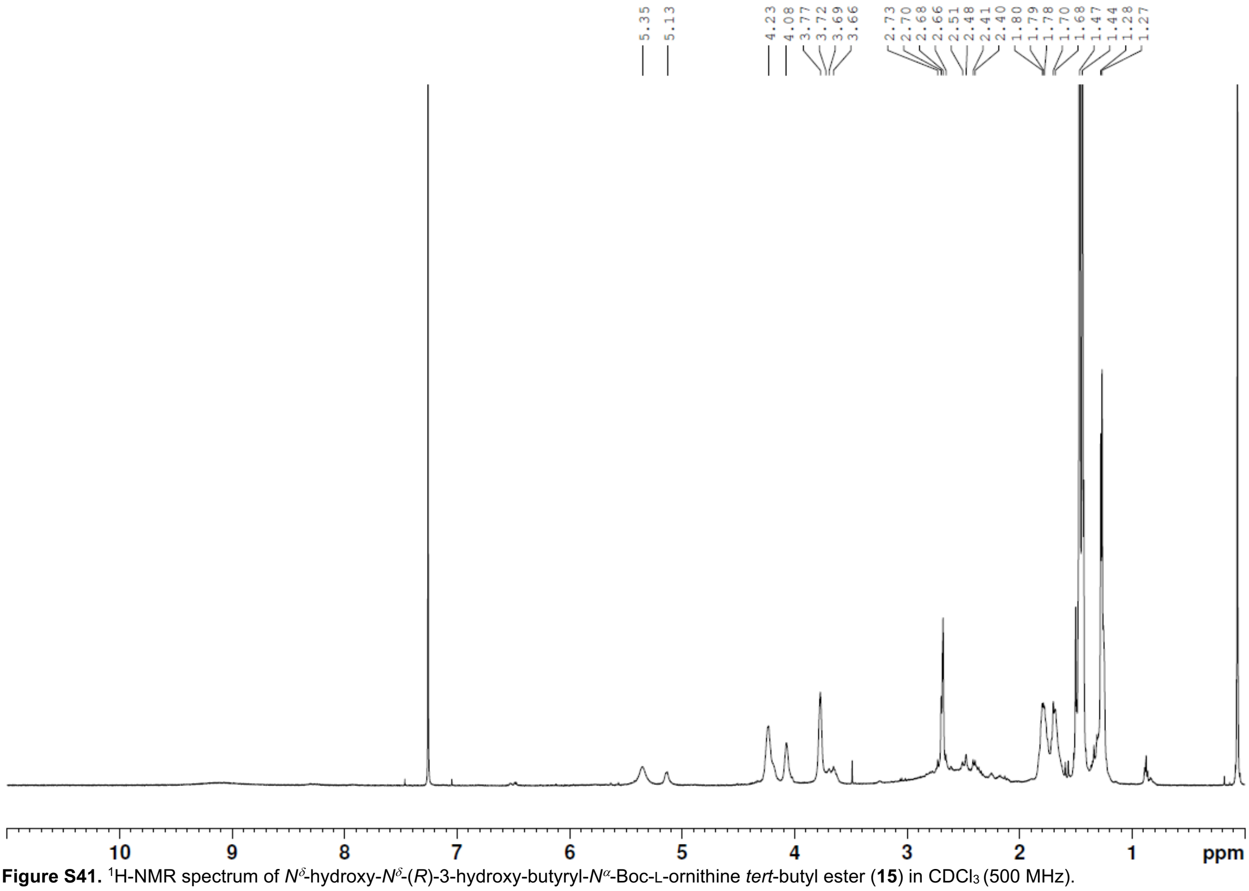


**Figure S41.** $^{1}$H-NMR spectrum of *N*$^{\delta}$-hydroxy-*N*$^{\delta}$-(*R*)-3-hydroxy-butyryl-*N*$^{\alpha}$-Boc-L-ornithine *tert*-butyl ester (**15**) in $CDCl_3$ (500 MHz).

**NMR spectra of $N^{\delta}$-hydroxy-$N^{\delta}$-(*R*)-3-hydroxy-butyryl-L-ornithine (6)**

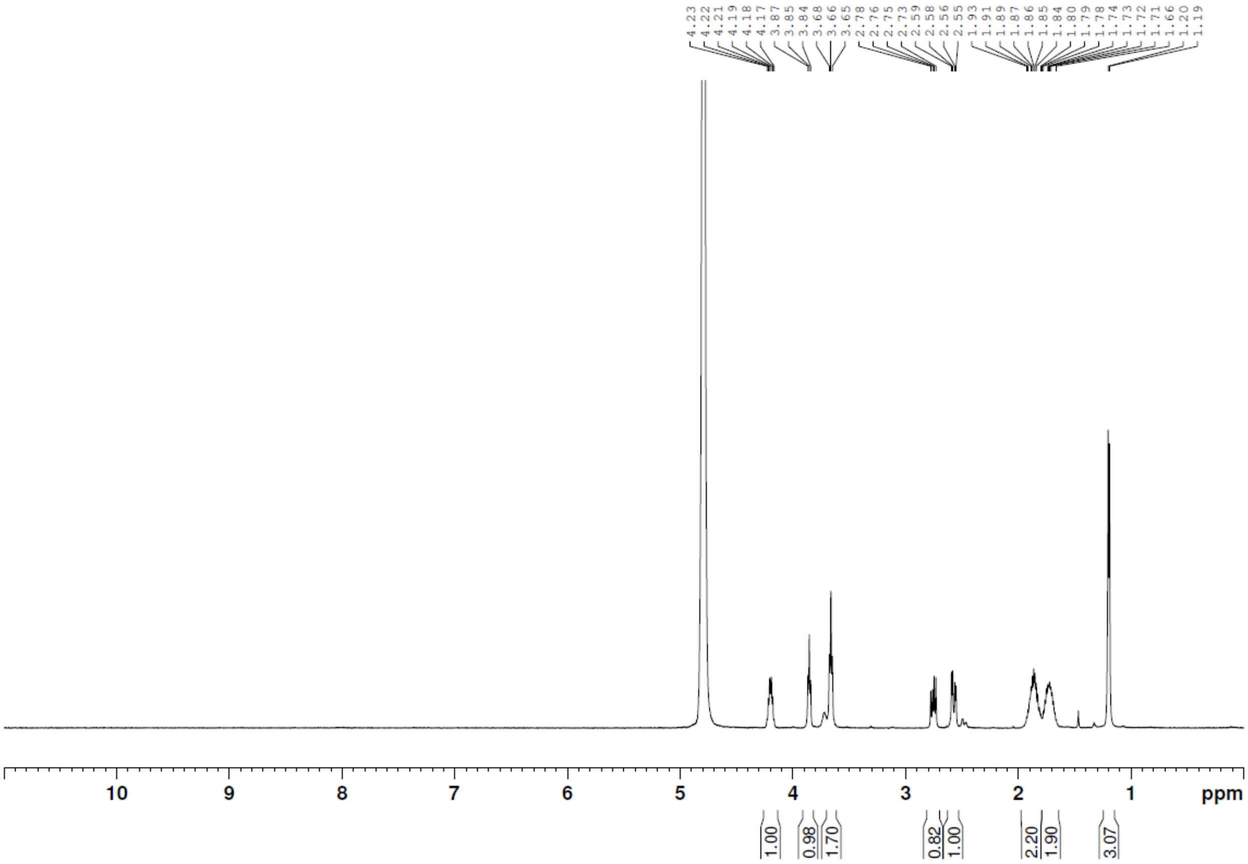


**Figure S42.** $^{1}$H-NMR spectrum of $N^{\delta}$-hydroxy-$N^{\delta}$-(*R*)-3-hydroxy-butyryl-L-ornithine (**6**) in $D_2O$ (500 MHz).

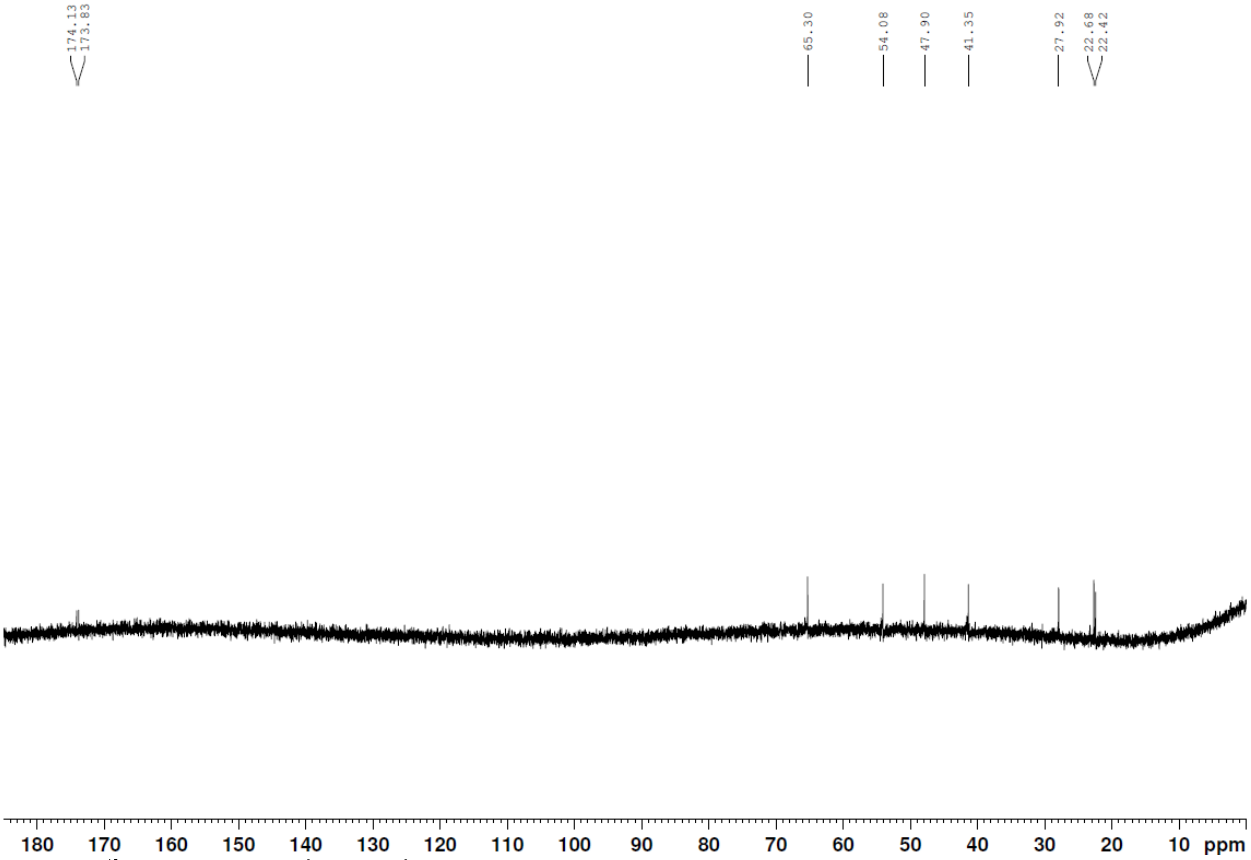


**Figure S43.** $^{13}$C-NMR spectrum of $N^{\delta}$-hydroxy-$N^{\delta}$-(*R*)-3-hydroxy-butyryl-L-ornithine (**6**) in $D_2O$ (125 MHz).

**NMR spectrum of 3-(*R*)-hydroxy-butyryl-CoA (7)**

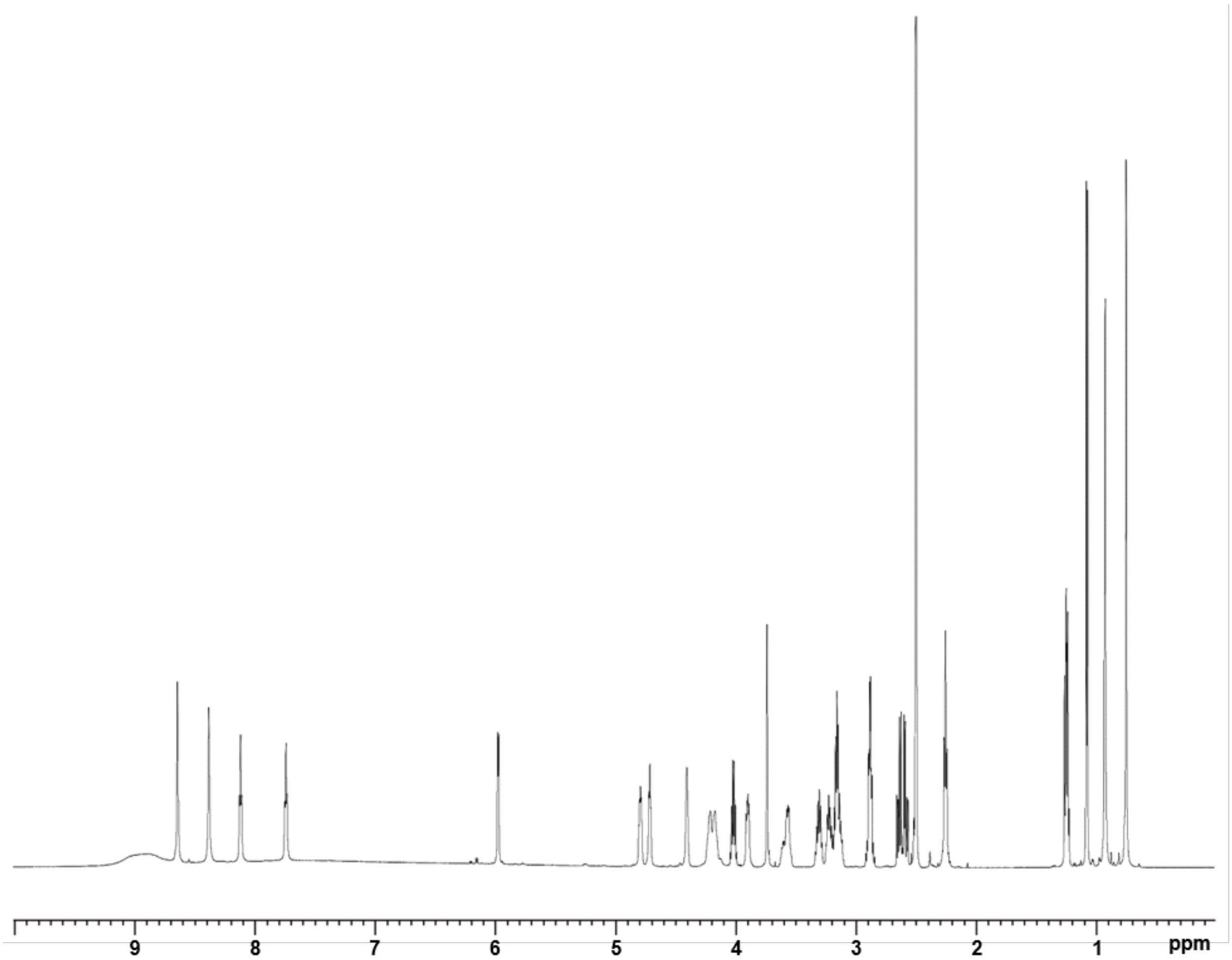


**Figure S44.** $^{1}H$ NMR spectrum of 3-(*R*)-hydroxy-butyryl-CoA (**7**) in DMSO-$d_6$ (600 MHz).

**NMR spectra of $Ga^{3+}$-pandorabactin A (8)**

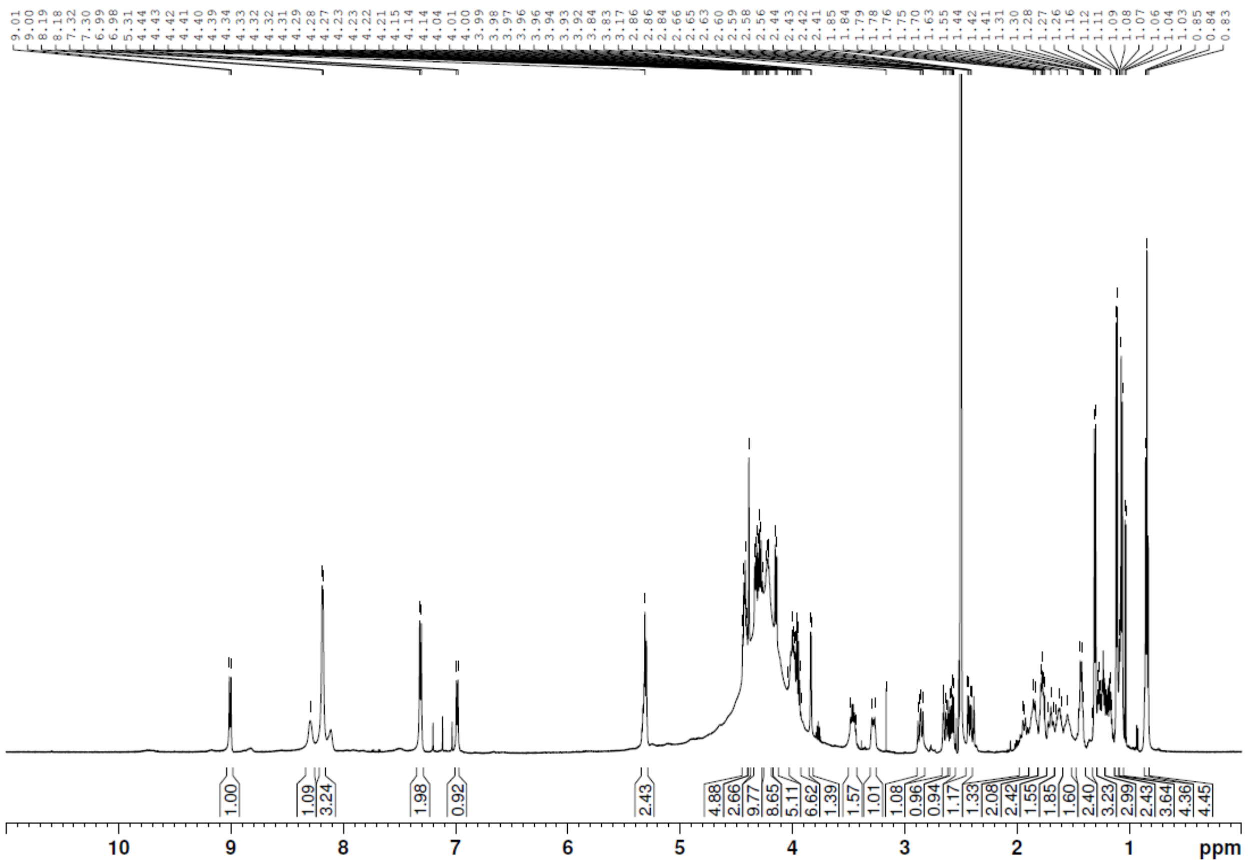


**Figure S45.** $^{1}H$-NMR spectrum of $Ga^{3+}$-pandorabactin A (**8**) in DMSO-$d_6$ (600 MHz).

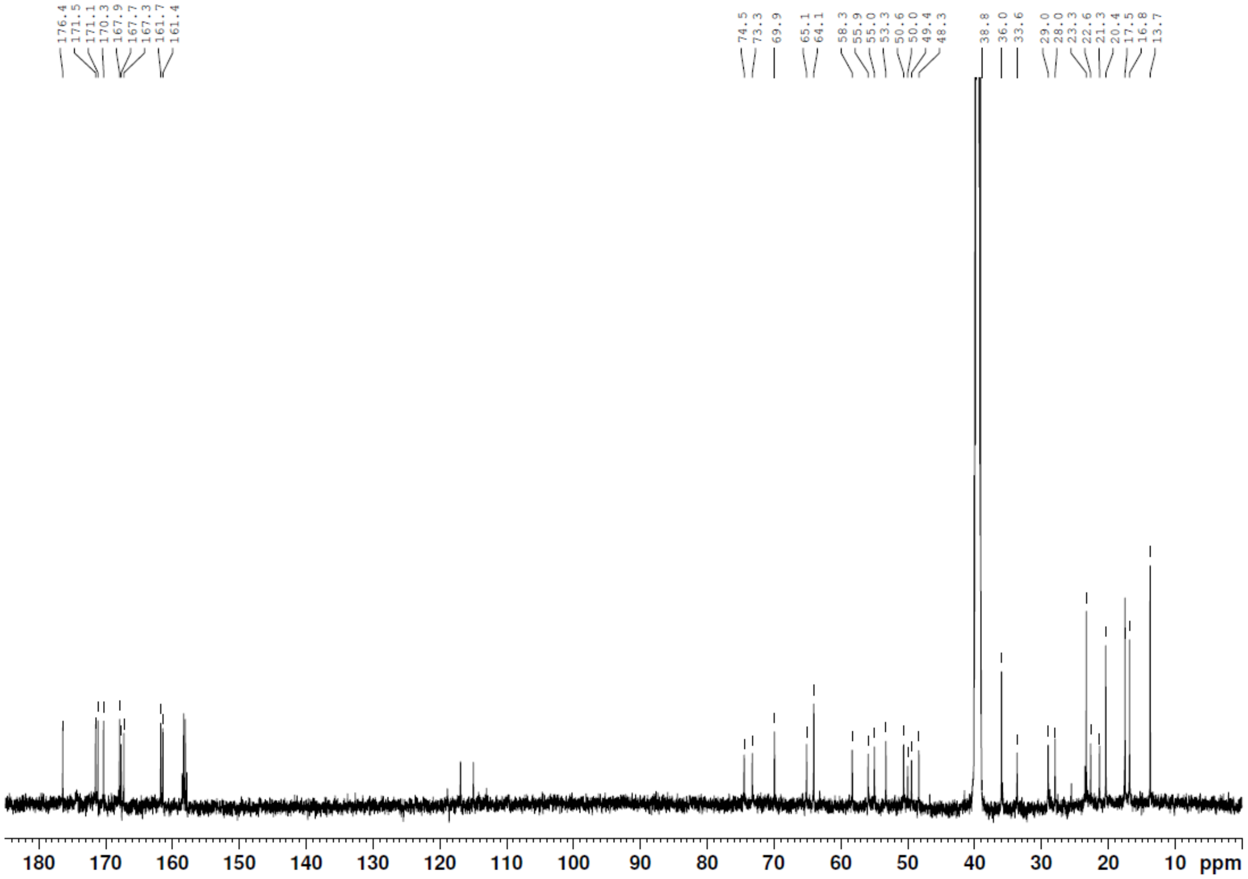


**Figure S46.** $^{13}C$-NMR spectrum of $Ga^{3+}$-pandorabactin A (**8**) in DMSO-$d_6$ (150 MHz).

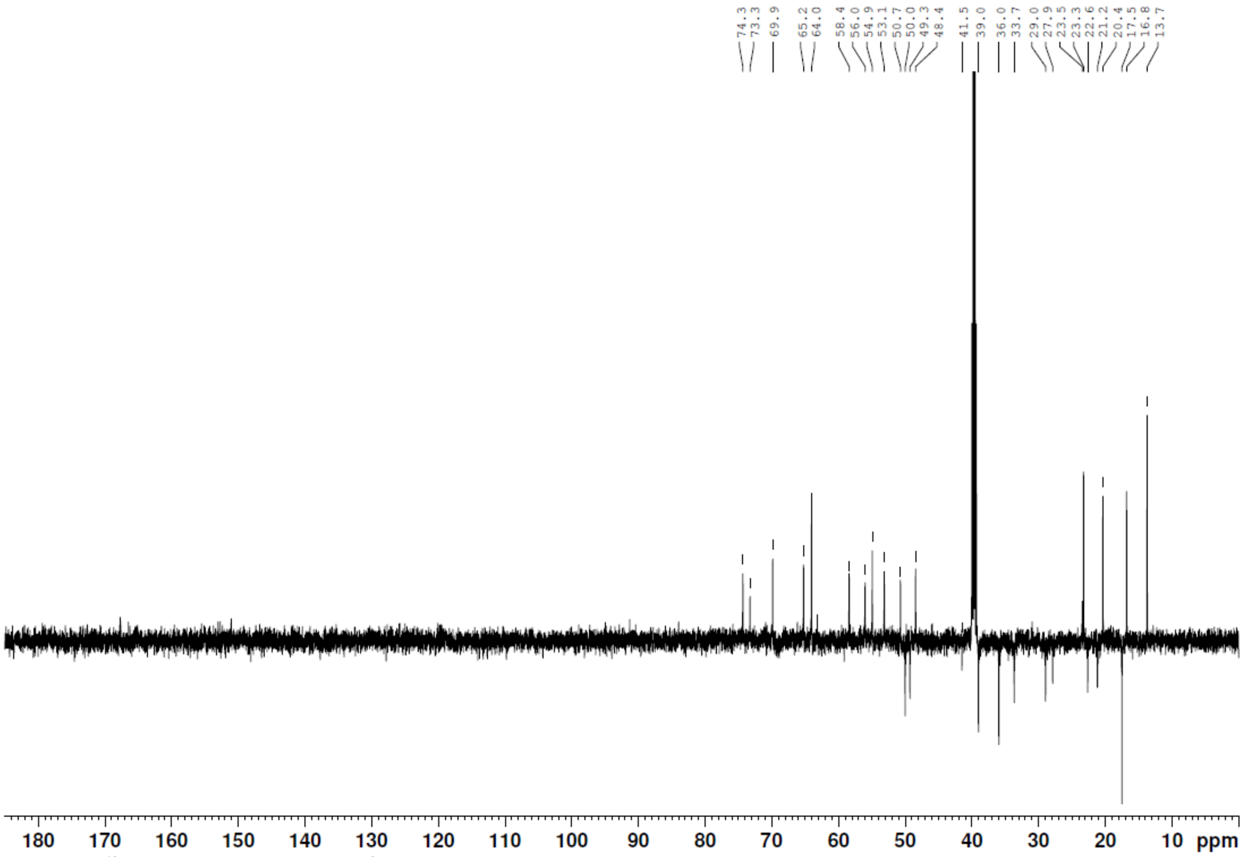


**Figure S47.** $^{13}C$-DEPT-NMR spectrum of $Ga^{3+}$-pandorabactin A (**8**) in DMSO-$d_6$ (150 MHz, $CH_2$ are negative, CH and $CH_3$ are positive).

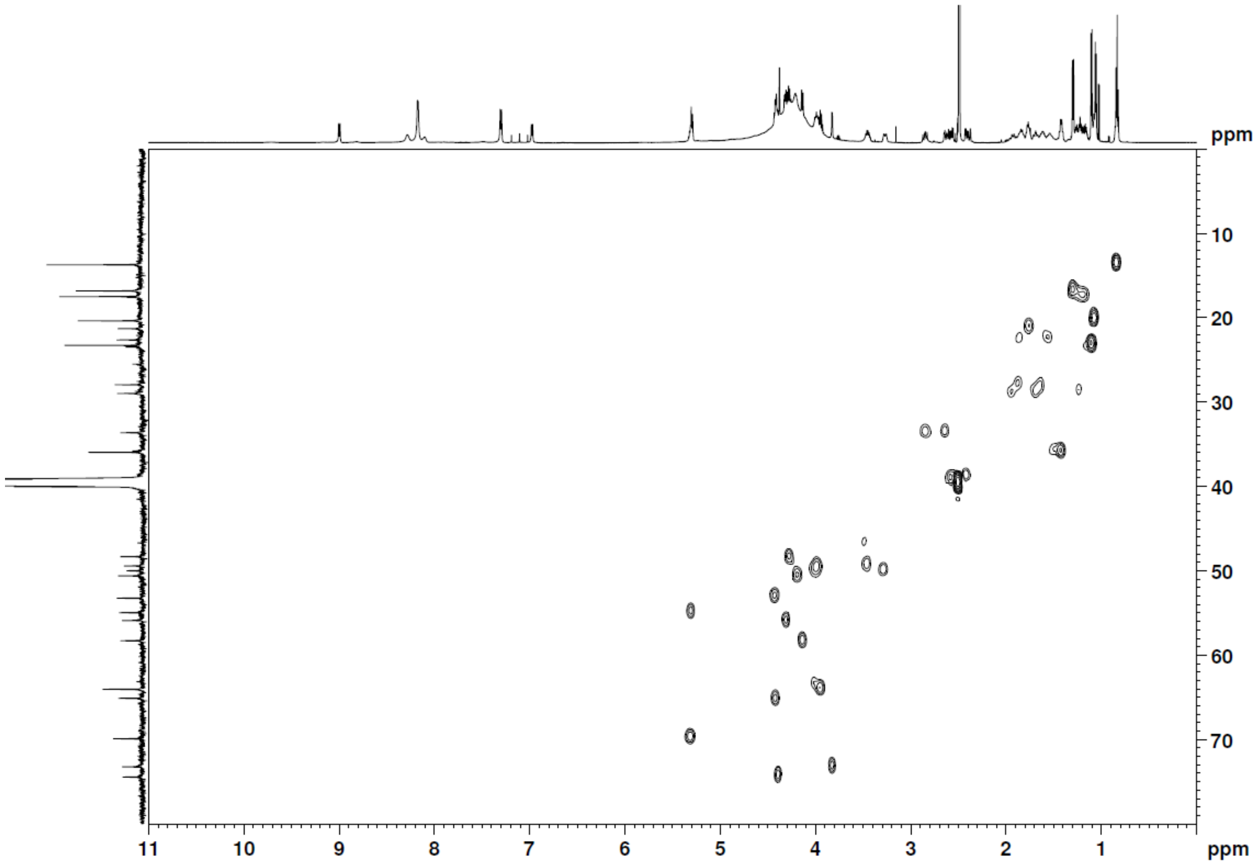


**Figure S48.** $^1H$-$^{13}C$-HSQC spectrum of $Ga^{3+}$-pandorabactin A (**8**) in DMSO-$d_6$ (600 MHz).

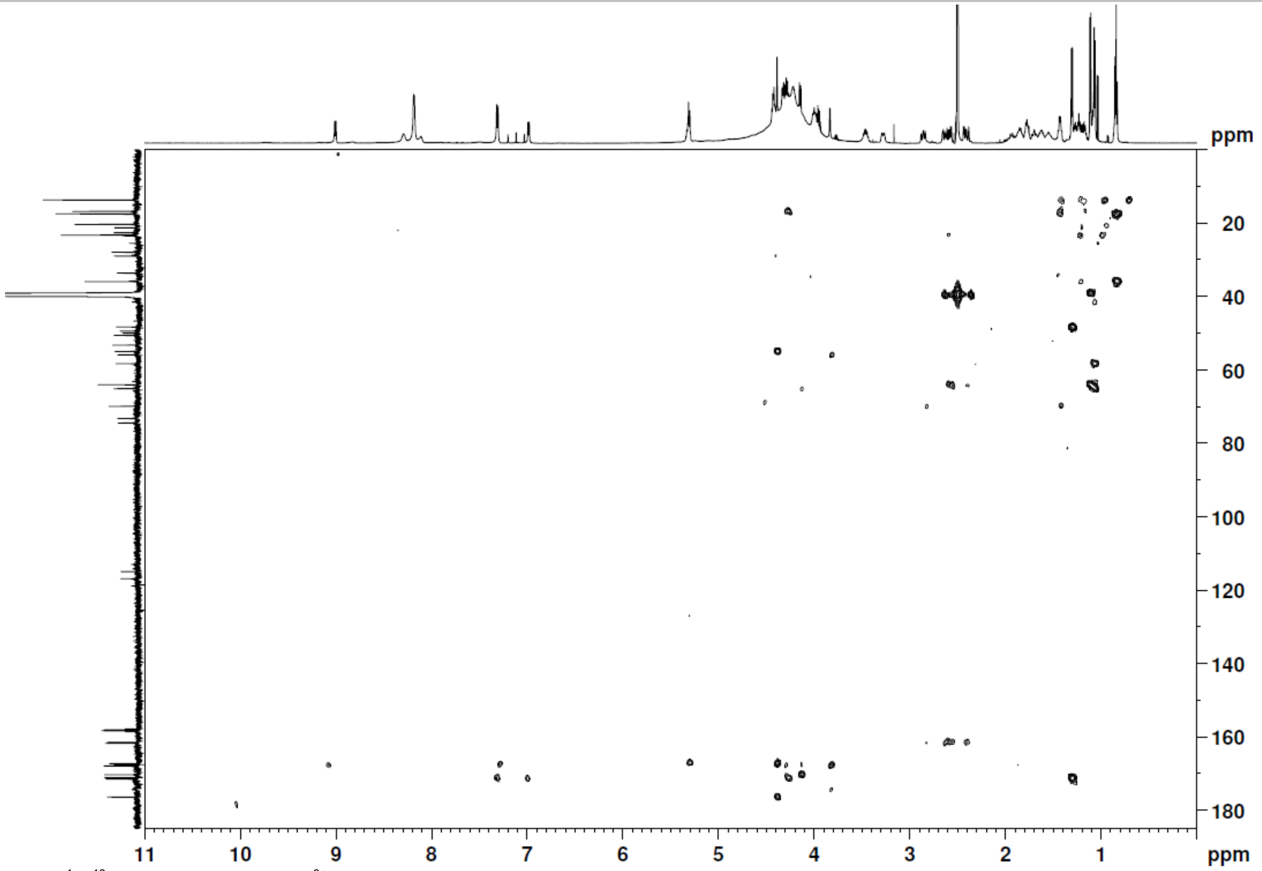


**Figure S49.** $^{1}H$-$^{13}C$-HMBC spectrum of $Ga^{3+}$-pandorabactin A (**8**) in DMSO-$d_6$ (600 MHz).

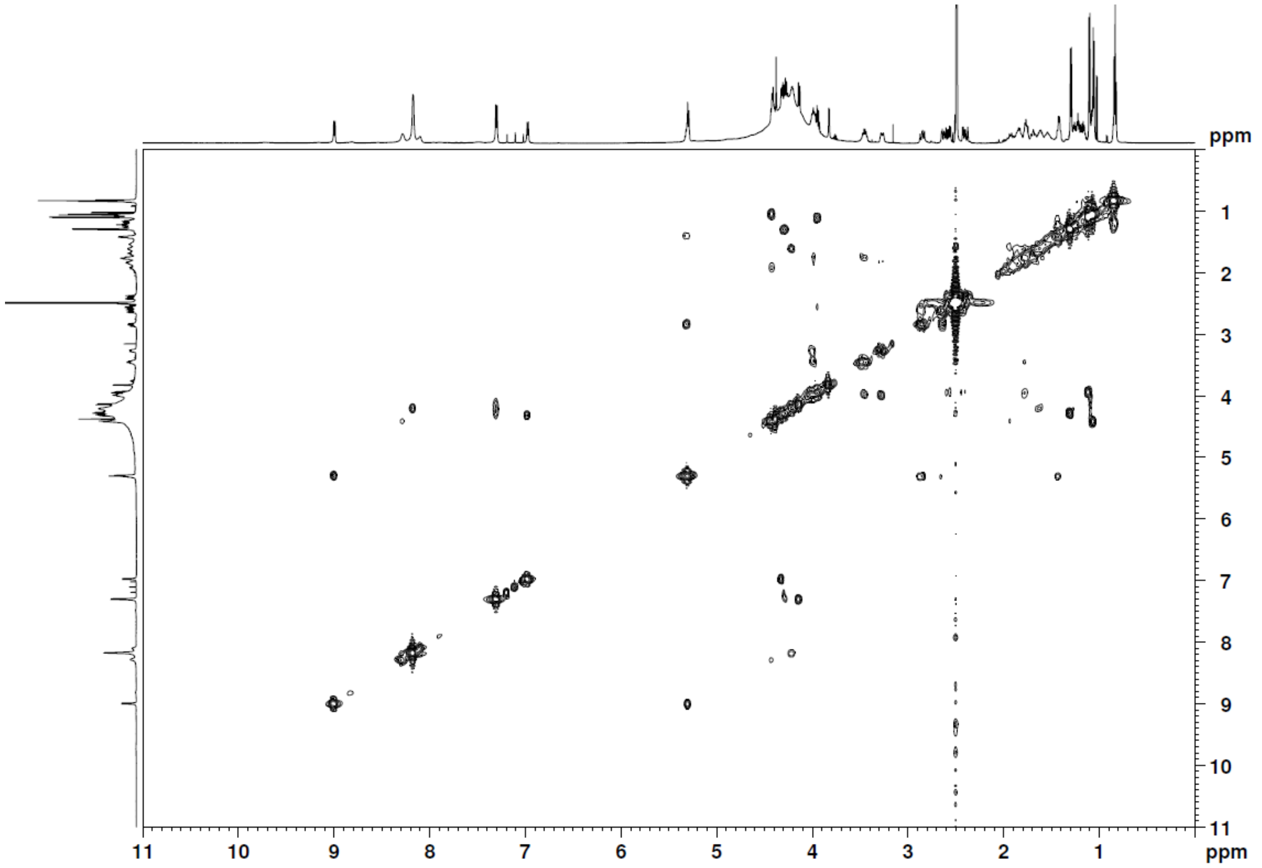


**Figure S50.** $^{1}H$-$^{1}H$-COSY spectrum of $Ga^{3+}$-pandorabactin A (**8**) in DMSO-$d_6$ (600 MHz).

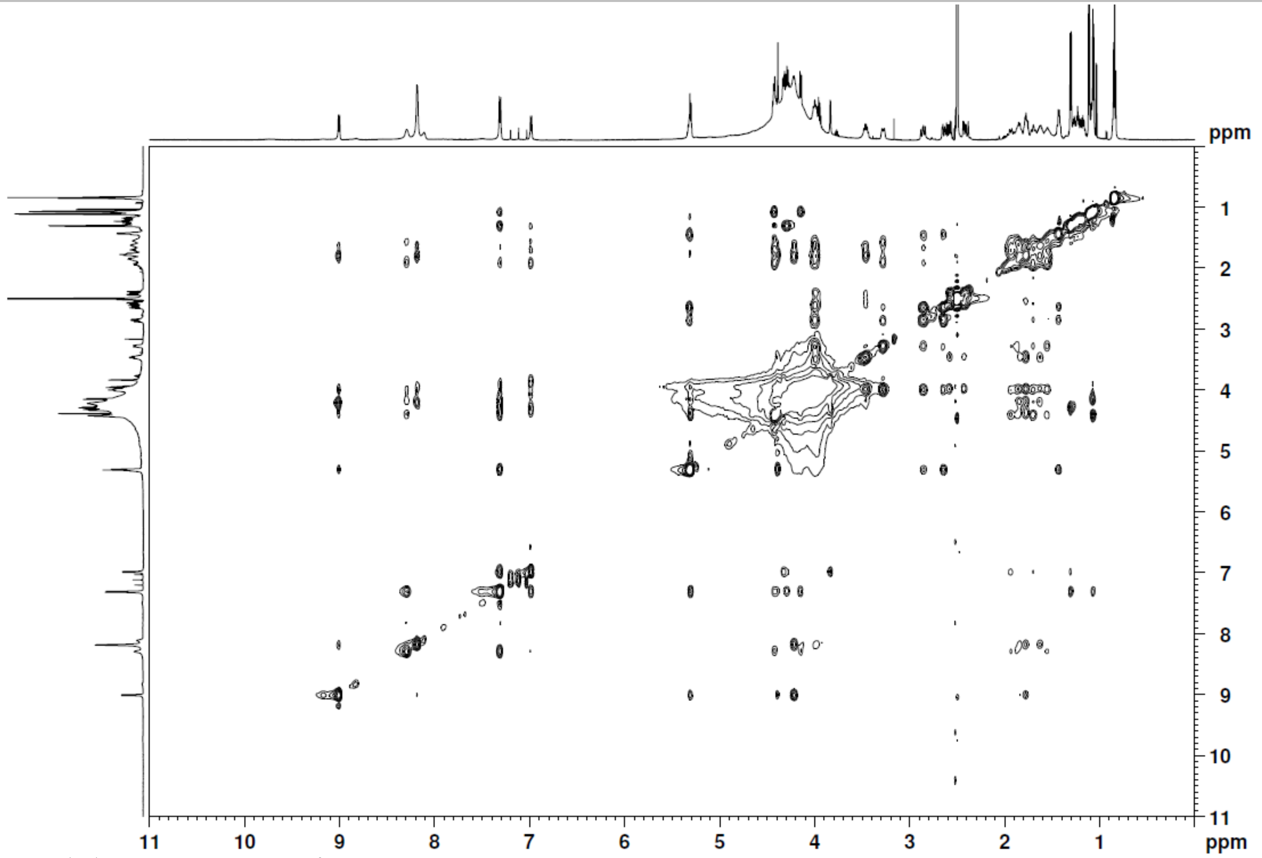


**Figure S51.** $^{1}H$-$^{1}H$-NOESY spectrum of $Ga^{3+}$-pandorabactin A (**8**) in DMSO-$d_6$ (600 MHz).

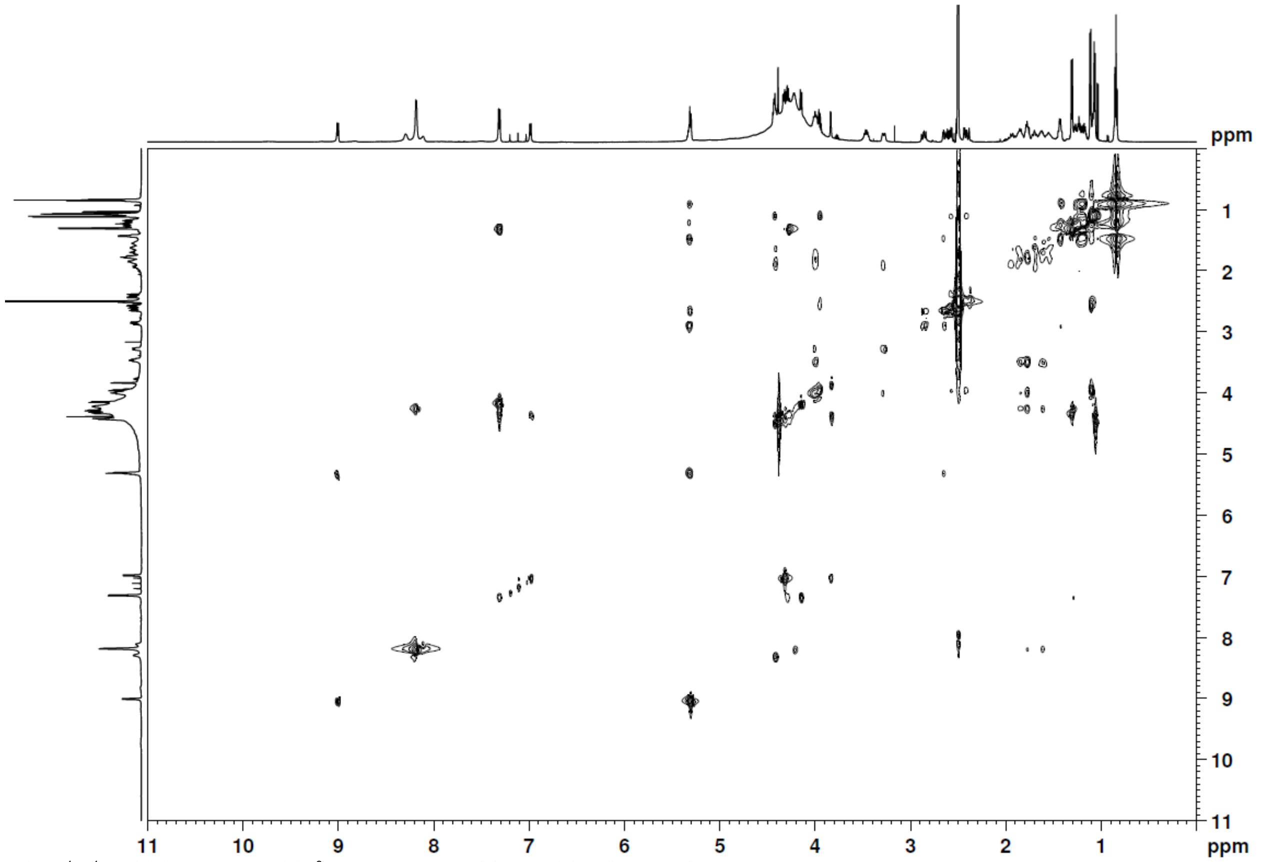


**Figure S52.** $^{1}H$-$^{1}H$-TOCSY spectrum of $Ga^{3+}$-pandorabactin A (**8**) in DMSO-$d_6$ (600 MHz).

**NMR spectra of $Ga^{3+}$-pandorabactin B (9)**

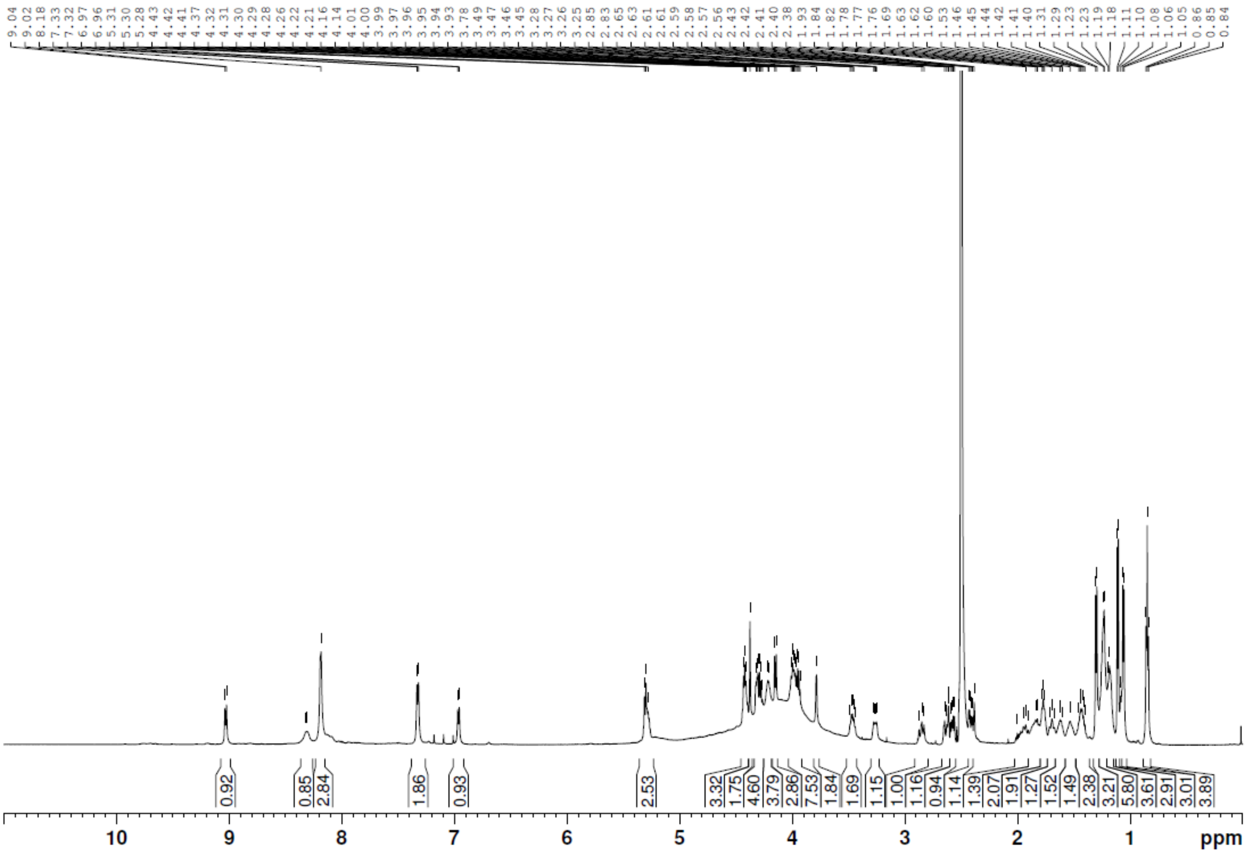

**Figure S53.** $^{1}H$-NMR spectrum of $Ga^{3+}$-pandorabactin B (**9**) in DMSO-$d_6$ (600 MHz).

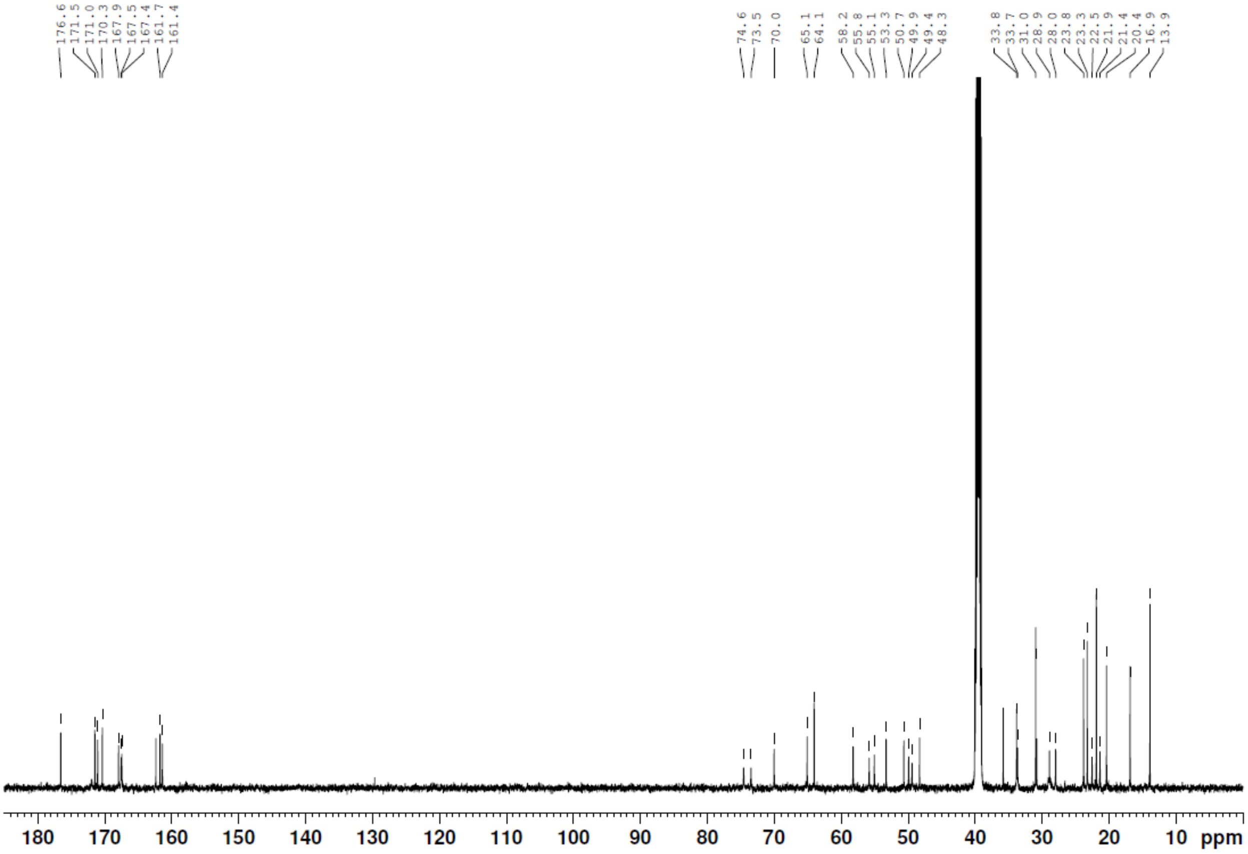

**Figure S54.** $^{13}C$-NMR spectrum of $Ga^{3+}$-pandorabactin B (**9**) in DMSO-$d_6$ (150 MHz).

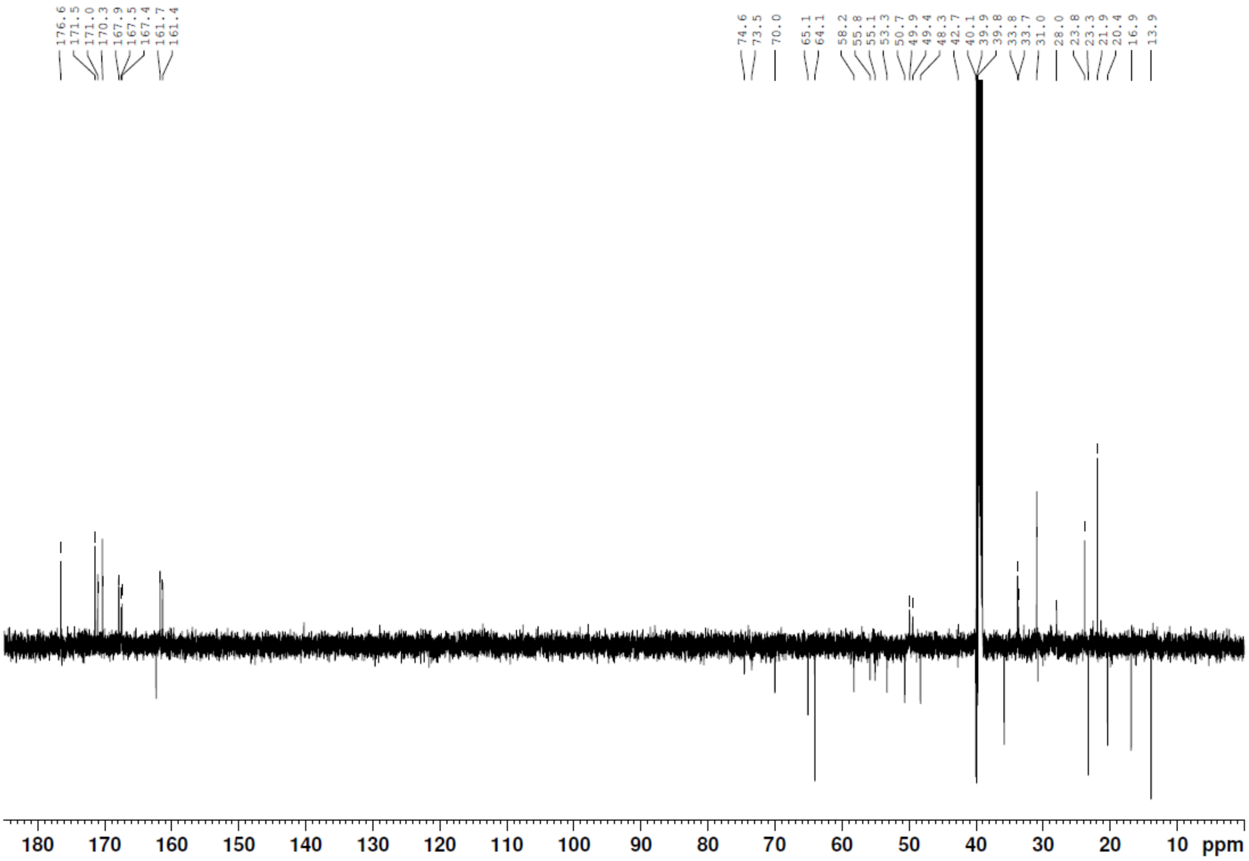


**Figure S55.** $^{13}C$-Jmod-NMR spectrum of $Ga^{3+}$-pandorabactin B (**9**) in DMSO-$d_6$ (150 MHz, C and $CH_2$ are positive, CH and $CH_3$ are negative).

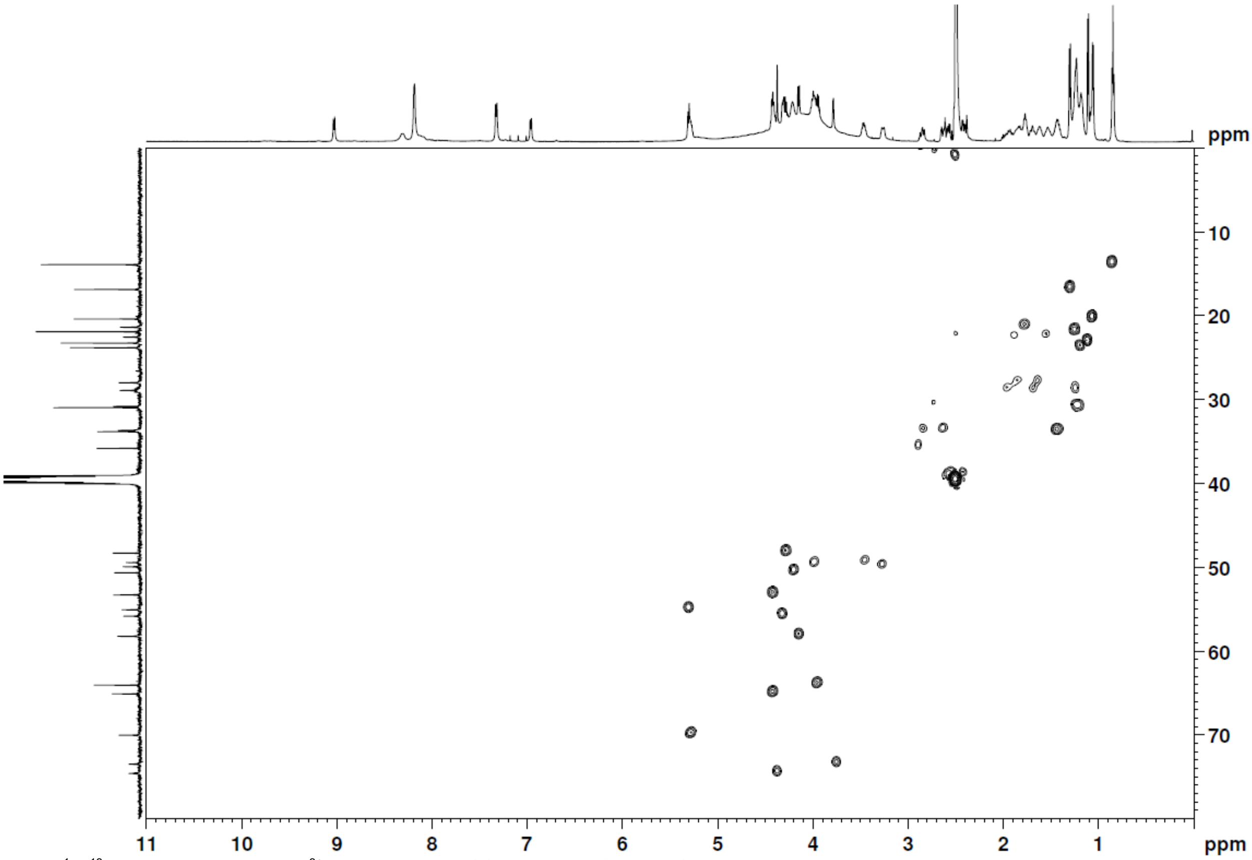


**Figure S56.** $^{1}H$-$^{13}C$-HSQC spectrum of $Ga^{3+}$-pandorabactin B (**9**) in DMSO-$d_6$ (600 MHz).

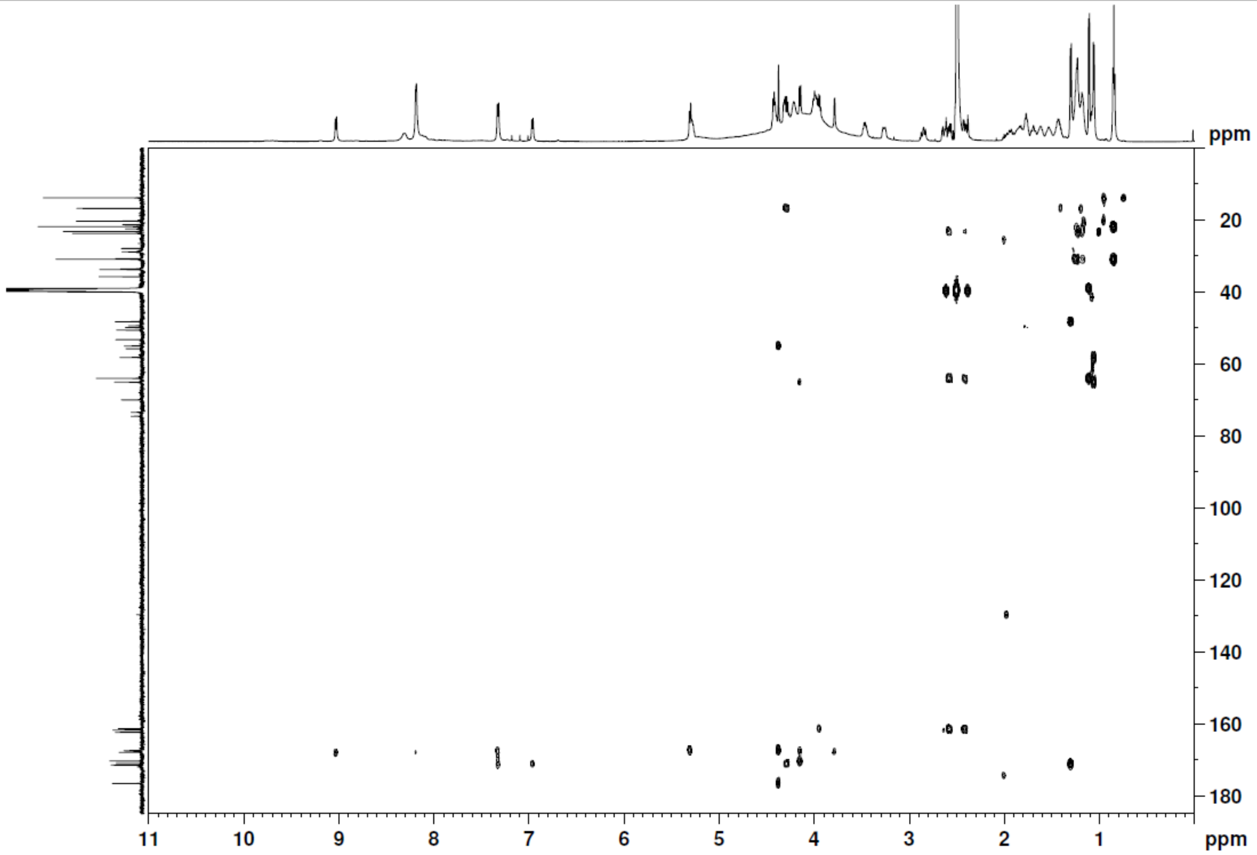


**Figure S57.** $^{1}H$-$^{13}C$-HMBC spectrum of $Ga^{3+}$-pandorabactin B (**9**) in DMSO-$d_6$ (600 MHz).

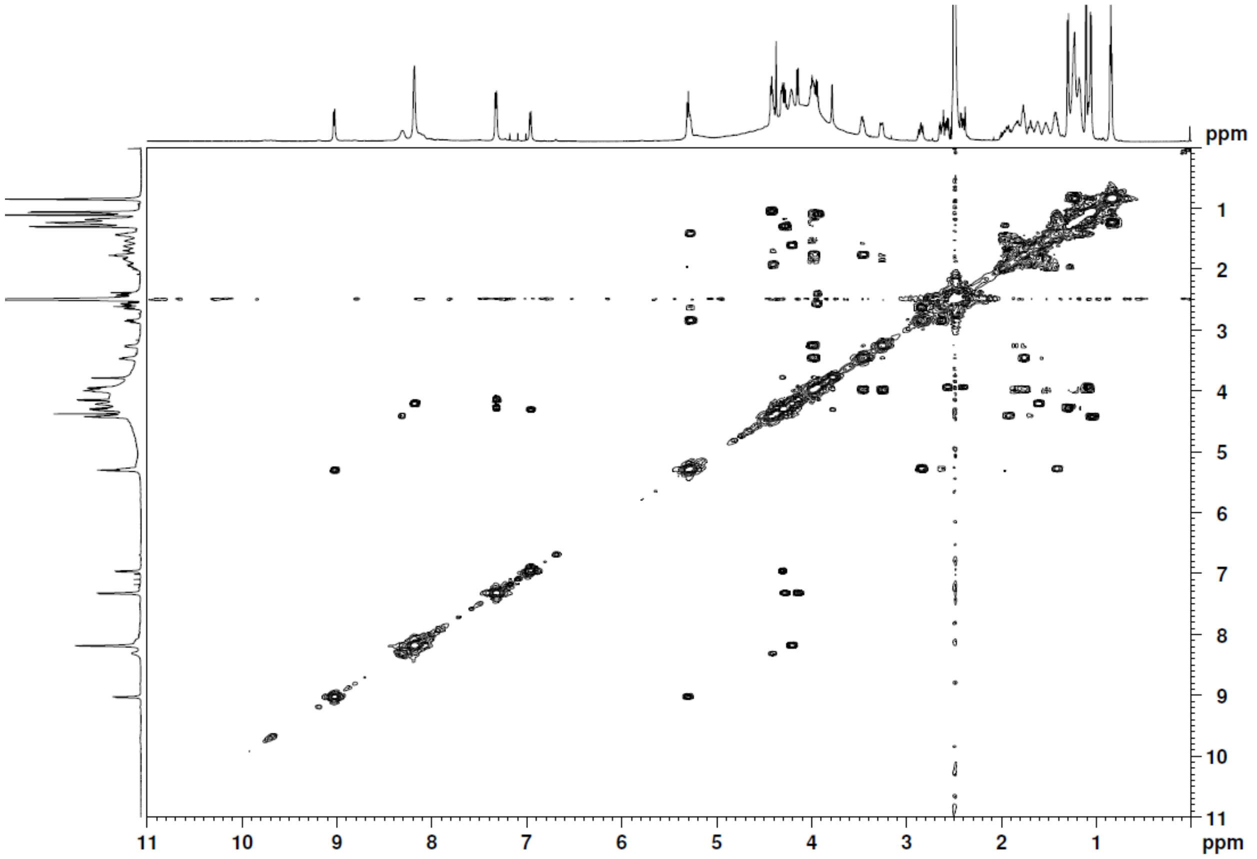


**Figure S58.** $^{1}H$-$^{1}H$-COSY spectrum of $Ga^{3+}$-pandorabactin B (**9**) in DMSO-$d_6$ (600 MHz).

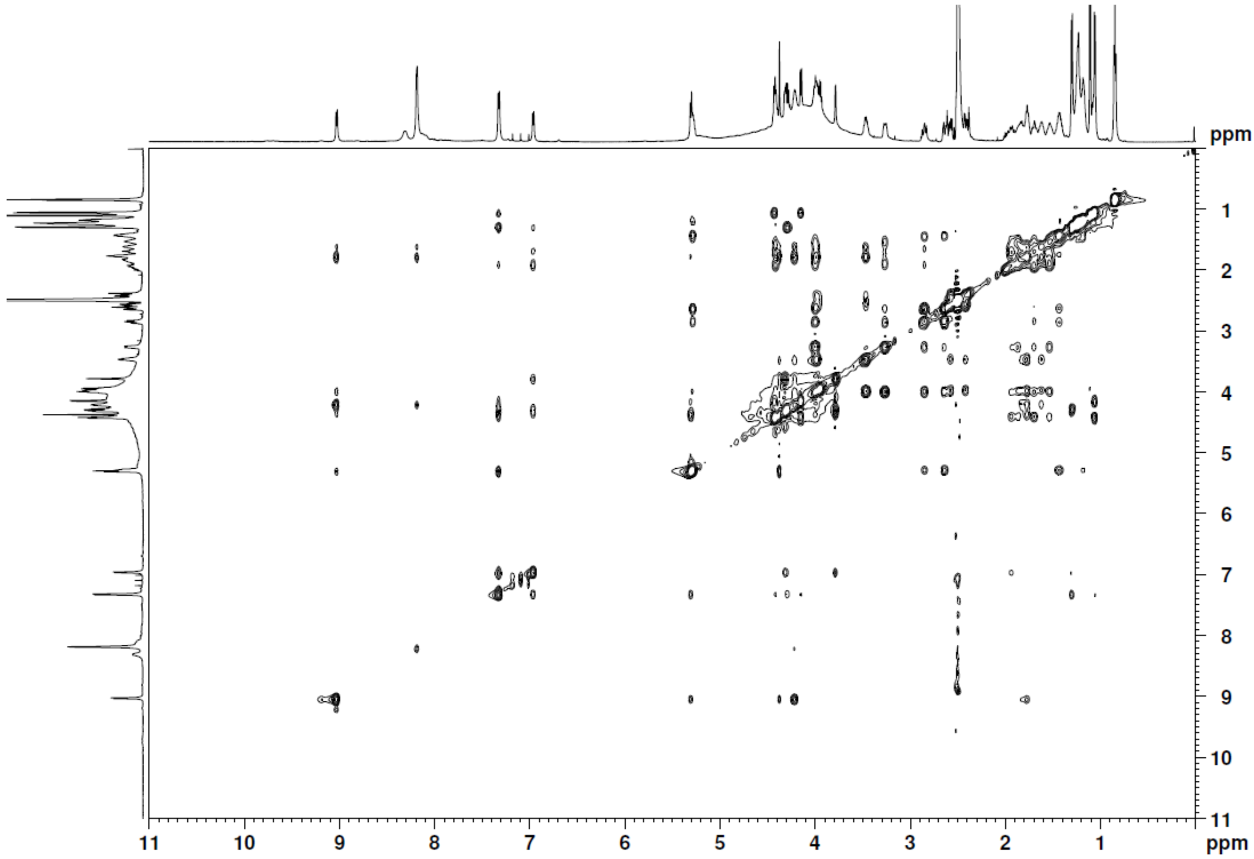


**Figure S59.** $^{1}H$-$^{1}H$-NOESY spectrum of $Ga^{3+}$-pandorabactin B (**9**) in DMSO-$d_6$ (600 MHz).

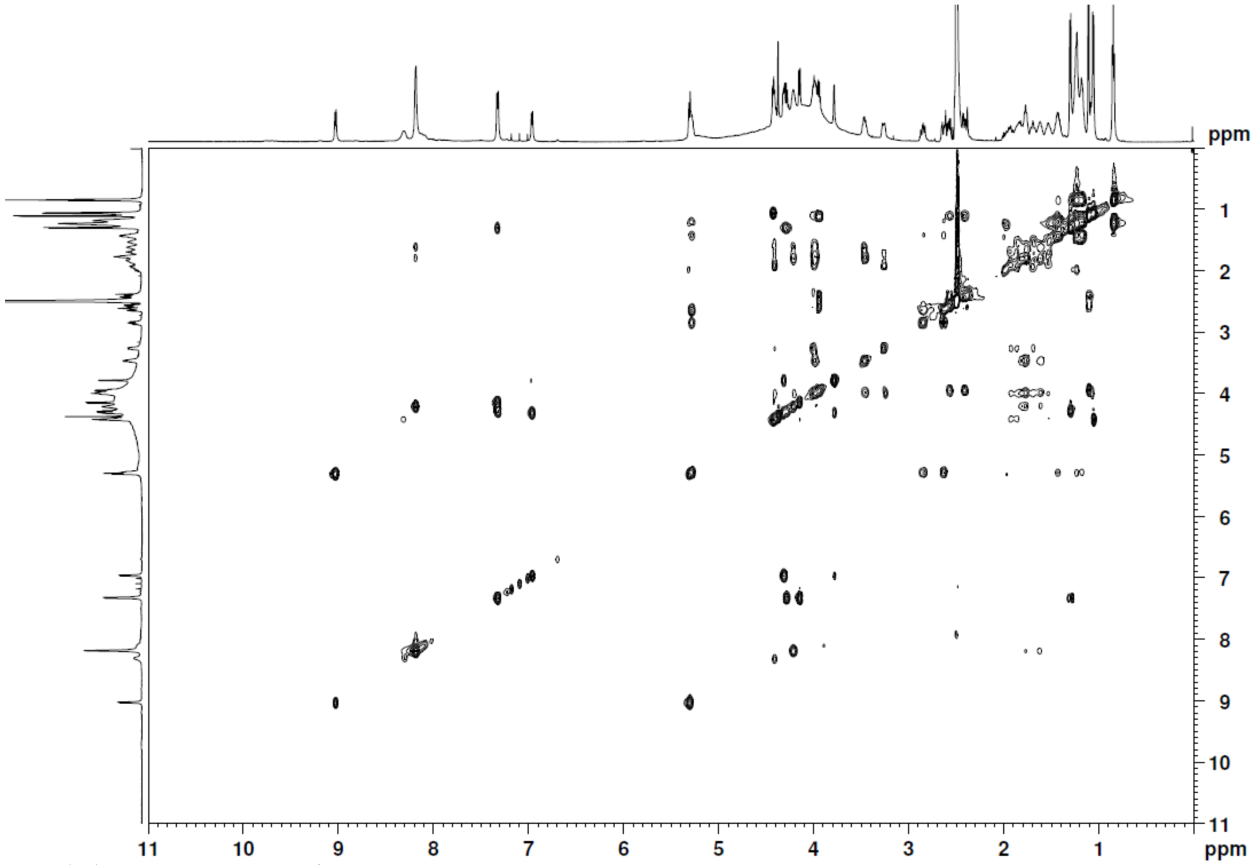


**Figure S60.** $^{1}H$-$^{1}H$-TOCSY spectrum of $Ga^{3+}$-pandorabactin B (**9**) in DMSO-$d_6$ (600 MHz).

## References


[1] L. J. Liermann, A. S. Barnes, B. E. Kalinowski, X. Zhou, S. L. Brantley, "Microenvironments of pH in biofilms grown on dissolving silicate surfaces" *Chem. Geol.* **2000**, *171*, 1.
[2] R. Hermenau, J. L. Mehl, K. Ishida, B. Dose, S. J. Pidot, T. P. Stinear, C. Hertweck, "Genomics-Driven Discovery of NO-Donating Diazeniumdiolate Siderophores in Diverse Plant-Associated Bacteria" *Angew. Chem. Int. Ed. Engl.* **2019**, *58*, 13024.
[3] R. Hermenau, J. L. Mehl, K. Ishida, B. Dose, S. J. Pidot, T. P. Stinear, C. Hertweck, "Genomics‐Driven Discovery of NO‐Donating Diazeniumdiolate Siderophores in Diverse Plant‐Associated Bacteria" *Angew. Chem.* **2019**, *131*, 13158.
[4] K. Blin, S. Shaw, H. E. Augustijn, Z. L. Reitz, F. Biermann, M. Alanjary, A. Fetter, B. R. Terlouw, W. W. Metcalf, E. J. N. Helfrich, G. P. van Wezel, M. H. Medema, T. Weber, "antiSMASH 7.0: new and improved predictions for detection, regulation, chemical structures and visualisation" *Nucleic Acids Res.* **2023**, *51*, W46.
[5] S. F. Altschul, W. Gish, W. Miller, E. W. Myers, D. J. Lipman, "Basic local alignment search tool" *J. Mol. Biol.* **1990**, *215*, 403.
[6] B. O. Bachmann, J. Ravel, "Chapter 8 Methods for In Silico Prediction of Microbial Polyketide and Nonribosomal Peptide Biosynthetic Pathways from DNA Sequence Data" *Methods Enzymol.* **2009**, *458*, 181.
[7] M. H. Mirhakkak, X. Chen, Y. Ni, T. Heinekamp, T. Sae-Ong, L. L. Xu, O. Kurzai, A. E. Barber, A. A. Brakhage, S. Boutin, S. Schauble, G. Panagiotou, "Genome-scale metabolic modeling of *Aspergillus fumigatus* strains reveals growth dependencies on the lung microbiome" *Nat. Commun.* **2023**, *14*, 4369.
[8] K. Fujii, Y. Ikai, T. Mayumi, H. Oka, M. Suzuki, K.-i. Harada, "A Nonempirical Method Using LC/MS for Determination of the Absolute Configuration of Constituent Amino Acids in a Peptide: Elucidation of Limitations of Marfey's Method and of Its Separation Mechanism" *Anal. Chem.* **1997**, *69*, 3346.
[9] L. J. Klau, S. Podell, K. E. Creamer, A. M. Demko, H. W. Singh, E. E. Allen, B. S. Moore, N. Ziemert, A. C. Letzel, P. R. Jensen, "The Natural Product Domain Seeker version 2 (NaPDoS2) webtool relates ketosynthase phylogeny to biosynthetic function" *J. Biol. Chem.* **2022**, *298*, 102480.
[10] K. Katoh, J. Rozewicki, K. D. Yamada, "MAFFT online service: multiple sequence alignment, interactive sequence choice and visualization" *Brief. Bioinform.* **2019**, *20*, 1160.
[11] S. Kuraku, C. M. Zmasek, O. Nishimura, K. Katoh, "aLeaves facilitates on-demand exploration of metazoan gene family trees on MAFFT sequence alignment server with enhanced interactivity" *Nucleic Acids Res.* **2013**, *41*, W22.
[12] J. Trifinopoulos, L. T. Nguyen, A. von Haeseler, B. Q. Minh, "W-IQ-TREE: a fast online phylogenetic tool for maximum likelihood analysis" *Nucleic Acids Res.* **2016**, *44*, W232.
[13] S. Kumar, G. Stecher, K. Tamura, "MEGA7: Molecular Evolutionary Genetics Analysis Version 7.0 for Bigger Datasets" *Mol. Biol. Evol.* **2016**, *33*, 1870.
[14] K. Graupner, K. Scherlach, T. Bretschneider, G. Lackner, M. Roth, H. Gross, C. Hertweck, "Imaging Mass Spectrometry and Genome Mining Reveal Highly Antifungal Virulence Factor of Mushroom Soft Rot Pathogen" *Angew. Chem. Int. Ed.* **2012**, *51*, 13173.
[15] K. Graupner, K. Scherlach, T. Bretschneider, G. Lackner, M. Roth, H. Gross, C. Hertweck, "Imaging Mass Spectrometry and Genome Mining Reveal Highly Antifungal Virulence Factor of Mushroom Soft Rot Pathogen" *Angew. Chem.* **2012**, *124*, 13350.
[16] R. Hermenau, K. Ishida, S. Gama, B. Hoffmann, M. Pfeifer-Leeg, W. Plass, J. F. Mohr, T. Wichard, H. P. Saluz, C. Hertweck, "Gramibactin is a bacterial siderophore with a diazeniumdiolate ligand system" *Nat. Chem. Biol.* **2018**, *14*, 841.
[17] J. R. Heemstra, Jr., C. T. Walsh, E. S. Sattely, "Enzymatic tailoring of ornithine in the biosynthesis of the *Rhizobium* cyclic trihydroxamate siderophore vicibactin" *J. Am. Chem. Soc.* **2009**, *131*, 15317.
[18] J. C. Williams, J. R. Sheldon, H. D. Imlay, B. F. Dutter, M. M. Draelos, E. P. Skaar, G. A. Sulikowski, "Synthesis of the Siderophore Coelichelin and Its Utility as a Probe in the Study of Bacterial Metal Sensing and Response" *Org. Lett.* **2019**, *21*, 679.
[19] B. Schwyn, J. B. Neilands, "Universal chemical assay for the detection and determination of siderophores" *Anal. Biochem.* **1987**, *160*, 47.
[20] B. C. Louden, D. Haarmann, A. M. Lynne, "Use of Blue Agar CAS Assay for Siderophore Detection" *J. Microbiol. Biol. Educ.* **2011**, *12*, 51.
[21] *DIN 58940-8 Beiblatt 1:1991-07 Medical microbiology - Methods for the determination of susceptibility of pathogens (except mycrobacteria) to antimicrobial agents - MICs of agents with control strains for the microdilution*, Beuth, Berlin, **1991**.
[22] J. A. Waitz, S. National Committee for Clinical Laboratory, *Methods for dilution antimicrobial susceptibility tests for bacteria that grow aerobically*, Second edition ed., National Committee for Clinical Laboratory Standards Villanova, Pennsylvania, Villanova, Pennsylvania, **1991**.
[23] F. Trottmann, J. Franke, K. Ishida, M. Garcia-Altares, C. Hertweck, "A Pair of Bacterial Siderophores Releases and Traps an Intercellular Signal Molecule: An Unusual Case of Natural Nitrone Bioconjugation" *Angew. Chem. Int. Ed.* **2019**, *58*, 200.
[24] F. Trottmann, J. Franke, K. Ishida, M. García‐Altares, C. Hertweck, "A Pair of Bacterial Siderophores Releases and Traps an Intercellular Signal Molecule: An Unusual Case of Natural Nitrone Bioconjugation" *Angew. Chem.* **2019**, *131*, 206.
[25] S. Tortorella, P. Tiberi, A. P. Bowman, B. S. R. Claes, K. Scupakova, R. M. A. Heeren, S. R. Ellis, G. Cruciani, "LipostarMSI: Comprehensive, Vendor-Neutral Software for Visualization, Data Analysis, and Automated Molecular Identification in Mass Spectrometry Imaging" *J. Am. Soc. Mass Spectrom.* **2020**, *31*, 155.
[26] B. Buchfink, C. Xie, D. H. Huson, "Fast and sensitive protein alignment using DIAMOND" *Nat. Methods* **2015**, *12*, 59.
[27] M. I. Love, W. Huber, S. Anders, "Moderated estimation of fold change and dispersion for RNA-seq data with DESeq2" *Genome Biol.* **2014**, *15*, 550.
